# Facile synthesis of α-alkoxyl amides *via* scandium-catalyzed oxidative reaction between ynamides and alcohols†

**DOI:** 10.1039/c8ra03842b

**Published:** 2018-05-18

**Authors:** Zhi-Xin Zhang, Bo-Han Zhu, Pei-Xi Xie, Jia-Qi Tang, Xin-Ling Li, Chunyin Zhu, Ying-Wu Yin, Long-Wu Ye

**Affiliations:** State Key Laboratory of Physical Chemistry of Solid Surfaces, Key Laboratory for Chemical Biology of Fujian Province, College of Chemistry and Chemical Engineering, Xiamen University Xiamen 361005 China longwuye@xmu.edu.cn; School of Chemistry and Chemical Engineering, Jiangsu University Zhenjiang 212013 China zhucycn@gmail.com; State Key Laboratory of Organometallic Chemistry, Shanghai Institute of Organic Chemistry, Chinese Academy of Sciences Shanghai 200032 China

## Abstract

A novel and efficient scandium-catalyzed oxidative reaction between ynamides and alcohols for the facile synthesis of various α-alkoxyl amides is reported in this paper. The reaction avoids the need for the use of α-diazo carbonyls which are unstable and may cause some safety concerns. Instead, by using alkynes as the starting materials, this protocol features readily available substrates, compatibility with a broad range of functional groups, simple procedure, mild reaction conditions, and high chemoselectivity.

## Abstract 2

A novel scandium-catalyzed oxidative reaction between ynamides and alcohols has been developed, allowing the concise and efficient synthesis of various substituted α-alkoxyl amides.

## Graphical abstract

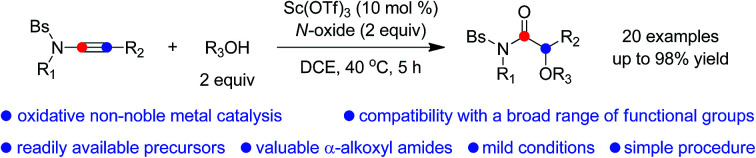

## Introduction

**Fig. 1:**
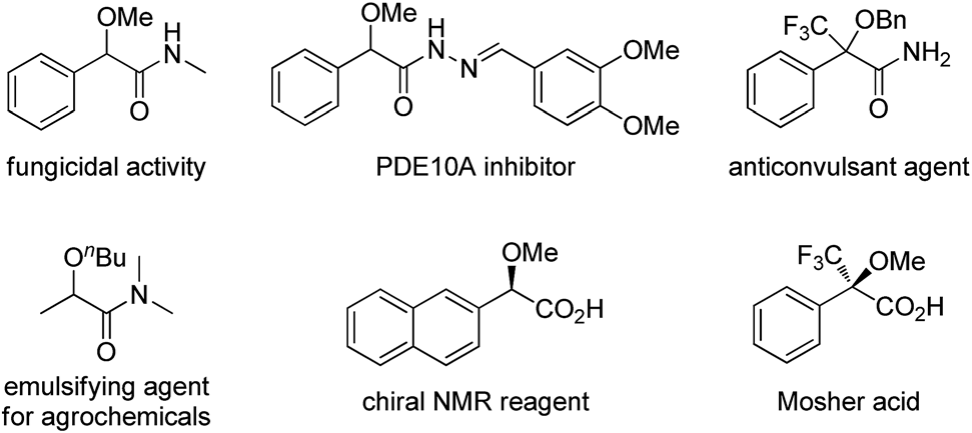
Selected examples of bioactive α-alkoxyl carbonyls.

**Scheme 1:**
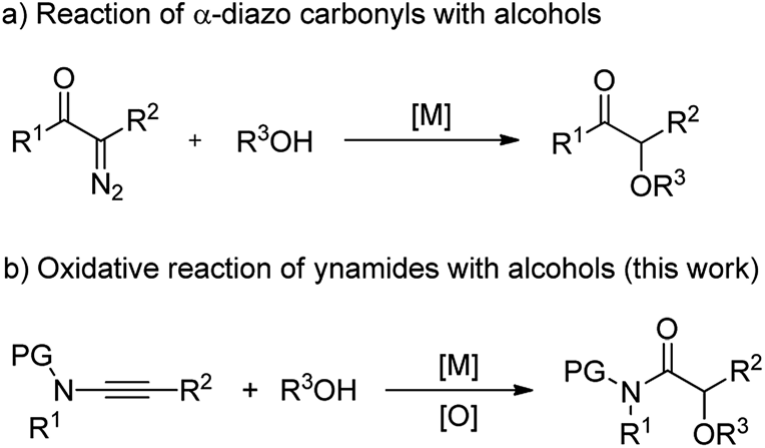
Two synthetic pathways to α-alkoxyl carbonyls: the reaction of alcohols with α-diazo carbonyls and ynamides.

α-Alkoxyl carbonyls are a class of privileged motifs prevalent in many important natural products, pharmaceuticals, and agrochemicals (Fig. 1).^1^ Accordingly, significant efforts have been devoted to their synthesis. In this context, α-functionalization of carbonyl compounds such as the transition metal-catalyzed insertion of α-diazo carbonyl into O–H bond (Scheme 1a),^2,3^ has been established as one of the most important methods to construct these structures. However, those reactions involving α-diazo carbonyls generally suffer from the problems of inaccessible precursors, limitations on the substrate scope, and multistep synthesis.^2^ Moreover, the preparation of α-alkoxyl amides has been less explored and documented than that of α-alkoxyl ketones and esters.^4^ Thus, to develop highly efficient strategies to access valuable α-alkoxyl amides is of great importance.

Alkynes have been recognized as one type of the most fundamental synthons due to their ample availability and divergent reactivity. As a consequence, alkynes have been reported to undergo numerous useful transformations, among which the gold-catalyzed intermolecular *N*-oxide oxidation of alkynes *via* a presumable α-oxo gold carbene pathway has attracted significant research attention.^5^ This protocol renders readily available and safer alkynes as the replacement for hazardous, inaccessible, and potentially explosive α-diazo carbonyls for the generation of α-oxo gold carbenes that can react with different nucleophiles for various functionalizations. Therefore, many elegant studies have been published for synthetic applications of this chemistry over the last several years.^6,7^ However, the intermolecular oxidative reaction of alkynes with external nucleophiles is still challenging and successful examples are rather limited.^7^ There are at least two competing reactions. One is the background reaction of external nucleophiles with the activated alkynes, which produces the corresponding olefins, and another is the overoxidation of the generated electrophilic carbene center,^8^ which affords diketone byproducts.

In our recent study on ynamide chemistry,^9,10^ we found that this kind of intermolecular oxidation of alkynes could occur efficiently by employing zinc as catalyst, thus leading to the facile synthesis of α-azido, α-thiocyanate, α-halide and α-aryloxy amides.^11^ It is important to note that this oxidative zinc catalysis could significantly prohibit the overoxidation reaction. Motivated by these results, we envisioned that judicious choice of a metal catalyst would enable the construction of α-alkoxyl amides *via* such an oxidative reaction of ynamides with alcohols (Scheme 1b). We report herein the realization of the scandium-catalyzed oxidative reaction between ynamides and alcohols, delivering various α-alkoxyl amides in high yields. Additionally, this reaction, which employs readily available reagents and catalysts, proceeds under very mild conditions, and is compatible with a broad range of functional groups. In this paper, we wish to report the results of our detailed investigations of this scandium-catalyzed reaction of ynamides with alcohols and thiols, including substrate scope, synthetic applications and mechanistic studies.

## Results and discussion

Inspired by our previous work on the oxidative zinc catalysis,^11*a*^ Bs-substituted ynamide 1a and benzyl alcohol 2a were initially chosen as the model substrates for the study. As shown in Table 1, the first set of experiment using 2,6-dibromopyridine *N*-oxide 3a as the oxidant and Ph_3_PAuNTf_2_ as the catalyst afforded only the overoxidation product 4aa (Table 1, entry 1). When switching to IPrAuNTf_2_ as the catalyst, the desired α-alkoxyl amide 4a began to form as a minor product (Table 1, entry 2). Then, other metals were tested for the reaction, and it was found that Zn(OTf)_2_, Fe(OTf)_3_, Yb(OTf)_3_ and In(OTf)_3_ could produce 4a as the major product along with small amounts of hydration byproduct 4ab (Table 1, entries 3–6). To our delight, when Sm(OTf)_3_ or Sc(OTf)_3_ was employed as the catalyst (Table 1, entries 7 and 8), 4a was generated as nearly the only product and the yield could be up to 95% for the reaction using Sc(OTf)_3_ (Table 1, entry 8). In addition, Brønsted acids such as HNTf_2_ and MsOH were not effective in promoting this reaction (Table 1, entries 9 and 10). Further investigation indicated that replacing 2,6-dibromopyridine *N*-oxide 3a with other pyridine *N*-oxides, such as 2,6-dichloropyridine *N*-oxide 3b and 2-bromopyridine *N*-oxide 3c, may jeopardize the reaction, leading to poor chemoselectivity (Table 1, entries 11 and 12).

**Table 1:** Optimization of reaction conditions^a^

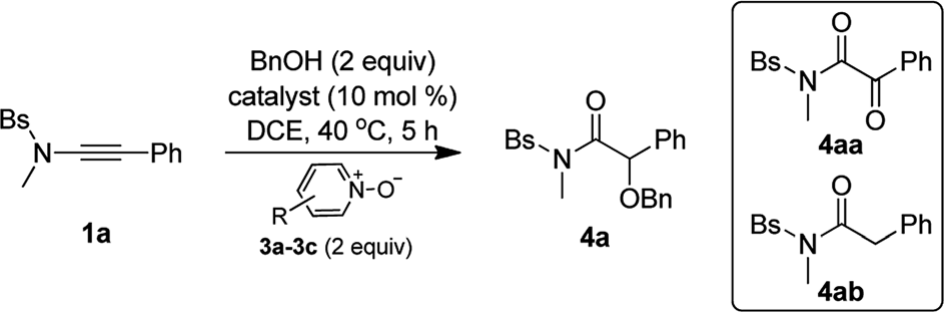					
Entry	Catalyst	Oxident (R)	Yield^b^ (%)		
			4a	4aa	4ab
1	PPh_3_AuNTf_2_	3a (2,6-Br_2_)	<1	90	<1
2	IPrAuNTf_2_	3a (2,6-Br_2_)	17	72	<1
3	Zn(OTf)_2_	3a (2,6-Br_2_)	75	<1	12
4	Fe(OTf)_3_	3a (2,6-Br_2_)	65	<1	32
5	Yb(OTf)_3_	3a (2,6-Br_2_)	69	<1	12
6	In(OTf)_3_	3a (2,6-Br_2_)	64	<1	18
7	Sm(OTf)_3_	3a (2,6-Br_2_)	74	<1	<1
**8**	**Sc(OTf)**_**3**_	3a**(2,6-Br**_**2**_**)**	**95**	**<1**	**<1**
9	HNTf_2_	3a (2,6-Br_2_)	39	<1	23
10	MsOH	3a (2,6-Br_2_)	<1	<1	70
11	Sc(OTf)_3_	3b (2,6-Cl_2_)	88	<1	6
12	Sc(OTf)_3_	3c (2-Br)	80	<1	11

a Reactions run in vials; [1a] = 0.05 M.

b Estimated by ^1^H NMR using diethyl phthalate as an internal reference.

We then used the optimal reaction conditions (Table 1, entry 8) to explore the scope of the reaction with respect to the variation of the ynamides and alcohols. As summarized in Table 2, a wide range of ynamides were applicable and good to excellent isolated yields of the α-alkoxyl amides 4a–4k were achieved (Table 2, 1–11). Ynamides bearing different alkyl or aryl groups on nitrogen atom (R^1^) and aromatic rings on the triple bond (R^2^) were all compatible with this reaction. Notably, with an alkyl-substituted (R^2^ = alkyl) substrate, the reaction only led to the formation of an α,β-unsaturated amide.^12^ Attempts to extend the reaction to the terminal ynamide only gave a complex mixture of products. Then, a significant variation of alcohols was screened, as exemplified by entries 12–20 (4l–4t). Alcohols with acyclic or cyclic alkyl groups of different carbon numbers reacted well with ynamides, leading to the desired products 4l–4o in generally excellent yields (Table 2, 12–15). A series of functional groups, such as fluoro, TMS and π-bonds, were all well tolerated under the reaction conditions, and the corresponding products 4p–4t were formed in 92–97% yields (Table 2, 16–20). Impressively, propargyl alcohol underwent this reaction smoothly with its triple bond untouched, demonstrating the chemoselectivity and preparative utility of the developed methodology (Table 2, entry 20).

**Table 2:** Reaction scope study^a^

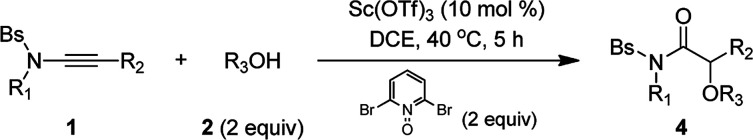
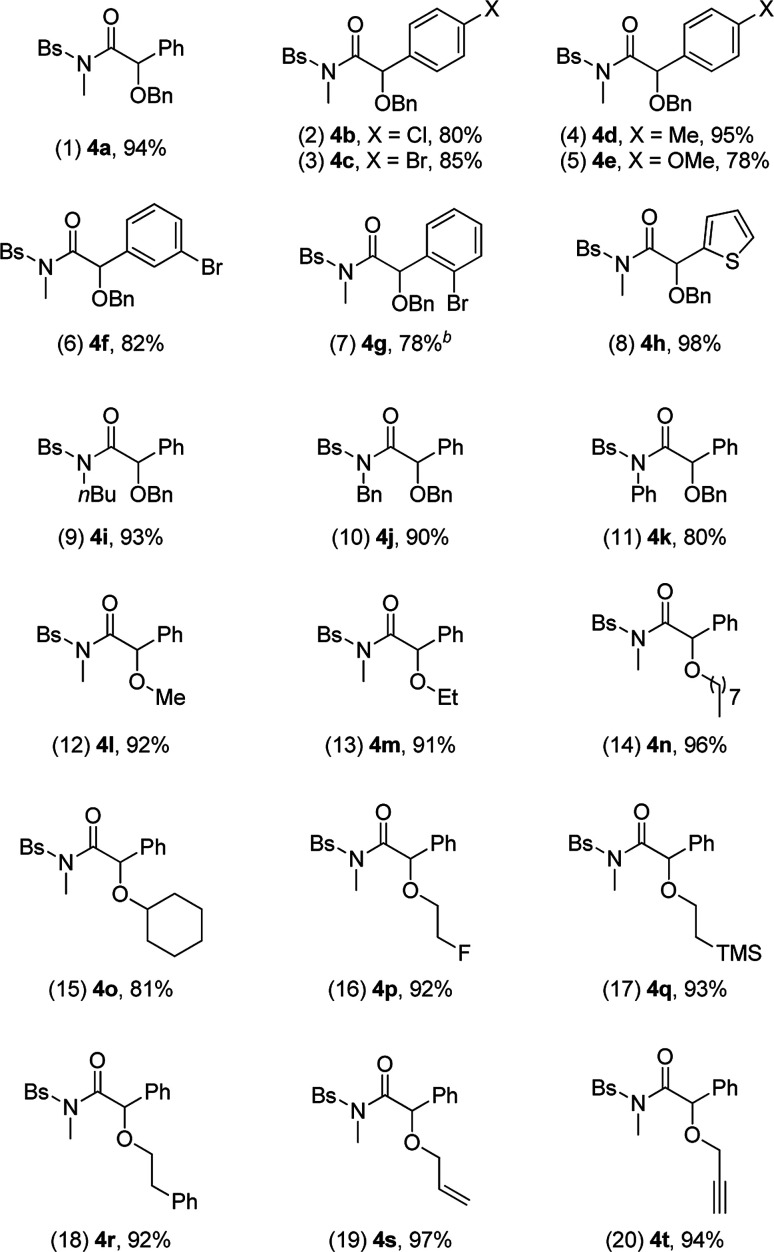

a Reactions run in vials; [1] = 0.05 M; isolated yields are reported.

b 3 equiv. of 3a was employed.

Besides alcohols, this oxidative scandium catalysis was applicable to aliphatic thiols 5, allowing the facile synthesis of the desired α-alkylthio amides 6a and 6b in 61–69% yields (eqn (1)). Of note, these sulfur-containing molecules are potentially useful in organic and medicinal chemistry.^13^ In addition, this scandium catalysis was also extended to phenol (eqn (2)), and the desired α-phenoxy amide 6c was obtained in slightly improved yield (77% *vs.* 74%) compared to the related oxidative zinc catalysis.^11*a*^1
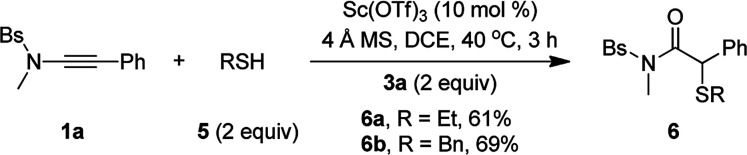
2
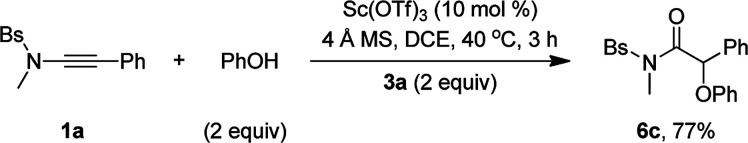


**Scheme 2:**
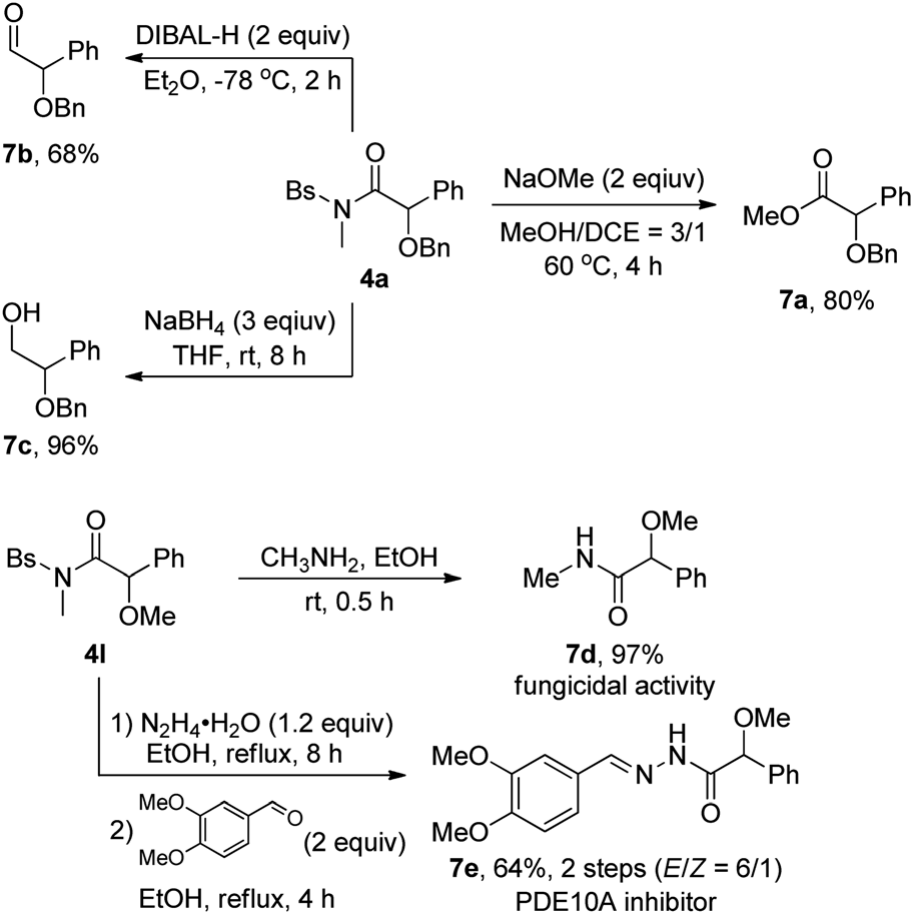
Transformation of selected α-alkoxyl amides.

The utility of this methodology was also showcased by the transformation of the as-synthesized α-alkoxyl amides 4, as outlined in Scheme 2. The amide 4a could be readily converted into the corresponding synthetically useful α-alkoxyl ester 7a in 80% yield, α-alkoxyl aldehyde 7b in 68% yield and β-alkoxyl alcohol 7c in 96% yield, respectively. Moreover, the synthesis of α-methoxyl amide 7d with fungicidal activity^1*g*^ and PDE10A inhibitor^1*a*^7e could be readily achieved by starting from the corresponding α-alkoxyl amide 4l.

**Scheme 3:**
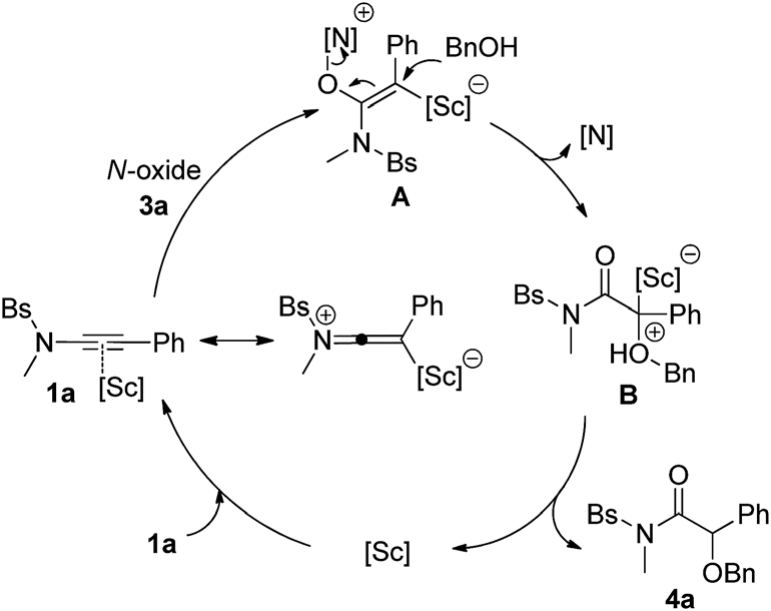
Mechanistic rationale for the synthesis of α-alkoxyl amide 4a.

Based on the obtained results and our previous reports,^11^ we propose the following mechanism with ynamide 1a and benzyl alcohol 2a as the substrates (Scheme 3). Initially, the pyridine *N*-oxide 3a attacks the Sc(iii)-activated substrate 1a to deliver the vinyl Sc(iii) intermediate A. The resulting A then undergoes an intermolecular S_N_2′ pathway and subsequent protodemetalation, leading to the final α-alkoxyl amide 4a and regenerating the scandium catalyst. In contrast, trapping of the intermediate A by another *N*-oxide leads to the formation of diketone 4aa. It is noteworthy that the activation of alkynes by scandium has relatively seldom been explored.^14^

## Conclusions

In conclusion, we have presented a novel scandium-catalyzed oxidative reaction between ynamides and alcohols, leading to the facile synthesis of valuable α-alkoxyl amides in good to excellent yields. This reaction avoids the need for the use of α-diazo carbonyls which are unstable and may cause some safety concerns. Instead, by using alkynes as the starting material, this protocol features readily available substrates, compatibility with a broad range of functional groups, a simple procedure, mild reaction conditions, and high chemoselectivity. Additional exploration on the asymmetric version of this oxidative reaction and further synthetic applications of this chemistry are currently underway in our group.

## Experimental section

### General information

Ethyl acetate (ACS grade), hexanes (ACS grade) and anhydrous 1,2-dichloroethane (ACS grade) were obtained commercially and used without further purification. Methanol, tetrahydrofuran and diethyl ether were purified according to standard methods unless otherwise noted. Commercially available reagents were used without further purification. High-resolution mass spectra were obtained using electrospray ionization using an ICR analyzer (ESI-MS). ^1^H NMR spectra were recorded in chloroform-d_3_. Chemical shifts are reported in ppm with the internal TMS signal at 0.0 ppm as a standard. The data are being reported as (s = singlet, d = doublet, t = triplet, m = multiplet or unresolved, brs = broad singlet, coupling constant(s) in Hz, integration). ^13^C NMR spectra were recorded in chloroform-d_3_. Chemical shifts are reported in ppm with the internal chloroform signal at 77.0 ppm as a standard.

Ynamides 1 were prepared according to the known procedure.^15^ The data of the ynamides 1a–1h, 1j and 1k were reported in our previous work.^11^

#### 4-Bromo-*N*-butyl-*N*-(phenylethynyl)benzenesulfonamide (1i)

Pale yellow oil (80%). ^1^H NMR (400 MHz, CDCl_3_) *δ* 7.81 (d, *J* = 8.8 Hz, 2H), 7.70 (d, *J* = 8.8 Hz, 2H), 7.42–7.36 (m, 2H), 7.35–7.20 (m, 3H), 3.41 (t, *J* = 7.2 Hz, 2H), 1.76–1.63 (m, 2H), 1.47–1.31 (m, 2H), 0.93 (t, *J* = 7.2 Hz, 3H); ^13^C NMR (100 MHz, CDCl_3_) *δ* 136.5, 132.4, 131.4, 129.0, 128.7, 128.3, 128.0, 122.5, 81.8, 70.9, 51.5, 29.9, 19.4, 13.5; IR (neat): 3054, 2930, 2235, 1358, 958, 724; HRESIMS calcd for [C_18_H_18_BrNNaO_2_S]^+^ (M + Na^+^) 414.0134, found 414.0135.

### General procedure for the scandium-catalyzed synthesis of α-alkoxyl amide 4

2,6-Dibromopyridine *N*-oxide (151.7 mg, 0.60 mmol) and Sc(OTf)_3_ (14.7 mg, 0.03 mmol) were added in this order to a mixture of the ynamide 1 (0.30 mmol) and alcohol 2 (0.60 mmol) in DCE (6.0 mL) at room temperature. The reaction mixture was stirred at 40 °C and the progress of the reaction was monitored by TLC. The reaction typically took 5 h. Upon completion, the mixture was then concentrated and the residue was purified by chromatography on silica gel (eluent: hexanes/ethyl acetate) to afford the desired α-alkoxyl amide 4.

#### 2-(Benzyloxy)-*N*-((4-bromophenyl)sulfonyl)-*N*-methyl-2-phenylacetamide (4a)

Pale yellow oil (133.8 mg, 94%). ^1^H NMR (400 MHz, CDCl_3_) *δ* 7.56 (s, 4H), 7.44–7.19 (m, 10H), 5.50 (s, 1H), 4.62–4.48 (m, 2H), 3.15 (s, 3H); ^13^C NMR (125 MHz, CDCl_3_) *δ* 170.4, 137.2, 136.7, 134.6, 132.1, 129.5, 129.0, 128.8, 128.5, 128.1, 128.0, 127.8, 80.6, 71.6, 32.9; IR (neat): 2924, 1770 (s), 1345, 1240, 1158, 743; HRESIMS calcd for [C_22_H_20_BrNNaO_4_S]^+^ (M + Na^+^) 496.0189, found 496.0195.

#### *N*-((4-Bromophenyl)sulfonyl)-*N*-methyl-2-oxo-2-phenylacetamide (4aa)

Pale yellow oil. ^1^H NMR (400 MHz, CDCl_3_) *δ* 7.94 (d, *J* = 7.2 Hz, 2H), 7.88 (d, *J* = 8.8 Hz, 2H), 7.76 (d, *J* = 8.8 Hz, 2H), 7.66 (t, *J* = 7.6 Hz, 1H), 7.54 (t, *J* = 7.6 Hz, 2H), 3.26 (s, 3H); ^13^C NMR (125 MHz, CDCl_3_) *δ* 188.0, 167.0, 135.5, 134.7, 132.9, 132.5, 130.2, 129.8, 129.7, 128.9, 30.9; IR (neat): 2924, 1770 (s), 1345, 1240, 1158, 743; HRESIMS calcd for [C_15_H_12_BrNNaO_4_S]^+^ (M + Na^+^) 403.9563, found 403.9565.

#### *N*-((4-Bromophenyl)sulfonyl)-*N*-methyl-2-oxo-2-phenylacetamide (4ab)

Pale yellow oil. ^1^H NMR (400 MHz, CDCl_3_) *δ* 7.74–7.54 (m, 4H), 7.40–7.20 (m, 3H), 7.20–7.06 (m, 2H), 3.99 (s, 2H), 3.29 (s, 3H); ^13^C NMR (125 MHz, CDCl_3_) *δ* 171.0, 137.7, 132.9, 132.4, 129.2, 129.1, 129.0, 128.7, 127.3, 43.1, 33.4; IR (neat): 2924, 1770 (s), 1345, 1240, 1158, 743; HRESIMS calcd for [C_15_H_14_BrNNaO_3_S]^+^ (M + Na^+^) 389.9770, found 389.9772.

#### 2-(Benzyloxy)-*N*-((4-bromophenyl)sulfonyl)-2-(4-chlorophenyl)-*N*-methylacetamide (4b)

Pale yellow oil (122.1 mg, 80%). ^1^H NMR (400 MHz, CDCl_3_) *δ* 7.57 (s, 4H), 7.37–7.24 (m, 9H), 5.53 (s, 1H), 4.61–4.40 (m, 2H), 3.14 (s, 3H); ^13^C NMR (100 MHz, CDCl_3_) *δ* 170.2, 137.2, 136.5, 135.1, 133.3, 132.3, 129.4, 129.2, 129.1, 129.0, 128.6, 128.3, 128.2, 79.9, 71.7, 33.0; IR (neat): 3054, 2937, 1596 (s), 1349, 1164, 1090, 744; HRESIMS calcd for [C_22_H_19_BrClNNaO_4_S]^+^ (M + Na^+^) 529.9799, found 529.9797.

#### 2-(Benzyloxy)-2-(4-bromophenyl)-*N*-((4-bromophenyl)sulfonyl)-*N*-methylacetamide (4c)

Pale yellow oil (141.1 mg, 85%). ^1^H NMR (400 MHz, CDCl_3_) *δ* 7.57 (s, 4H), 7.53–7.40 (m, 2H), 7.39–7.31 (m, 3H), 7.31–7.26 (m, 2H), 7.24–7.15 (m, 2H), 5.51 (s, 1H), 4.55–4.48 (m, 2H), 3.14 (s, 3H); ^13^C NMR (100 MHz, CDCl_3_) *δ* 170.1, 137.2, 136.5, 133.9, 132.3, 132.0, 129.5, 129.3, 129.1, 128.6, 128.2, 128.1, 123.3, 80.0, 71.7, 33.0; IR (neat): 3054, 2920, 1353, 1261, 1164, 784; HRESIMS calcd for [C_22_H_19_Br_2_NNaO_4_S]^+^ (M + H^+^) 573.9294, found 573.9296.

#### 2-(Benzyloxy)-*N*-((4-bromophenyl)sulfonyl)-*N*-methyl-2-(*p*-tolyl)acetamide (4d)

Pale yellow oil (139.2 mg, 95%). ^1^H NMR (400 MHz, CDCl_3_) *δ* 7.60–7.53 (m, 4H), 7.35–7.26 (m, 5H), 7.18–7.12 (m, 4H), 5.43 (s, 1H), 4.56–4.49 (m, 2H), 3.15 (s, 3H), 2.35 (s, 3H); ^13^C NMR (100 MHz, CDCl_3_) *δ* 170.6, 139.0, 137.4, 136.9, 132.1, 131.5, 129.5, 129.4, 128.9, 128.5, 128.1, 128.0, 127.8, 80.4, 71.5, 32.9, 21.2; IR (neat): 3022, 2926, 1614 (s), 1345, 1158, 742; HRESIMS calcd for [C_23_H_22_BrNNaO_4_S]^+^ (M + Na^+^) 510.0345, found 510.0348.

#### 2-(Benzyloxy)-*N*-((4-bromophenyl)sulfonyl)-2-(4-methoxyphenyl)-*N*-methylacetamide (4e)

Colorless oil (118.0 mg, 78%). ^1^H NMR (400 MHz, CDCl_3_) *δ* 7.59–7.51 (m, 4H), 7.37–7.28 (m, 5H), 7.21–7.16 (m, 2H), 6.88–6.83 (m, 2H), 5.43 (s, 1H), 4.51 (s, 2H), 3.81 (s, 3H), 3.14 (s, 3H); ^13^C NMR (100 MHz, CDCl_3_) *δ* 170.6, 160.2, 137.3, 136.9, 132.1, 129.5, 129.4, 128.9, 128.5, 128.2, 128.0, 126.5, 114.2, 79.8, 71.3, 55.3, 32.9; IR (neat): 3089, 2924, 1709 (s), 1512, 1361; 1250, 1173, 1068, 827, 750; HRESIMS calcd for [C_23_H_22_BrNNaO_5_S]^+^ (M + Na^+^) 526.0294, found 526.0298.

#### 2-(Benzyloxy)-2-(3-bromophenyl)-*N*-((4-bromophenyl)sulfonyl)-*N*-methylacetamide (4f)

Pale yellow oil (131.2 mg, 82%). ^1^H NMR (400 MHz, CDCl_3_) *δ* 7.59 (s, 4H), 7.51–7.46 (m, 1H), 7.40–7.28 (m, 6H), 7.25–7.19 (m, 2H), 5.50 (s, 1H), 4.59–4.49 (m, 2H), 3.16 (s, 3H); ^13^C NMR (100 MHz, CDCl_3_) *δ* 170.0, 137.2, 137.1, 136.4, 132.4, 132.1, 130.6, 130.4, 129.3, 129.2, 128.6, 128.3(0), 128.2(7), 126.4, 122.9, 80.1, 72.0, 33.0; IR (neat): 2920, 1766 (s), 1358, 1258, 1172, 764; HRESIMS calcd for [C_22_H_19_Br_2_NNaO_4_S]^+^ (M + Na^+^) 573.9294, found 573.9298.

#### 2-(Benzyloxy)-2-(2-bromophenyl)-*N*-((4-bromophenyl)sulfonyl)-*N*-methylacetamide (4g)

Pale yellow oil (124.8 mg, 78%). ^1^H NMR (500 MHz, CDCl_3_) *δ* 7.72 (d, *J* = 8.5 Hz, 2H), 7.59–7.53 (m, 3H), 7.33–7.19 (m, 8H), 5.83 (s, 1H), 4.60 (d, *J* = 11.0 Hz, 1H), 4.54 (d, *J* = 11.0 Hz, 1H), 3.16 (s, 3H); ^13^C NMR (125 MHz, CDCl_3_) *δ* 169.2, 137.2, 136.4, 134.4, 133.0, 132.1, 130.5, 129.6, 129.2, 128.9, 128.4, 128.1, 127.9, 124.5, 79.3, 72.4, 32.8; IR (neat): 2921, 1768 (s), 1356, 1241, 1172, 764; HRESIMS calcd for [C_22_H_19_Br_2_NNaO_4_S]^+^ (M + Na^+^) 573.9294, found 573.9294.

#### 2-(Benzyloxy)-*N*-((4-bromophenyl)sulfonyl)-*N*-methyl-2-(thiophen-2-yl)acetamide (4h)

Colorless oil (141.2 mg, 98%). ^1^H NMR (400 MHz, CDCl_3_) *δ* 7.59–7.53 (m, 4H), 7.39–7.29 (m, 6H), 6.99–6.96 (m, 2H), 5.85 (s, 1H), 4.55 (dd, *J* = 17.2, 11.6 Hz, 2H), 3.18 (s, 3H); ^13^C NMR (100 MHz, CDCl_3_) *δ* 169.6, 137.3, 137.1, 136.5, 132.3, 129.3, 129.1, 128.5, 128.3, 128.2, 127.9, 127.6, 126.8, 75.7, 71.3, 33.1; IR (neat): 3089, 3031, 2922, 1707 (s), 1573, 1390, 1361, 1170, 1068, 742; HRESIMS calcd for [C_20_H_18_BrNNaO_4_S_2_]^+^ (M + Na^+^) 501.9753, found 501.9756.

#### 2-(Benzyloxy)-*N*-((4-bromophenyl)sulfonyl)-*N*-butyl-2-phenylacetamide (4i)

Pale yellow oil (144.1 mg, 93%). ^1^H NMR (400 MHz, CDCl_3_) *δ* 7.62–7.53 (m, 4H), 7.42–7.24 (m, 10H), 5.40 (s, 1H), 4.57–4.49 (m, 2H), 3.58–3.50 (m, 2H), 1.50–1.35 (m, 1H), 1.34–1.22 (m, 1H), 1.21–1.08 (m, 2H), 0.77 (t, *J* = 7.2 Hz, 3H); ^13^C NMR (125 MHz, CDCl_3_) *δ* 170.2, 137.9, 136.8, 134.7, 132.1, 129.7, 129.1, 128.9, 128.8, 128.5, 128.2, 128.1, 127.9, 80.1, 71.5, 46.4, 31.8, 19.8, 13.4; IR (neat): 2929, 1641 (s), 1347, 1193, 1158, 742; HRESIMS calcd for [C_25_H_26_BrNNaO_4_S]^+^ (M + Na^+^) 538.0658, found 538.0658.

#### *N*-Benzyl-2-(benzyloxy)-*N*-((4-bromophenyl)sulfonyl)-2-phenylacetamide (4j)

Pale yellow oil (148.6 mg, 90%). ^1^H NMR (400 MHz, CDCl_3_) *δ* 7.53 (s, 4H), 7.36–7.24 (m, 10H), 7.18–7.05 (m, 5H), 5.25 (s, 1H), 4.90 (dd, *J* = 48.5, 15.8 Hz, 2H), 4.37 (dd, *J* = 34.0, 12.0 Hz, 2H); ^13^C NMR (125 MHz, CDCl_3_) *δ* 170.7, 137.6, 136.6, 135.7, 134.3, 132.0, 130.0, 129.3, 129.0, 128.9, 128.8, 128.4, 128.2, 128.1, 128.0, 127.9, 126.9, 79.7, 71.3, 49.2; IR (neat): 2927, 1607 (s), 1435, 1246, 1158, 947; HRESIMS calcd for [C_28_H_24_BrNNaO_4_S]^+^ (M + Na^+^) 572.0502, found 572.0504.

#### 2-(Benzyloxy)-*N*-((4-bromophenyl)sulfonyl)-*N*,2-diphenylacetamide (4k)

Pale yellow oil (128.7 mg, 80%). ^1^H NMR (400 MHz, CDCl_3_) ^1^H NMR (400 MHz, CDCl_3_) *δ* 7.87 (d, *J* = 6.8 Hz, 2H), 7.69 (d, *J* = 6.8 Hz, 2H), 7.41 (t, *J* = 7.6 Hz, 1H), 7.33–7.20 (m, 8H), 7.19–7.09 (m, 2H), 6.94–6.88 (m, 2H), 6.79 (d, *J* = 7.2 Hz, 2H), 4.58 (s, 1H), 4.35 (dd, *J* = 40.4, 12.0 Hz, 2H); ^13^C NMR (125 MHz, CDCl_3_) *δ* 169.8, 137.5, 136.7, 134.2, 132.0, 130.8, 130.5, 130.2, 129.4, 129.3, 129.2, 128.6, 128.5, 128.4, 128.1, 128.0, 126.9, 78.8, 70.8; IR (neat): 2927, 1607 (s), 1435, 1246, 1158, 947; HRESIMS calcd for [C_27_H_22_BrNNaO_4_S]^+^ (M + Na^+^) 558.0345, found 558.0349.

#### *N*-((4-Bromophenyl)sulfonyl)-2-methoxy-*N*-methyl-2-phenylacetamide (4l)

Pale yellow oil (109.9 mg, 92%). ^1^H NMR (500 MHz, CDCl_3_) *δ* 7.61–7.55 (m, 4H), 7.38–7.33 (m, 3H), 7.30–7.26 (m, 2H), 5.33 (s, 1H), 3.38 (s, 3H), 3.18 (s, 3H); ^13^C NMR (125 MHz, CDCl_3_) *δ* 170.5, 137.3, 134.4, 132.2, 129.5, 129.1, 129.0, 128.9, 127.8, 83.4, 57.6, 32.9; IR (neat): 3022, 2926, 1614 (s), 1345, 1158, 742; HRESIMS calcd for [C_16_H_16_BrNNaO_4_S]^+^ (M + Na^+^) 419.9876, found 419.9875.

#### *N*-((4-Bromophenyl)sulfonyl)-2-ethoxy-*N*-methyl-2-phenylacetamide (4m)

Pale yellow oil (112.6 mg, 91%). ^1^H NMR (500 MHz, CDCl_3_) *δ* 7.60 (s, 4H), 7.36–7.31 (m, 3H), 7.28–7.24 (m, 2H), 5.39 (s, 1H), 3.60–3.50 (m, 2H), 3.20 (s, 3H), 1.24 (t, *J* = 7.2 Hz, 3H); ^13^C NMR (125 MHz, CDCl_3_) *δ* 170.8, 137.3, 134.9, 132.2, 129.6, 129.0, 128.9, 128.8, 127.5, 82.2, 65.8, 32.9, 15.1; IR (neat): 3055, 2930, 1603 (s), 1347, 1159, 949; HRESIMS calcd for [C_17_H_18_BrNNaO_4_S]^+^ (M + H^+^) 434.0032, found 434.0030.

#### *N*-((4-Bromophenyl)sulfonyl)-2-ethoxy-*N*-methyl-2-phenylacetamide (4n)

Pale yellow oil (143.0 mg, 96%). ^1^H NMR (400 MHz, CDCl_3_) *δ* 7.64–7.58 (m, 4H), 7.35–7.30 (m, 3H), 7.27–7.24 (m, 2H), 5.35 (s, 1H), 3.52–3.41 (m, 2H), 3.20 (s, 3H), 1.62–1.55 (m, 2H), 1.47–1.20 (m, 10H), 0.88 (t, *J* = 6.4 Hz, 3H); ^13^C NMR (100 MHz, CDCl_3_) *δ* 170.8, 137.5, 135.0, 132.1, 129.6, 128.9, 128.8, 127.2, 82.5, 70.6, 32.8, 31.8, 29.7, 29.3, 29.2, 26.0, 22.6, 14.0; IR (neat): 2924, 1347, 1193, 1158, 743; HRESIMS calcd for [C_23_H_30_BrNNaO_4_S]^+^ (M + Na^+^) 518.0971, found 518.0970.

#### *N*-((4-Bromophenyl)sulfonyl)-2-(cyclohexyloxy)-*N*-methyl-2-phenylacetamide (4o)

Pale yellow oil (113.3 mg, 81%). ^1^H NMR (500 MHz, CDCl_3_) *δ* 7.66–7.60 (m, 4H), 7.33–7.21 (m, 5H), 5.44 (s, 1H), 3.40–3.32 (m, 1H), 3.22 (s, 3H), 1.91–1.84 (m, 2H), 1.75–1.63 (m, 2H), 1.45–1.31 (m, 2H), 1.29–1.19 (m, 4H); ^13^C NMR (125 MHz, CDCl_3_) *δ* 171.5, 137.3, 135.4, 132.0, 129.7, 128.9, 128.8, 128.5, 126.5, 80.5, 78.1, 32.9, 32.4, 25.5, 23.9; IR (neat): 2931, 1614 (s), 1346, 1158, 743; HRESIMS calcd for [C_21_H_24_BrNNaO_4_S]^+^ (M + Na^+^) 488.0502, found 409.0748.

#### *N*-((4-Bromophenyl)sulfonyl)-2-(2-fluoroethoxy)-*N*-methyl-2-phenylacetamide (4p)

Pale yellow oil (118.8 mg, 92%). ^1^H NMR (500 MHz, CDCl_3_) *δ* 7.61 (s, 4H), 7.40–7.33 (m, 3H), 7.32–7.27 (m, 2H), 5.56 (s, 1H), 4.65–4.49 (m, 2H), 3.81–3.68 (m, 2H), 3.19 (s, 3H); ^13^C NMR (125 MHz, CDCl_3_) *δ* 170.3, 137.1, 134.2, 132.2, 129.6, 129.2, 129.1, 129.0, 127.9, 83.6, 82.2, 69.0 (d, *J* = 19.5 Hz), 32.9; IR (neat): 2932, 1488, 1345, 1193, 1132, 783; HRESIMS calcd for [C_17_H_17_BrFNNaO_4_S]^+^ (M + Na^+^) 451.9938, found 451.9939.

#### *N*-((4-Bromophenyl)sulfonyl)-*N*-methyl-2-phenyl-2-(2-(trimethylsilyl)ethoxy)acetamide (4q)

Pale yellow oil (135.2 mg, 93%). ^1^H NMR (400 MHz, CDCl_3_) *δ* 7.61 (s, 4H), 7.40–7.31 (m, 3H), 7.30–7.25 (m, 2H), 5.39 (s, 1H), 3.65–3.48 (m, 2H), 3.22 (s, 3H), 1.05–0.96 (m, 2H), 0.00 (s, 9H); ^13^C NMR (125 MHz, CDCl_3_) *δ* 170.9, 137.4, 134.9, 132.1, 129.6, 129.0, 128.8, 128.7, 127.3, 82.0, 67.9, 32.9, 18.3, −1.5; IR (neat): 2926, 2855, 1694 (s), 1682, 1450, 1330, 1193, 1151, 784; HRESIMS calcd for [C_20_H_26_BrNNaO_4_SSi]^+^ (M + Na^+^) 506.0427, found 506.0429.

#### *N*-((4-Bromophenyl)sulfonyl)-*N*-methyl-2-phenethoxy-2-phenylacetamide (4r)

Pale yellow oil (134.8 mg, 92%). ^1^H NMR (500 MHz, CDCl_3_) *δ* 7.58 (s, 4H), 7.34–7.27 (m, 5H), 7.25–7.16 (m, 5H), 5.33 (s, 1H), 3.72–3.66 (m, 2H), 3.05 (s, 3H), 3.00–2.85 (m, 2H); ^13^C NMR (125 MHz, CDCl_3_) *δ* 170.6, 138.4, 137.4, 134.6, 132.1, 129.6, 129.0, 128.9, 128.9, 128.8, 128.4, 127.2, 126.4, 82.6, 71.2, 36.2, 32.7; IR (neat): 2930, 1732 (s), 1343, 1244, 1157, 745; HRESIMS calcd for [C_23_H_22_BrNNaO_4_S]^+^ (M + Na^+^) 510.0345, found 510.0348.

#### 2-(Allyloxy)-*N*-((4-bromophenyl)sulfonyl)-*N*-methyl-2-phenylacetamide (4s)

Pale yellow oil (123.5 mg, 97%). ^1^H NMR (500 MHz, CDCl_3_) *δ* 7.59 (s, 4H), 7.38–7.30 (m, 3H), 7.29–7.24 (m, 2H), 5.94–5.85 (m, 1H), 5.47 (s, 1H), 5.35–5.22 (m, 2H), 4.10–3.97 (m, 2H), 3.19 (s, 3H); ^13^C NMR (125 MHz, CDCl_3_) *δ* 170.5, 137.2, 134.5, 133.5, 132.2, 129.6, 129.1, 129.0, 128.9, 127.7, 118.5, 80.9, 70.8, 32.9; IR (neat): 2920, 1731 (s), 1346, 1244, 1159, 1014, 739; HRESIMS calcd for [C_18_H_18_BrNNaO_4_S]^+^ (M + Na^+^) 446.0032, found 446.0039.

#### *N*-((4-Bromophenyl)sulfonyl)-*N*-methyl-2-phenyl-2-(prop-2-yn-1-yloxy)acetamide (4t)

Pale yellow oil (119.1 mg, 94%). ^1^H NMR (400 MHz, CDCl_3_) *δ* 7.60 (s, 4H), 7.40–7.33 (m, 3H), 7.32–7.28 (m, 2H), 5.78 (s, 1H), 4.28–4.08 (m, 2H), 3.21 (s, 3H), 2.51 (t, *J* = 2.4 Hz, 1H); ^13^C NMR (125 MHz, CDCl_3_) *δ* 169.9, 137.1, 133.7, 132.2, 129.6, 129.4, 129.1, 129.0, 128.4, 79.2, 78.4, 76.1, 56.5, 33.0; IR (neat): 2934, 1620 (s), 1467, 1157, 746; HRESIMS calcd for [C_18_H_16_BrNNaO_4_S]^+^ (M + Na^+^) 443.9876, found 389.1062.

### General procedure for the scandium-catalyzed synthesis of α-alkylthio amide 6

2,6-Dibromopyridine *N*-oxide (151.7 mg, 0.60 mmol) and Sc(OTf)_3_ (14.7 mg, 0.03 mmol) were added in this order to a mixture of the ynamide 1 (0.30 mmol) and thiol 5 or phenol (0.60 mmol) in DCE (6.0 mL) at room temperature. The reaction mixture was stirred at 40 °C and the progress of the reaction was monitored by TLC. The reaction typically took 3 h. Upon completion, the mixture was then concentrated and the residue was purified by chromatography on silica gel (eluent: hexanes/ethyl acetate) to afford the desired α- alkylthio amide 6.

#### *N*-((4-Bromophenyl)sulfonyl)-2-(ethylthio)-*N*-methyl-2-phenylacetamide (6a)

Colorless oil (75.8 mg, 61%). ^1^H NMR (400 MHz, CDCl_3_) *δ* 7.65–7.54 (m, 4H), 7.37–7.28 (m, 5H), 5.37 (s, 1H), 3.25 (s, 3H), 2.52–2.36 (m, 2H), 1.16 (t, *J* = 7.6 Hz, 3H); ^13^C NMR (100 MHz, CDCl_3_) *δ* 170.3, 137.4, 135.3, 132.3, 129.3, 129.0, 128.8, 128.6, 128.2, 52.4, 33.5, 25.5, 14.0; IR (neat): 2920, 2849, 2359, 2341, 1694 (s), 1573, 1359, 1171, 1069, 745; HRESIMS calcd for [C_17_H_18_BrNNaO_3_S_2_]^+^ (M + Na^+^) 449.9804, found 449.9807.

#### 2-(Benzylthio)-*N*-((4-bromophenyl)sulfonyl)-*N*-methyl-2-phenylacetamide (6b)

Pale yellow oil (88.7 mg, 69%). ^1^H NMR (400 MHz, CDCl_3_) *δ* 7.59–7.51 (m, 4H), 7.36–7.21 (m, 10H), 5.03 (s, 1H), 3.62 (dd, *J* = 66.8, 13.2 Hz, 2H), 3.06 (s, 3H); ^13^C NMR (100 MHz, CDCl_3_) *δ* 169.8, 137.4, 137.1, 134.7, 132.3, 129.4, 129.1, 129.0, 128.9, 128.9, 128.7, 128.4, 127.4, 51.7, 36.0, 33.2; IR (neat): 2919, 2849, 1699 (s), 1574, 1454, 1360, 1170, 1069, 745, 699; HRESIMS calcd for [C_22_H_20_BrNNaO_3_S_2_]^+^ (M + Na^+^) 511.9960, found 511.9963.

#### *N*-((4-Bromophenyl)sulfonyl)-*N*-methyl-2-phenoxy-2-phenylacetamide (6c)

Pale yellow oil (106.3 mg, 77%). This compound is known and the spectroscopic data match those reported in our previous work.^11*a*^^1^H NMR (400 MHz, CDCl_3_) *δ* 7.52 (d, *J* = 8.4 Hz, 2H), 7.48–7.37 (m, 7H), 7.23 (d, *J* = 7.6 Hz, 2H), 7.03–6.97 (m, 1H), 6.87 (d, *J* = 8.4 Hz, 2H), 6.32 (s, 1H), 3.24 (s, 3H).

#### Methyl 2-(benzyloxy)-2-phenylacetate (7a)

Compound 7a was prepared in 80% yield according to the known procedure.^11*a*^ Colorless oil. ^1^H NMR (400 MHz, CDCl_3_) *δ* 7.48–7.29 (m, 10H), 4.94 (s, 1H), 4.58 (dd, *J* = 15.2, 11.6 Hz, 2H), 3.70 (s, 3H); ^13^C NMR (125 MHz, CDCl_3_) *δ* 171.2, 137.1, 136.2, 128.7, 128.6, 128.4, 128.0, 127.9, 127.4, 79.6, 71.1, 52.2; IR (neat): 3030, 2921, 1749 (s), 1454, 1259, 1209, 1172, 1100, 734, 697; HRESIMS calcd for [C_16_H_16_NaO_3_]^+^ (M + Na^+^) 279.0992, found 279.0995.

#### 2-(Benzyloxy)-2-phenylacetaldehyde (7b)

Compound 7b was prepared in 68% yield according to the known procedure.^16^ This compound is known and the spectroscopic data match those reported.^17^^1^H NMR (400 MHz, CDCl_3_) *δ* 9.62 (d, *J* = 1.6 Hz, 1H), 7.42–7.31 (m, 10H), 4.80 (d, *J* = 1.6 Hz, 1H), 4.67 (d, *J* = 12.0 Hz, 1H), 4.54 (d, *J* = 12.0 Hz, 1H); ^13^C NMR (100 MHz, CDCl_3_) *δ* 198.3, 137.0, 134.0, 129.0, 128.9, 128.5, 128.0(4), 128.0(0), 127.5, 85.5, 71.1.

#### 2-(Benzyloxy)-2-phenylethan-1-ol (7c)

Compound 7c was prepared in 96% yield according to the known procedure.^11*a*^ Colorless oil. ^1^H NMR (500 MHz, CDCl_3_) *δ* 7.41–7.28 (m, 10H), 4.57–4.52 (m, 2H), 4.34 (d, *J* = 11.5 Hz, 1H), 3.76–3.71 (m, 1H), 3.69–3.57 (m, 1H), 2.32 (s, 1H); ^13^C NMR (100 MHz, CDCl_3_) *δ* 138.4, 137.9, 128.6, 128.4, 128.2, 127.9, 127.8, 127.0, 82.3, 70.7, 67.4; IR (neat): 3356 (br), 2920, 2851, 1492, 1453, 1010, 1065, 1026, 736, 699; HRESIMS calcd for [C_15_H_16_NaO_2_]^+^ (M + Na^+^) 251.1043, found 251.1047.

#### 2-Methoxy-*N*-methyl-2-phenylacetamide (7d)

Compound 7d was prepared according to the known procedures.^1*a*^ This compound is known and the spectroscopic data match those reported.^1*g*,18^^1^H NMR (400 MHz, CDCl_3_) *δ* 7.42–7.28 (m, 5H), 6.79 (s, 1H), 4.61 (s, 1H), 3.35 (s, 3H), 2.82 (d, *J* = 4.8 Hz, 3H); ^13^C NMR (100 MHz, CDCl_3_) *δ* 171.1, 137.0, 128.4, 128.3, 126.9, 83.8, 57.1, 25.6.

#### (*E*)-*N*'-(3,4-Dimethoxybenzylidene)-2-methoxy-2-phenylacetohydrazide (7e)

Compound 7e was prepared according to the known procedures.^1*a*^ This compound is known and the spectroscopic data match those reported.^1*a*^^1^H NMR (400 MHz, CDCl_3_) *δ* 9.58 (s, 1H), 8.18 (s, 1H), 7.50–7.44 (m, 3H), 7.40–7.25 (m, 3H), 7.10–7.06 (m, 1H), 6.85 (d, *J* = 8.4 Hz, 1H), 4.80 (s, 1H), 3.92 (s, 3H), 3.91 (s, 3H), 3.44 (s, 3H); ^13^C NMR (100 MHz, CDCl_3_) *δ* 166.5, 151.4, 149.4, 136.3, 128.6, 128.5, 128.0, 127.1, 126.5, 122.8, 110.5, 108.4, 83.6, 57.4, 56.0, 55.9.

## Conflicts of interest

There are no conflicts to declare.

## Supplementary Material

RA-008-C8RA03842B-s001
