# Supplementary material for: Facile synthesis of α-alkoxyl amides via scandium-catalyzed oxidative reaction between ynamides and alcohols
Source: RSC Adv. 2018 May 18;8(33):18308–15. doi: 10.1039/c8ra03842b (PMC9080523; doi:10.1039/c8ra03842b)
Supplement: RA-008-C8RA03842B-s001 [file RA-008-C8RA03842B-s001.pdf]

Electronic Supplementary Material (ESI) for RSC Advances.
This journal is © The Royal Society of Chemistry 2018

Supporting Information

Facile synthesis of α-alkoxyl amides via scandium-catalyzed oxidative reaction between ynamides and alcohols

Zhi-Xin Zhang, Bo-Han Zhu, Pei-Xi Xie, Jia-Qi Tang, Xin-Ling Li, Chunyin Zhu,*
Ying-Wu Yin,* and Long-Wu Ye*

State Key Laboratory of Physical Chemistry of Solid Surfaces and Key Laboratory for Chemical Biology of Fujian Province, College of Chemistry and Chemical Engineering, Xiamen University, Xiamen 361005, China

Content	Page number
**General**	2
**^{1}H and ^{13}C NMR Spectra**	3

General Information. Ethyl acetate (ACS grade), hexanes (ACS grade) and anhydrous 1, 2-dichloroethane (ACS grade) were obtained commercially and used without further purification. Methylene chloride, tetrahydrofuran and diethyl ether were purified according to standard methods unless otherwise noted. Commercially available reagents were used without further purification. Reactions were monitored by thin layer chromatography (TLC) using silicycle pre-coated silica gel plates. Flash column chromatography was performed over silica gel (300-400 mesh). Infrared spectra were recorded on a Nicolet AVATER FTIR330 spectrometer as thin film and are reported in reciprocal centimeter (cm^{-1}). Mass spectra were recorded with Micromass QTOF2 Quadrupole/Time-of-Flight Tandem mass spectrometer using electron spray ionization.

^{1}H NMR spectra and ^{13}C NMR spectra were recorded on a Bruker AV-400 spectrometer and a Bruker AV-500 spectrometer in chloroform-d_3. For ^{1}H NMR spectra, chemical shifts are reported in ppm with the internal TMS signal at 0.0 ppm as a standard. For ^{13}C NMR spectra, chemical shifts are reported in ppm with the internal chloroform signal at 77.0 ppm as a standard.

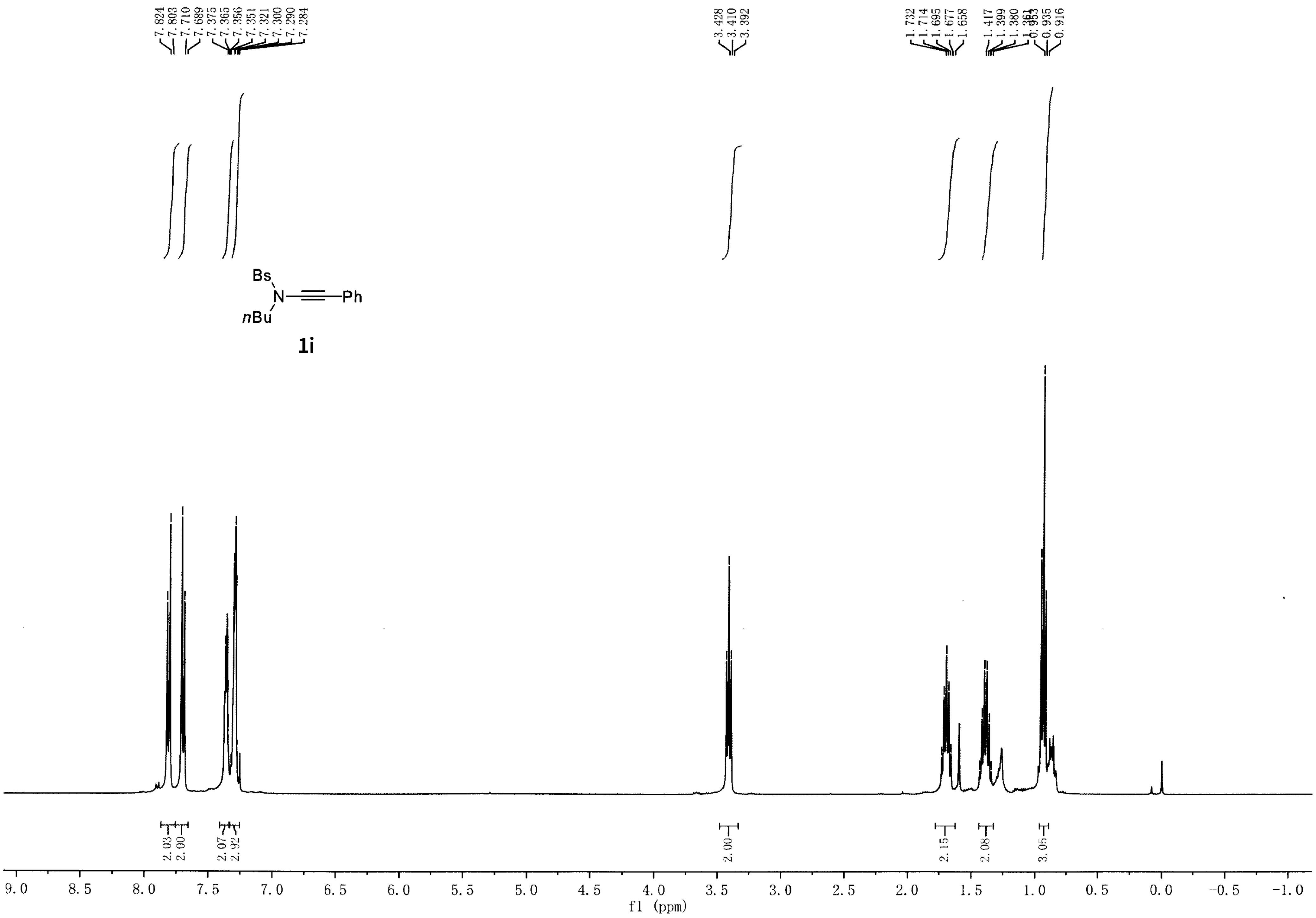

7.824
7.803
7.710
7.689
7.375
7.365
7.356
7.351
7.321
7.300
7.290
7.284
3.428
3.410
3.392
1.732
1.714
1.695
1.677
1.658
1.417
1.399
1.380
1.361
0.953
0.935
0.916
Bs
N
Ph
nBu
1i
2.03
2.00
2.07
2.92
2.00
2.15
2.08
3.05
9.0 8.5 8.0 7.5 7.0 6.5 6.0 5.5 5.0 4.5 4.0 3.5 3.0 2.5 2.0 1.5 1.0 0.5 0.0 -0.5 -1.0
f1 (ppm)

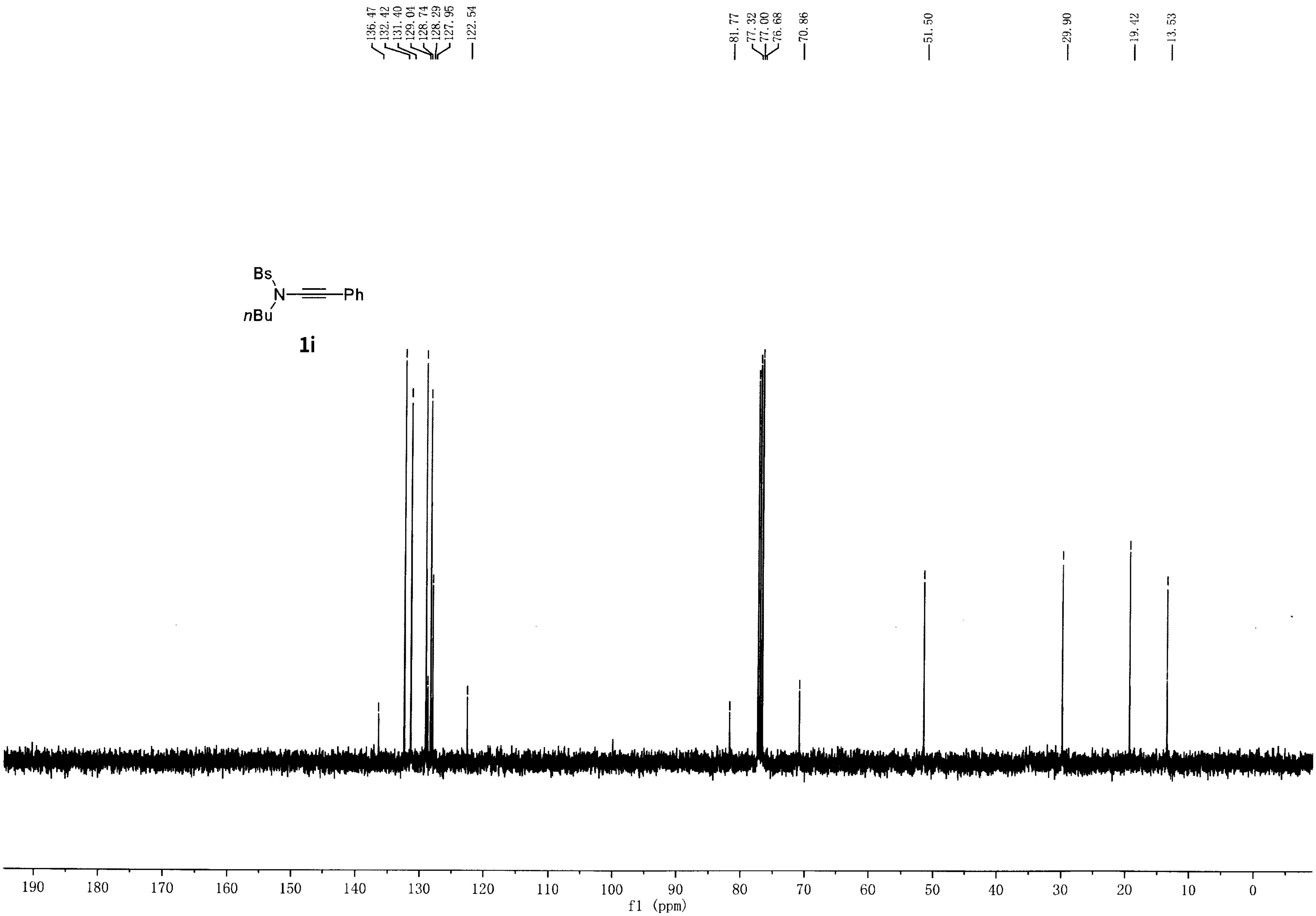
136.47
132.42
131.40
129.04
128.74
128.29
127.95
122.54
81.77
77.32
77.00
76.68
70.86
51.50
29.90
19.42
13.53
Bs
N
Ph
nBu
1i
190 180 170 160 150 140 130 120 110 100 90 80 70 60 50 40 30 20 10 0
f1 (ppm)

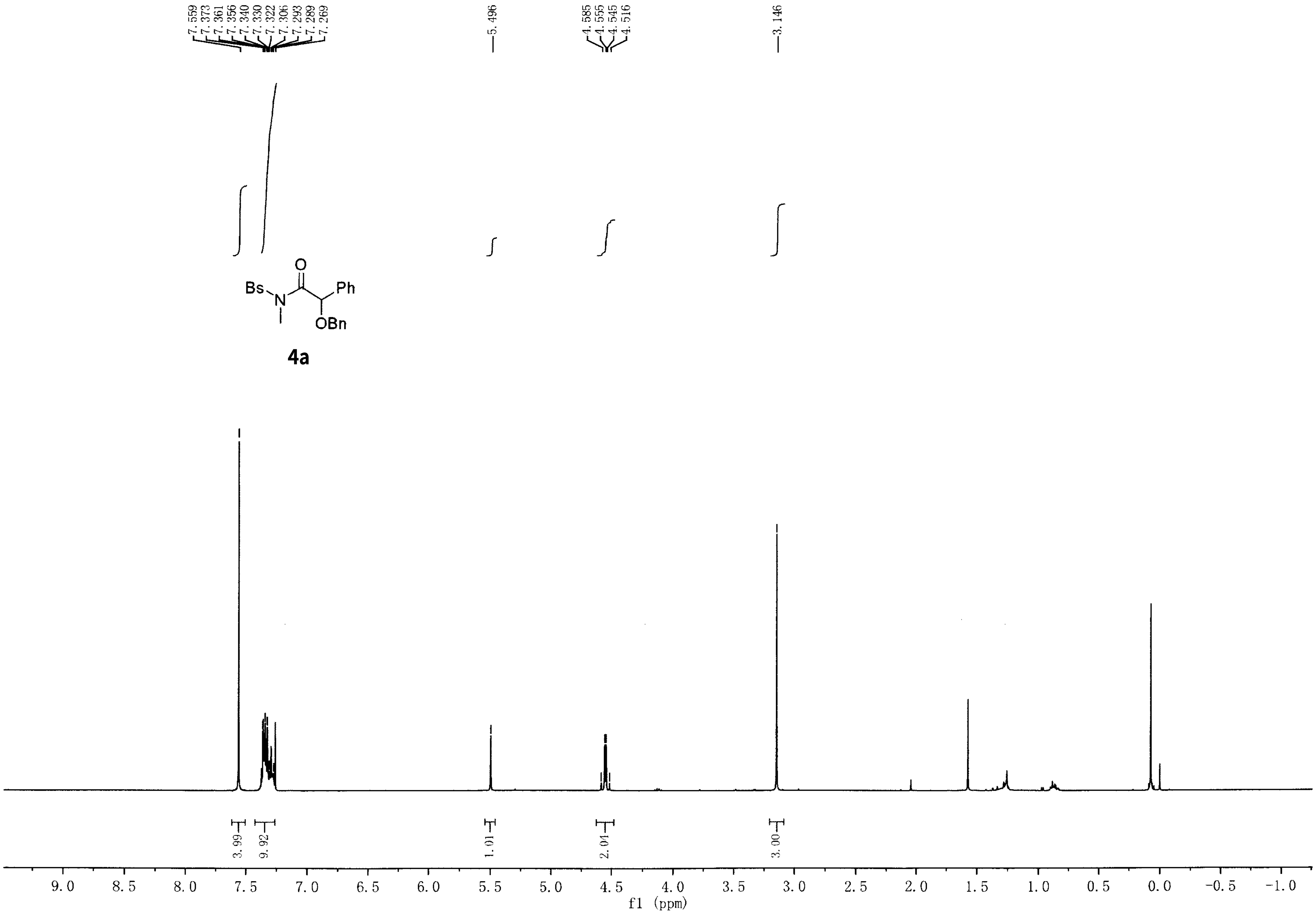

7.559
7.373
7.361
7.356
7.340
7.330
7.322
7.306
7.293
7.289
7.269
5.496
4.585
4.555
4.545
4.516
3.146
Bs
N
O
Ph
OBn
4a
3.99
9.92
1.01
2.04
3.00
9.0 8.5 8.0 7.5 7.0 6.5 6.0 5.5 5.0 4.5 4.0 3.5 3.0 2.5 2.0 1.5 1.0 0.5 0.0 -0.5 -1.0
f1 (ppm)

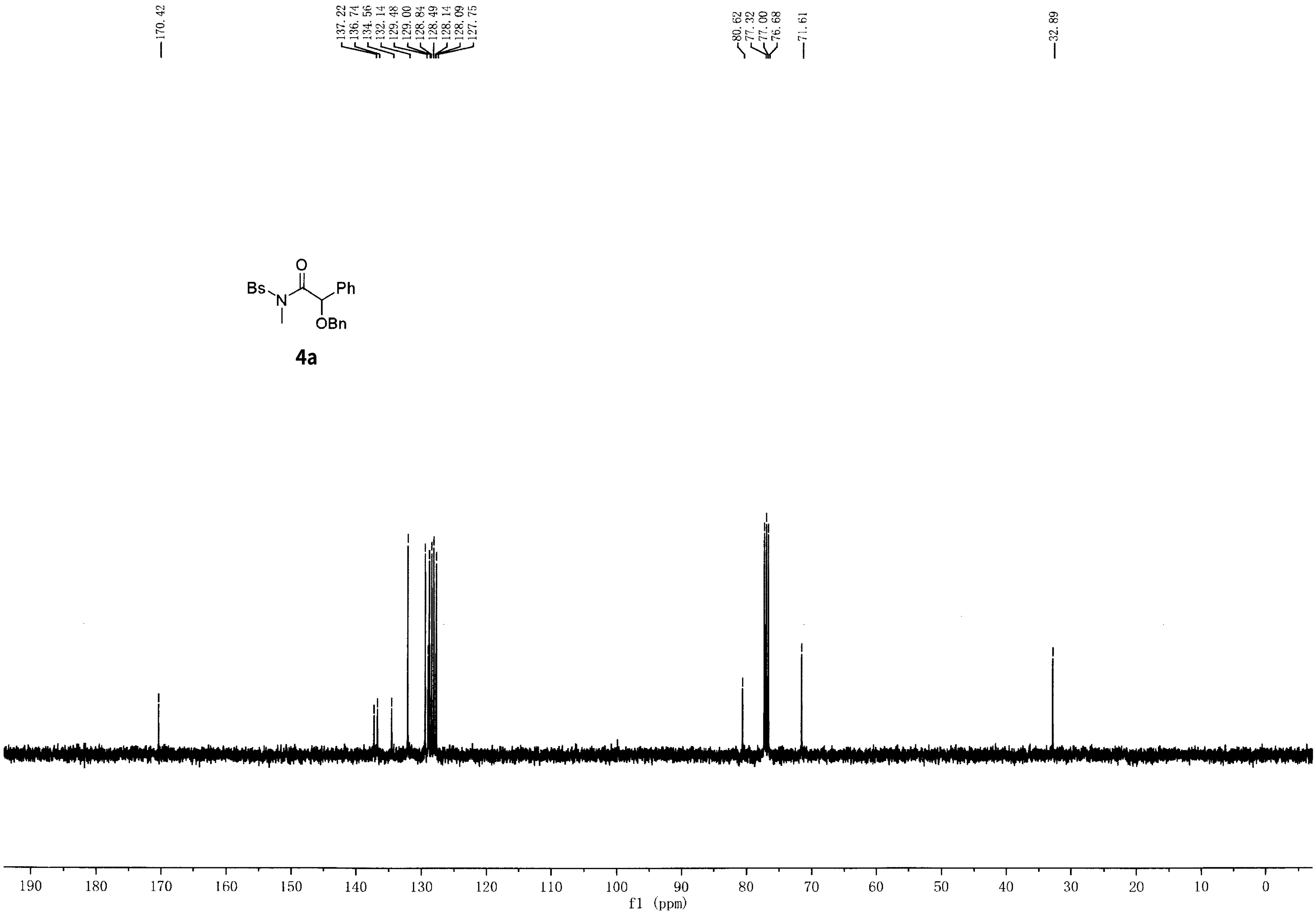
170.42
137.22
136.74
134.56
132.14
129.48
129.00
128.84
128.49
128.14
128.09
127.75
80.62
77.32
77.00
76.68
71.61
32.89
O
Bs
N
Ph
OBn
4a
190
180
170
160
150
140
130
120
110
100
90
80
70
60
50
40
30
20
10
0
f1 (ppm)

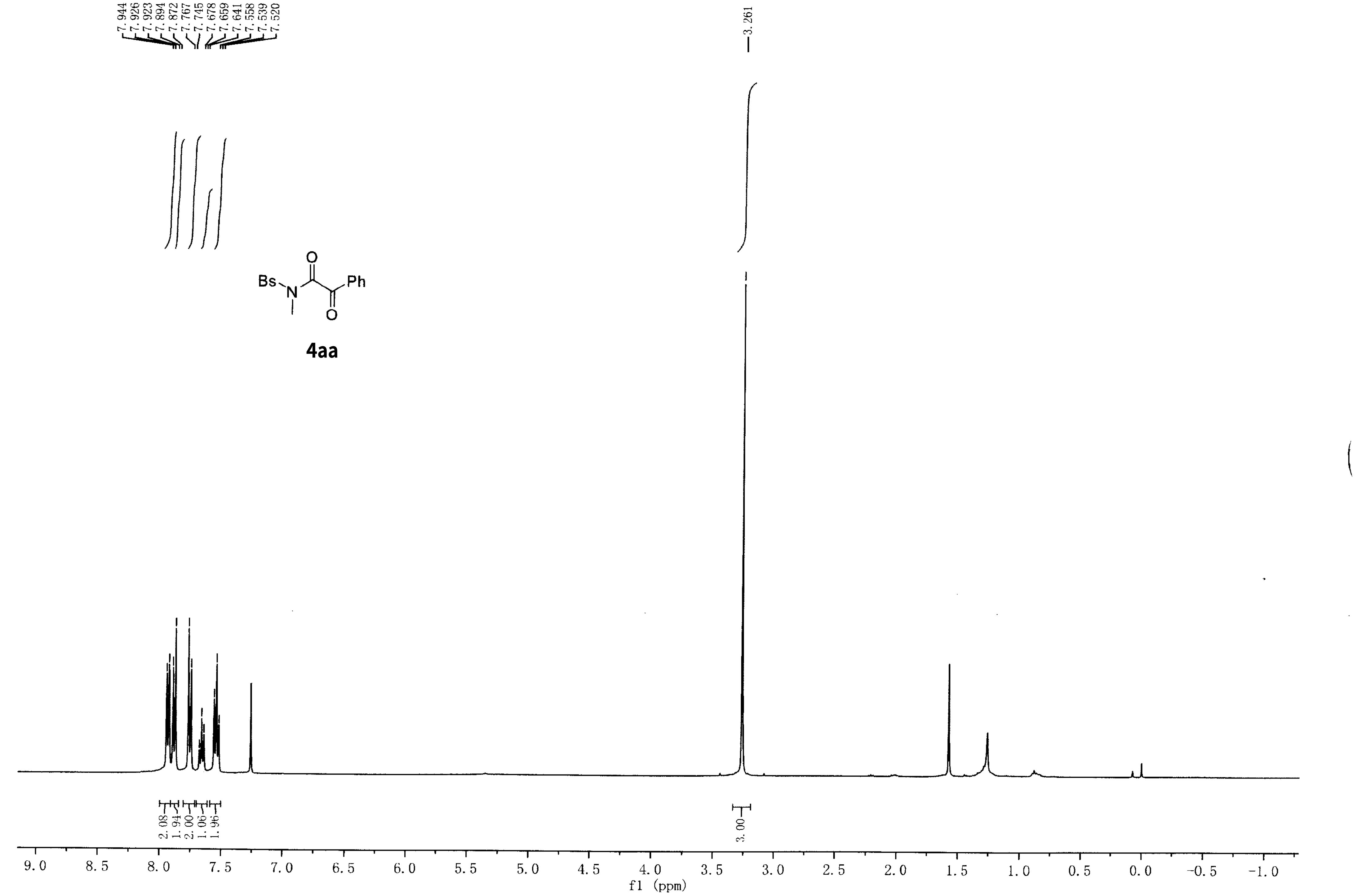
7.944
7.926
7.923
7.894
7.872
7.767
7.745
7.678
7.659
7.641
7.558
7.539
7.520
3.261
Bs
N
O
O
Ph
4aa
2.08
1.94
2.00
1.06
1.96
3.00
9.0
8.5
8.0
7.5
7.0
6.5
6.0
5.5
5.0
4.5
4.0
3.5
3.0
2.5
2.0
1.5
1.0
0.5
0.0
-0.5
-1.0
f1 (ppm)

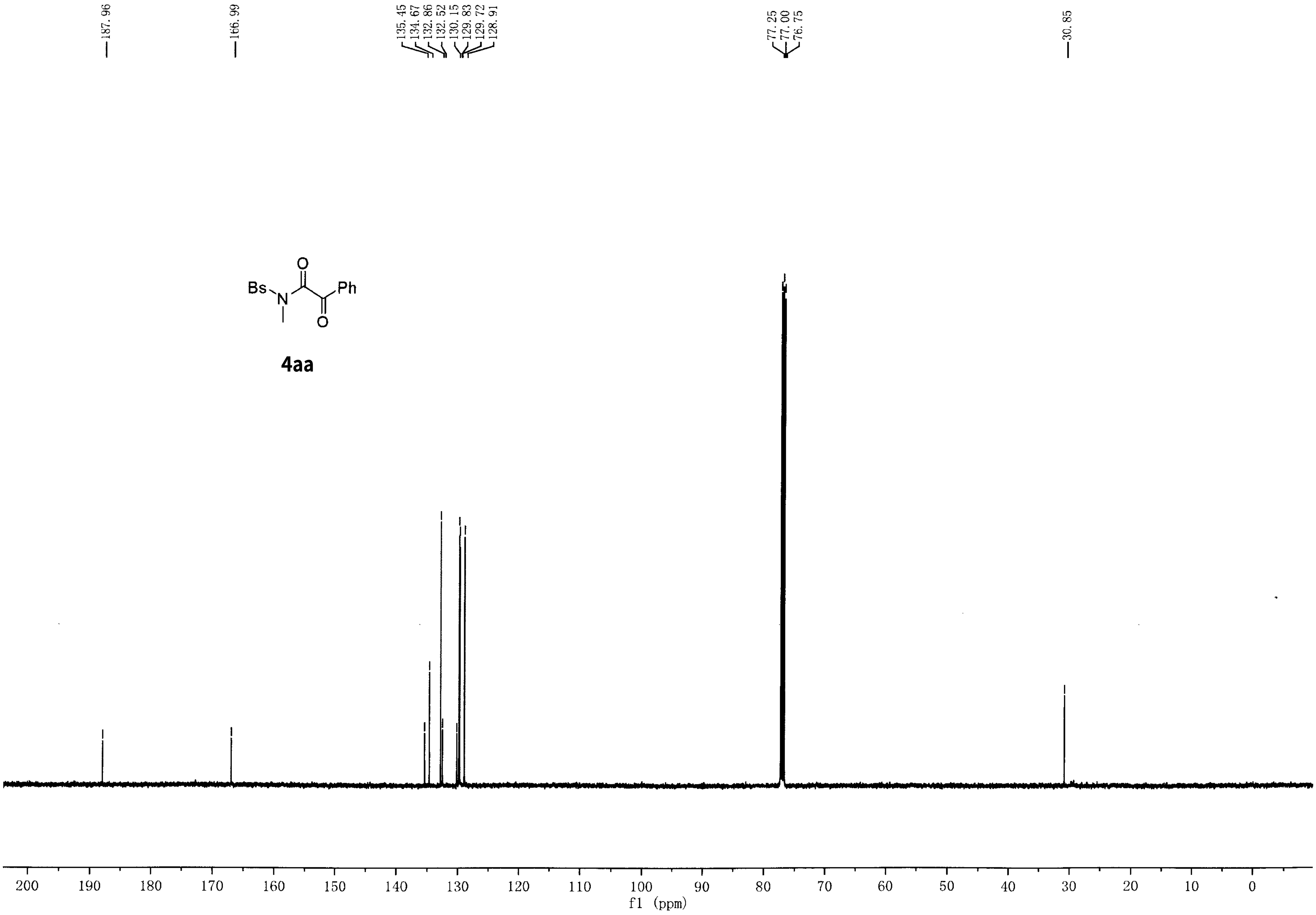

187.96
166.99
135.45
134.67
132.86
132.52
130.15
129.83
129.72
128.91
77.25
77.00
76.75
30.85
Bs
N
O
O
Ph
4aa
200 190 180 170 160 150 140 130 120 110 100 90 80 70 60 50 40 30 20 10 0
f1 (ppm)

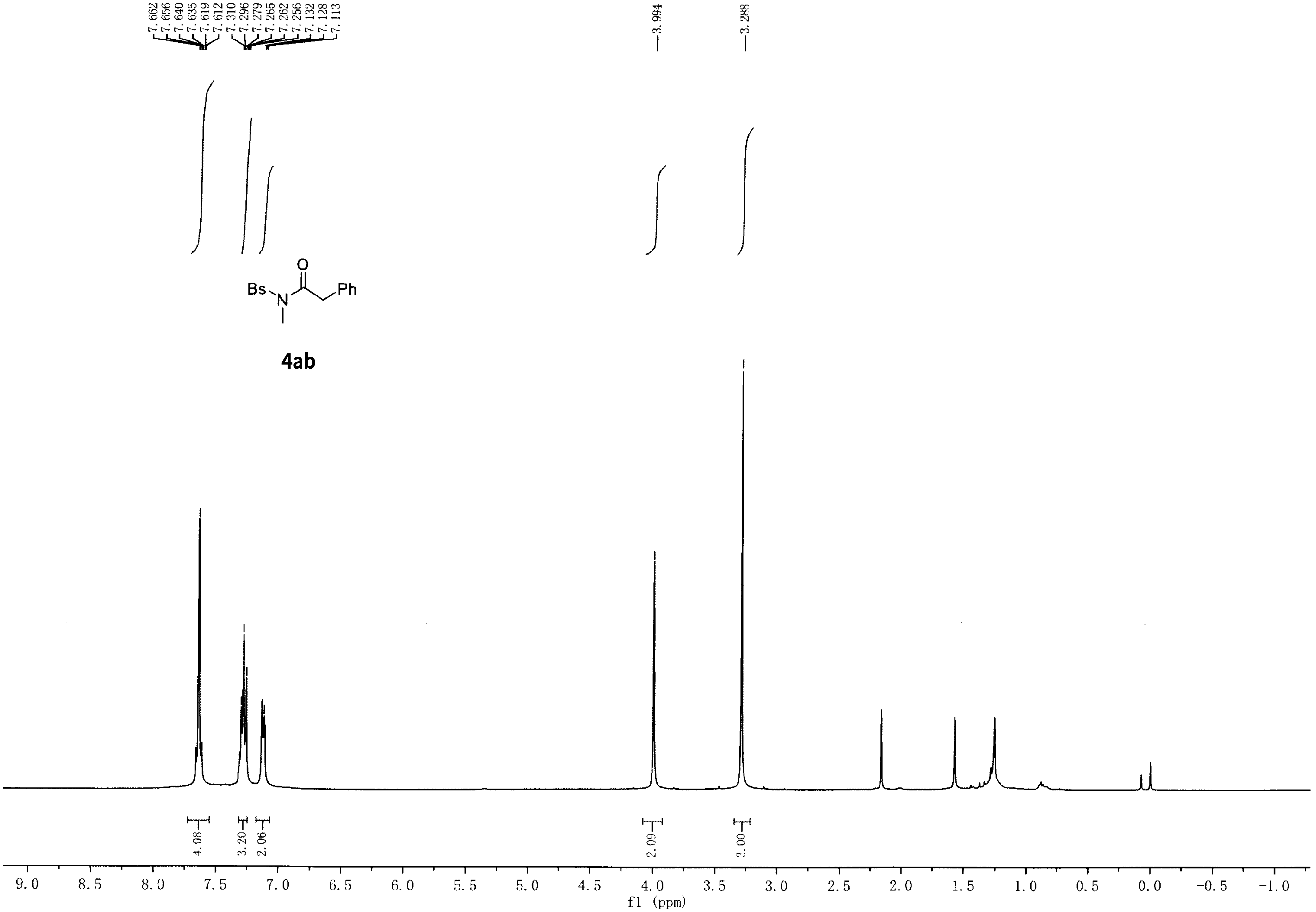

7.662
7.656
7.640
7.635
7.619
7.612
7.310
7.296
7.279
7.265
7.262
7.256
7.132
7.128
7.113
3.994
3.288
O
Bs
N
Ph
4.08
3.20
2.06
2.09
3.00
9.0 8.5 8.0 7.5 7.0 6.5 6.0 5.5 5.0 4.5 4.0 3.5 3.0 2.5 2.0 1.5 1.0 0.5 0.0 -0.5 -1.0
f1 (ppm)

4ab

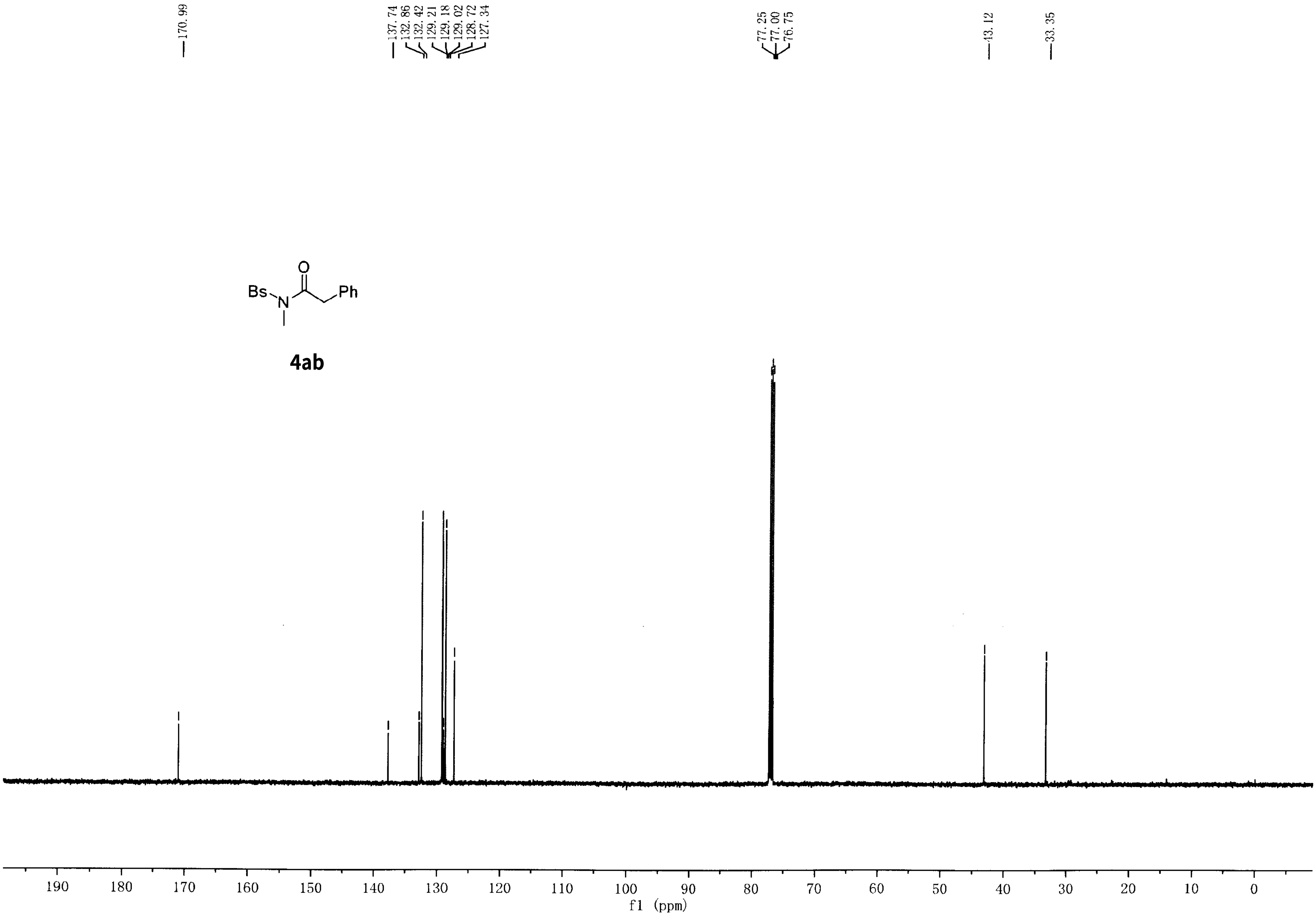

170.99
137.74
132.86
132.42
129.21
129.18
129.02
128.72
127.34
77.25
77.00
76.75
43.12
33.35
190
180
170
160
150
140
130
120
110
100
90
80
70
60
50
40
30
20
10
0
f1 (ppm)

4ab

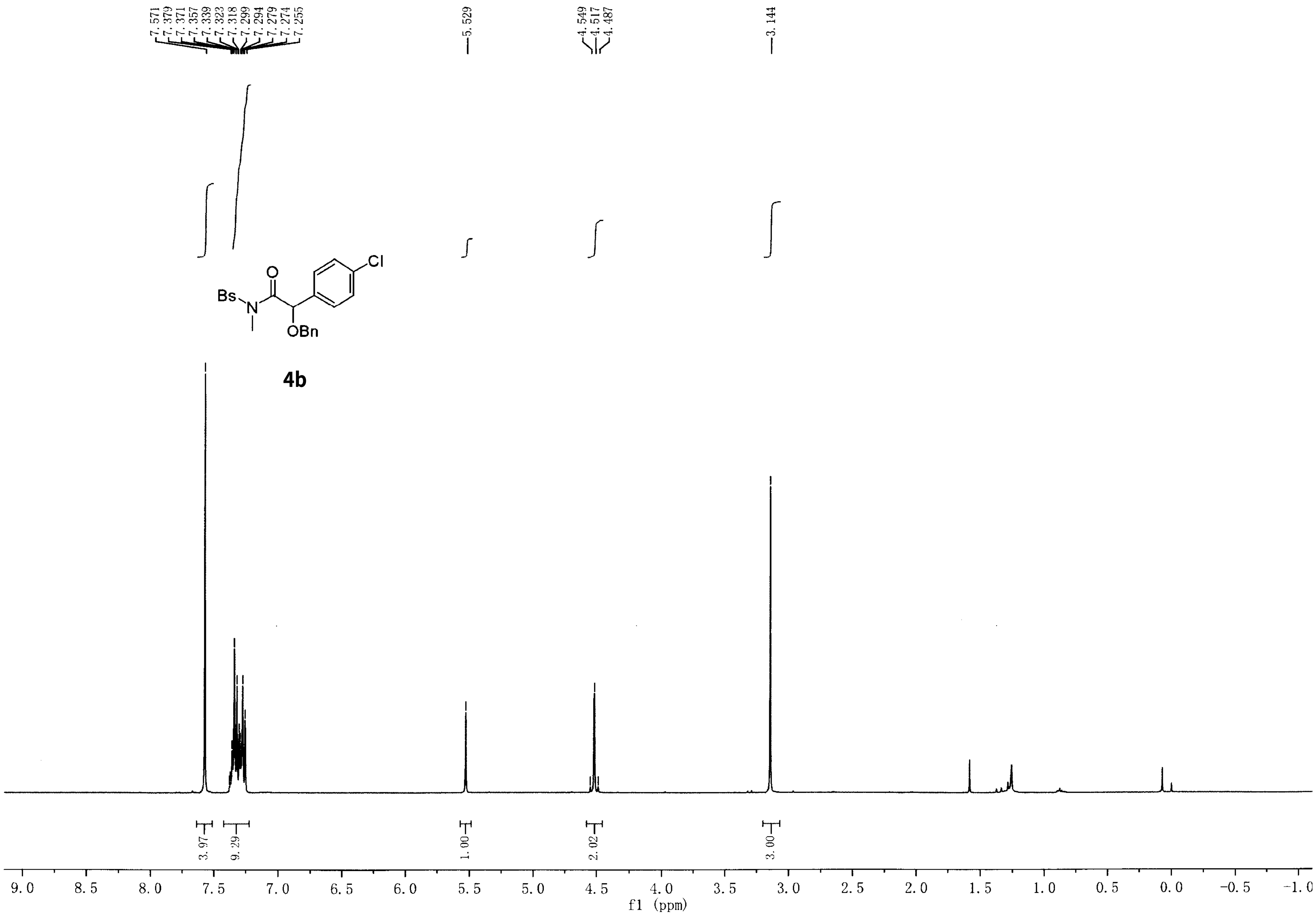

7.571
7.379
7.371
7.357
7.339
7.323
7.318
7.299
7.294
7.279
7.274
7.255
5.529
4.549
4.517
4.487
3.144
Bs
N
O
Cl
OBn
4b
3.97
9.29
1.00
2.02
3.00
9.0 8.5 8.0 7.5 7.0 6.5 6.0 5.5 5.0 4.5 4.0 3.5 3.0 2.5 2.0 1.5 1.0 0.5 0.0 −0.5 −1.0
f1 (ppm)

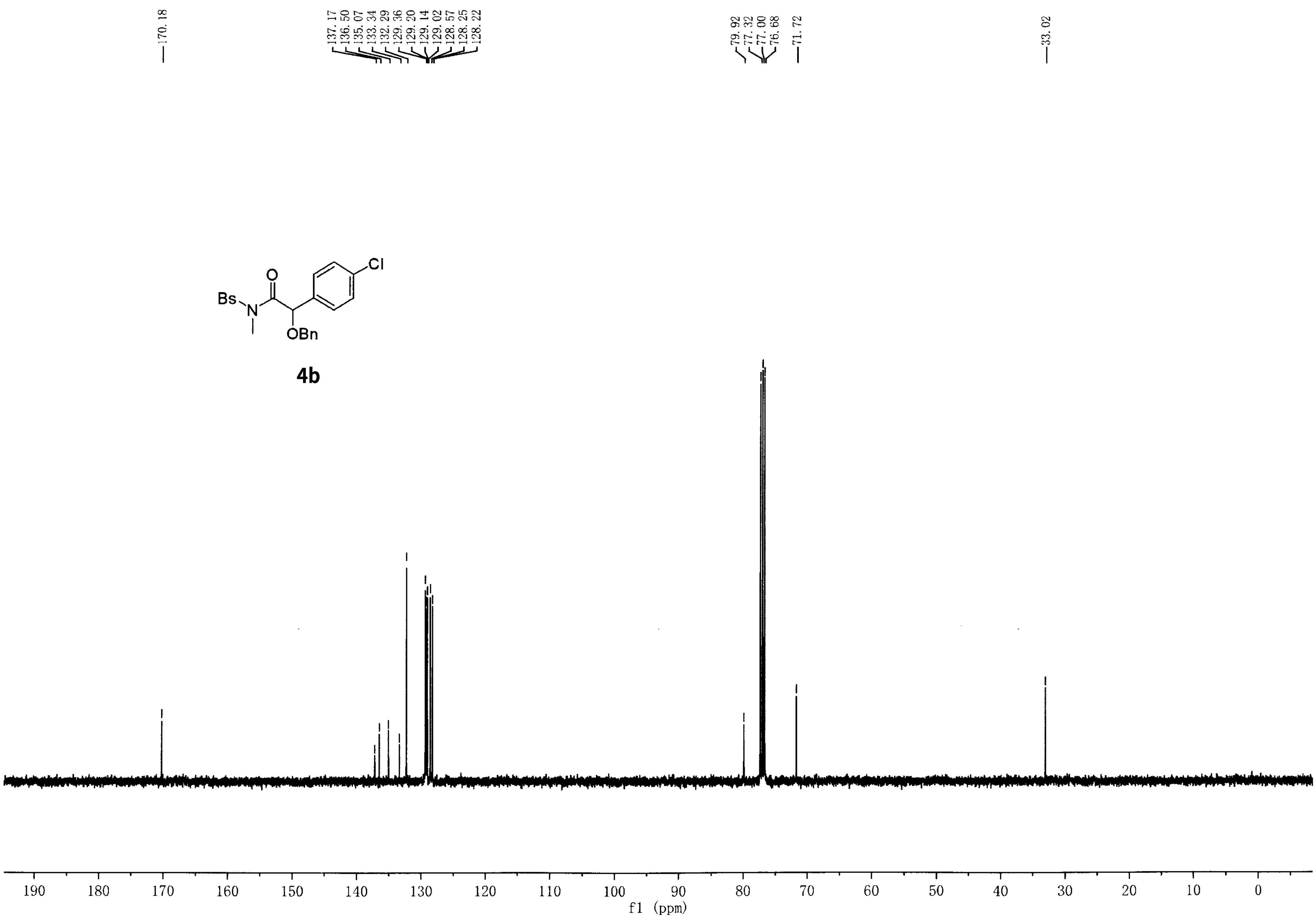
170.18
137.17
136.50
135.07
133.34
132.29
129.36
129.20
129.14
129.02
128.57
128.25
128.22
79.92
77.32
77.00
76.68
71.72
33.02
Bs
N
O
Cl
OBn
4b
190 180 170 160 150 140 130 120 110 100 90 80 70 60 50 40 30 20 10 0
f1 (ppm)

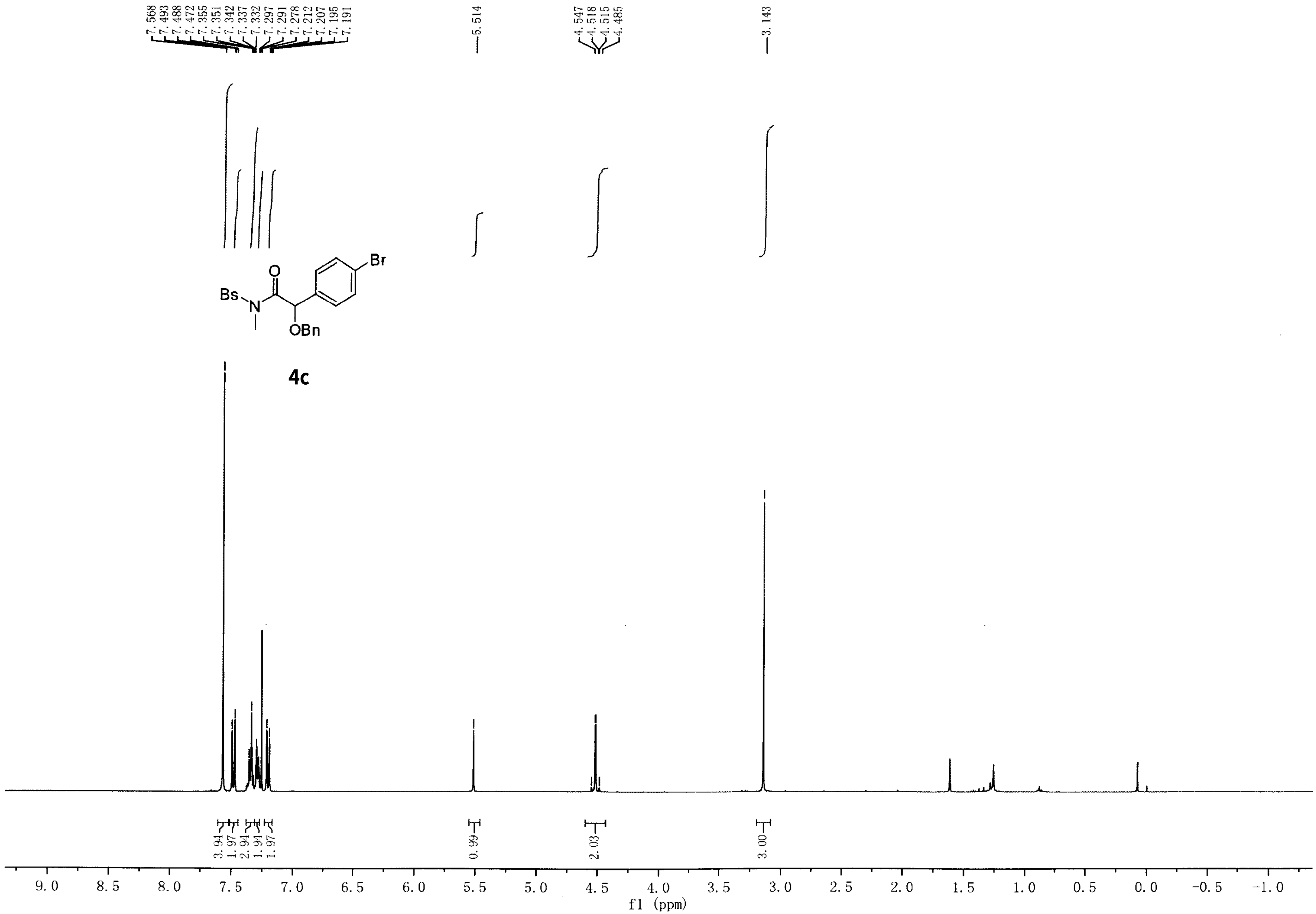
7.568
7.493
7.488
7.472
7.355
7.351
7.342
7.337
7.332
7.297
7.291
7.278
7.212
7.207
7.195
7.191
5.514
4.547
4.518
4.515
4.485
3.143
Br
O
Bs
N
OBn
3.94
1.97
2.94
1.94
1.97
0.99
2.03
3.00
9.0
8.5
8.0
7.5
7.0
6.5
6.0
5.5
5.0
4.5
4.0
3.5
3.0
2.5
2.0
1.5
1.0
0.5
0.0
-0.5
-1.0
f1 (ppm)

4c

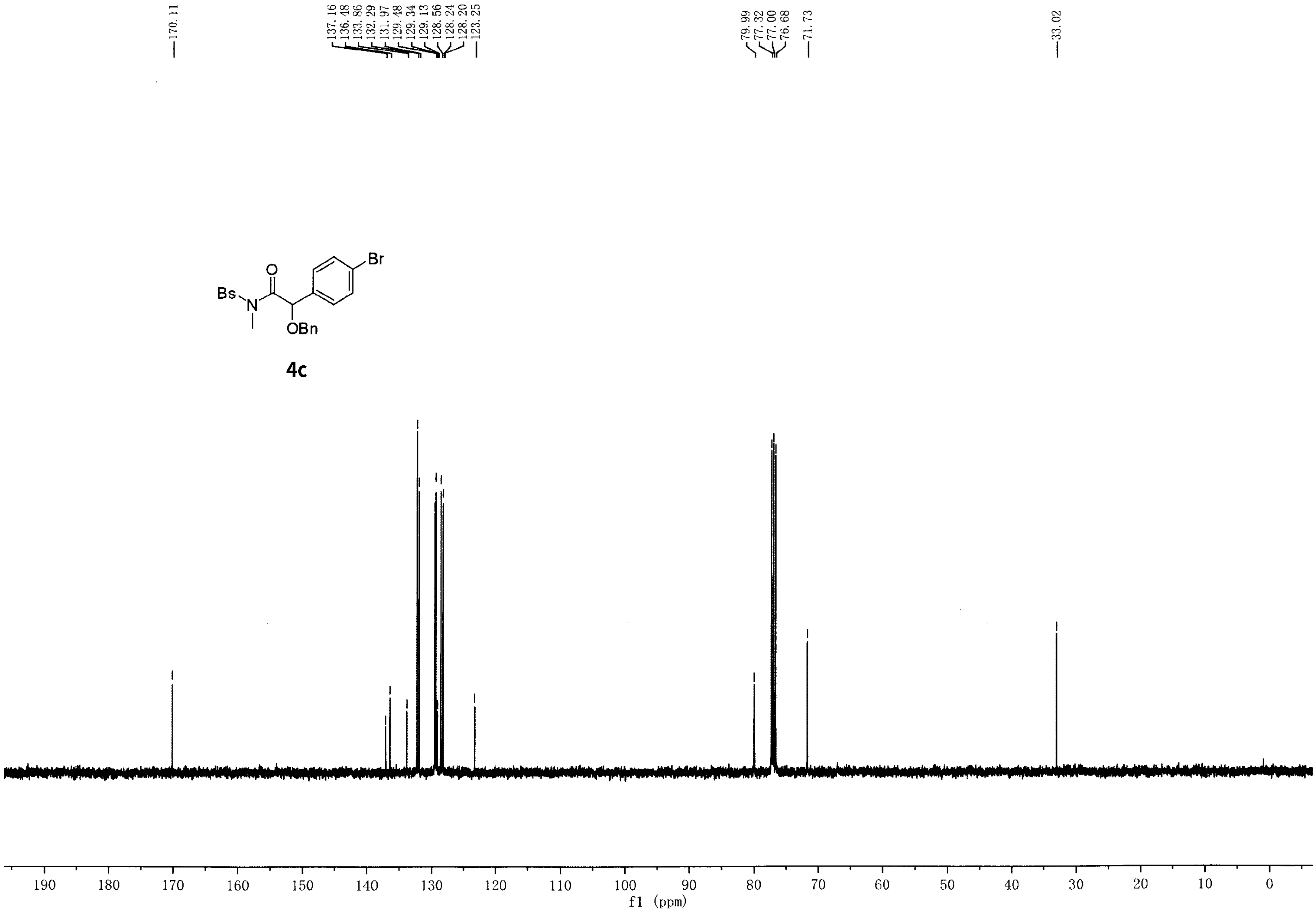
170.11
137.16
136.48
133.86
132.29
131.97
129.48
129.34
129.13
128.56
128.24
128.20
123.25
79.99
77.32
77.00
76.68
71.73
33.02
Br
O
Bs
N
OBn
4c
190 180 170 160 150 140 130 120 110 100 90 80 70 60 50 40 30 20 10 0
f1 (ppm)

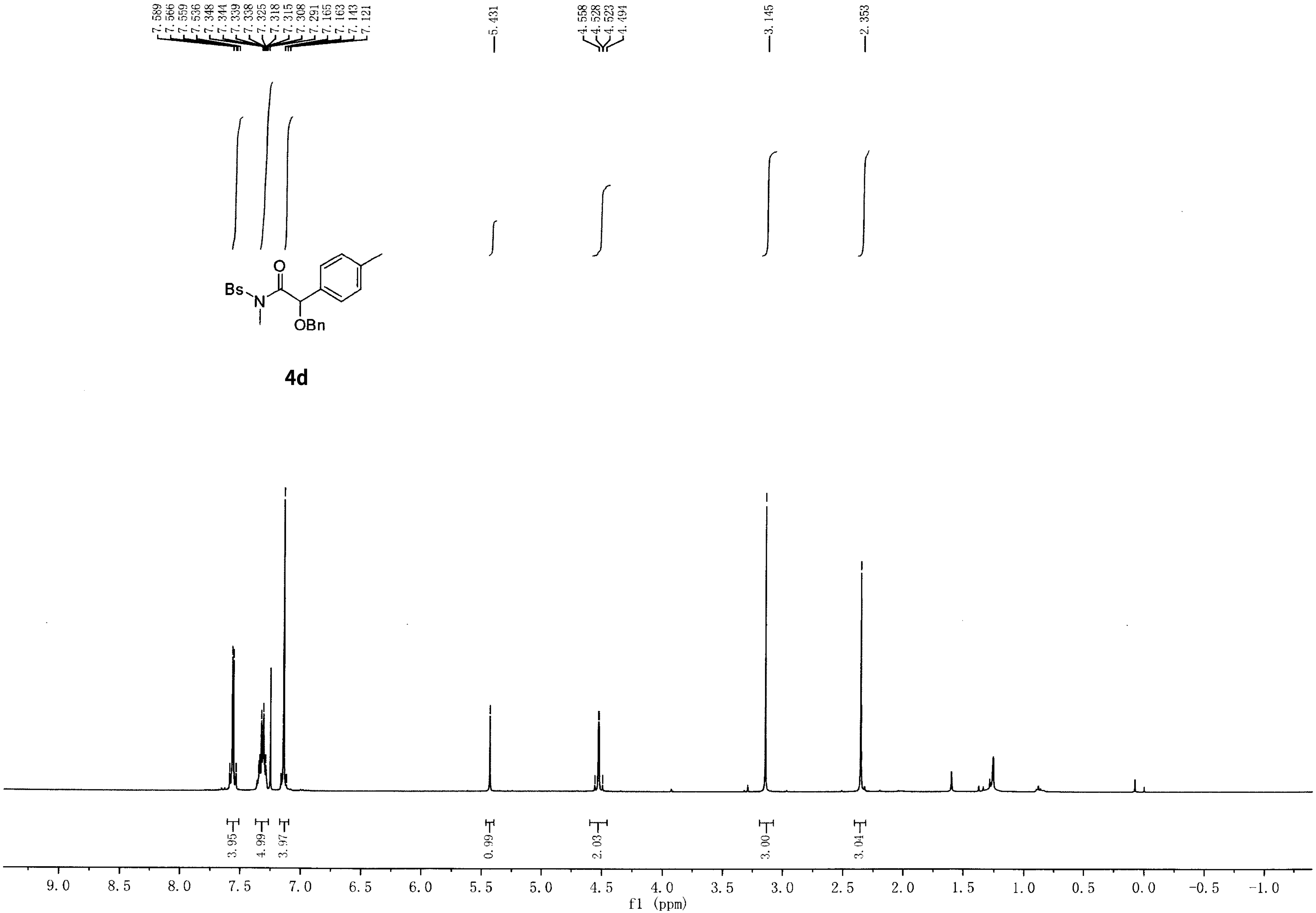

7.589
7.566
7.559
7.536
7.348
7.344
7.339
7.338
7.325
7.318
7.315
7.308
7.291
7.165
7.163
7.143
7.121
5.431
4.558
4.528
4.523
4.494
3.145
2.353
Bs
N
O
OBn
4d
3.95
4.99
3.97
0.99
2.03
3.00
3.04
9.0
8.5
8.0
7.5
7.0
6.5
6.0
5.5
5.0
4.5
4.0
3.5
3.0
2.5
2.0
1.5
1.0
0.5
0.0
-0.5
-1.0
f1 (ppm)

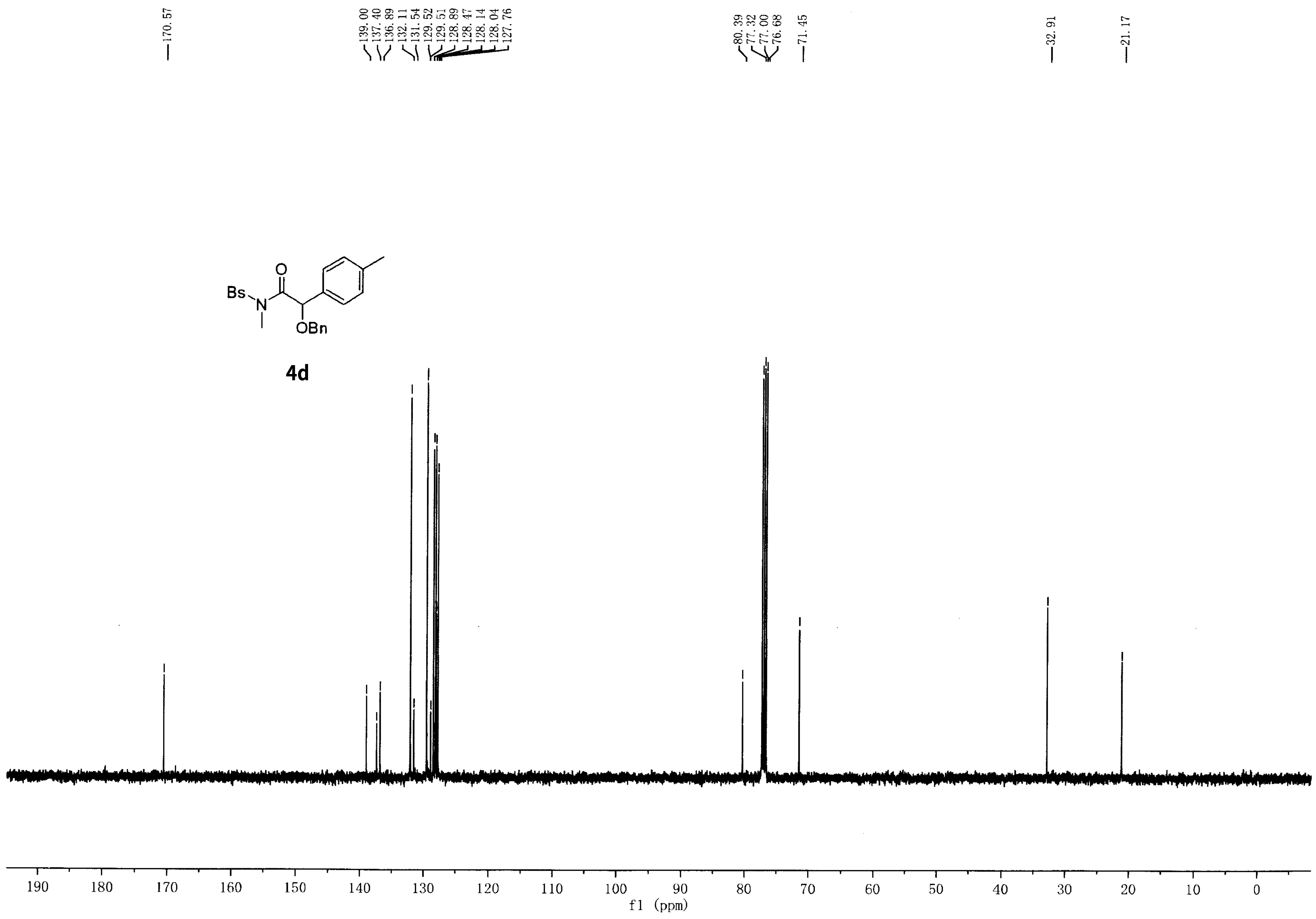

170.57
139.00
137.40
136.89
132.11
131.54
129.52
129.51
128.89
128.47
128.14
128.04
127.76
80.39
77.32
77.00
76.68
71.45
32.91
21.17
Bs
N
O
OBn
4d
190 180 170 160 150 140 130 120 110 100 90 80 70 60 50 40 30 20 10 0
f1 (ppm)

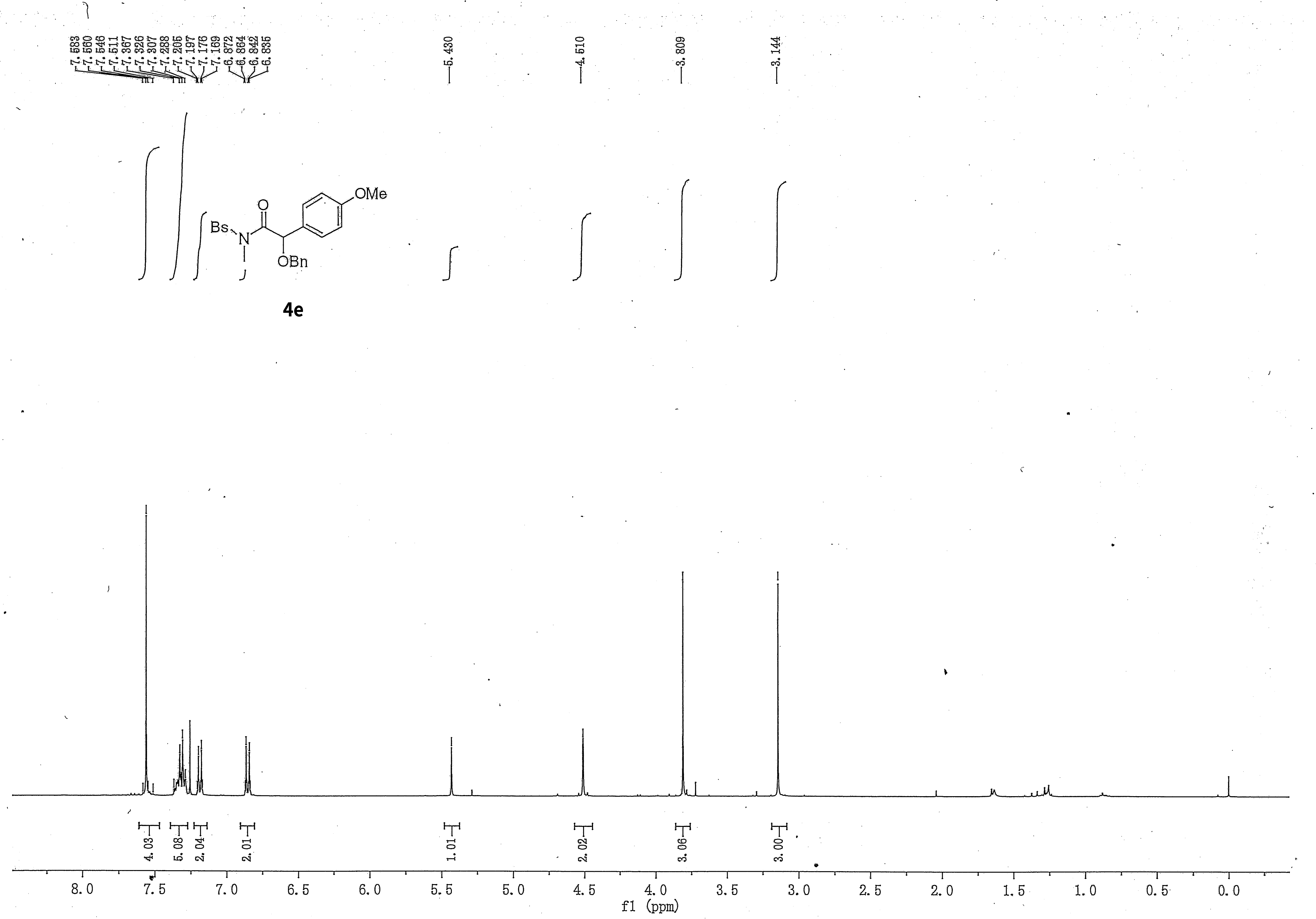

7.583
7.560
7.546
7.511
7.367
7.326
7.307
7.288
7.205
7.197
7.176
7.169
6.872
6.864
6.842
6.835
5.430
4.510
3.809
3.144
OMe
O
Bs
N
OBn
4e
4.03
5.08
2.04
2.01
1.01
2.02
3.06
3.00
8.0
7.5
7.0
6.5
6.0
5.5
5.0
4.5
4.0
3.5
3.0
2.5
2.0
1.5
1.0
0.5
0.0
f1 (ppm)

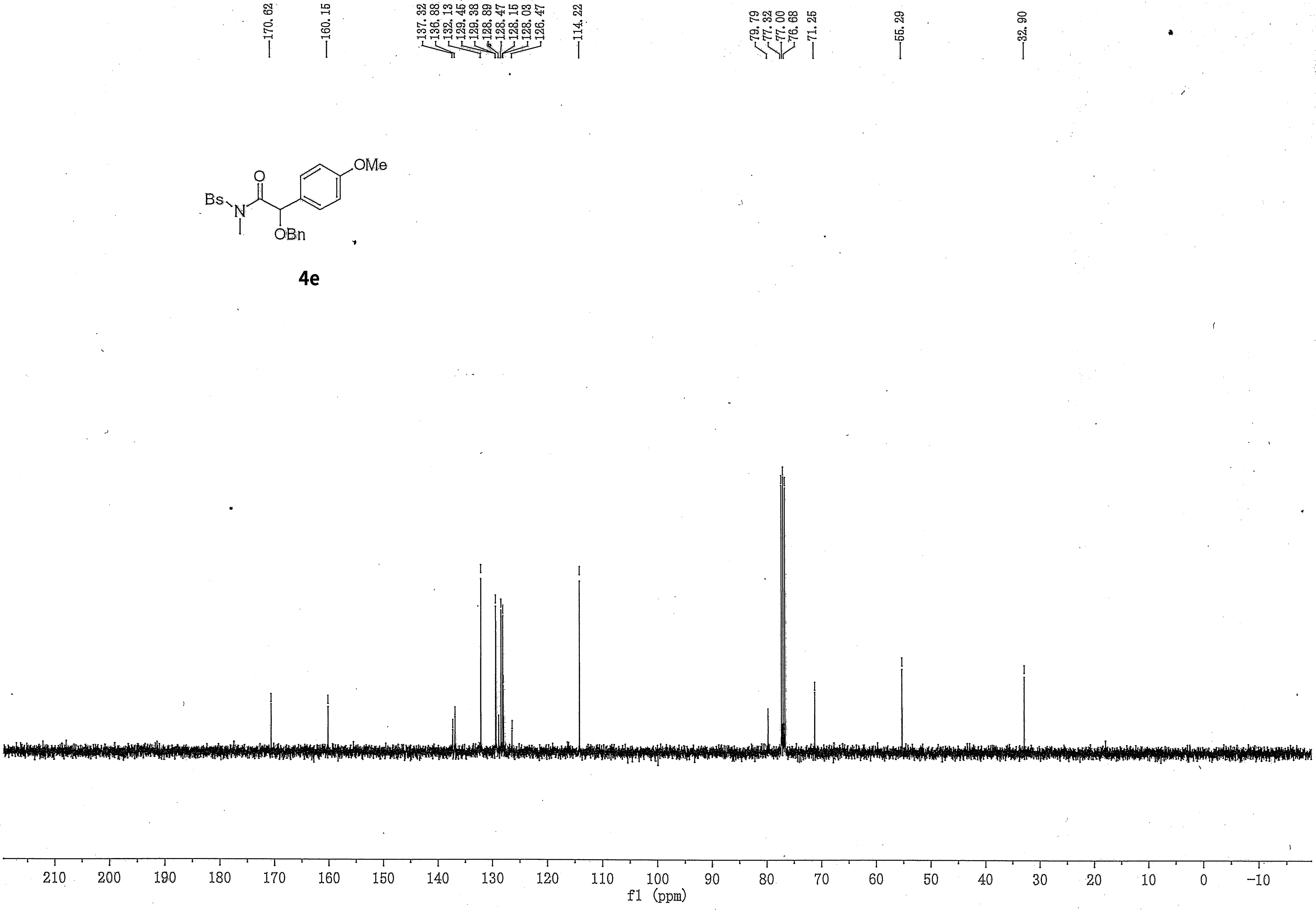

170.62
160.15
137.32
136.88
132.13
129.45
129.38
128.89
128.47
128.15
128.03
126.47
114.22
79.79
77.32
77.00
76.68
71.25
55.29
32.90
Bs
N
O
OMe
OBn
4e
210 200 190 180 170 160 150 140 130 120 110 100 90 80 70 60 50 40 30 20 10 0 -10
f1 (ppm)

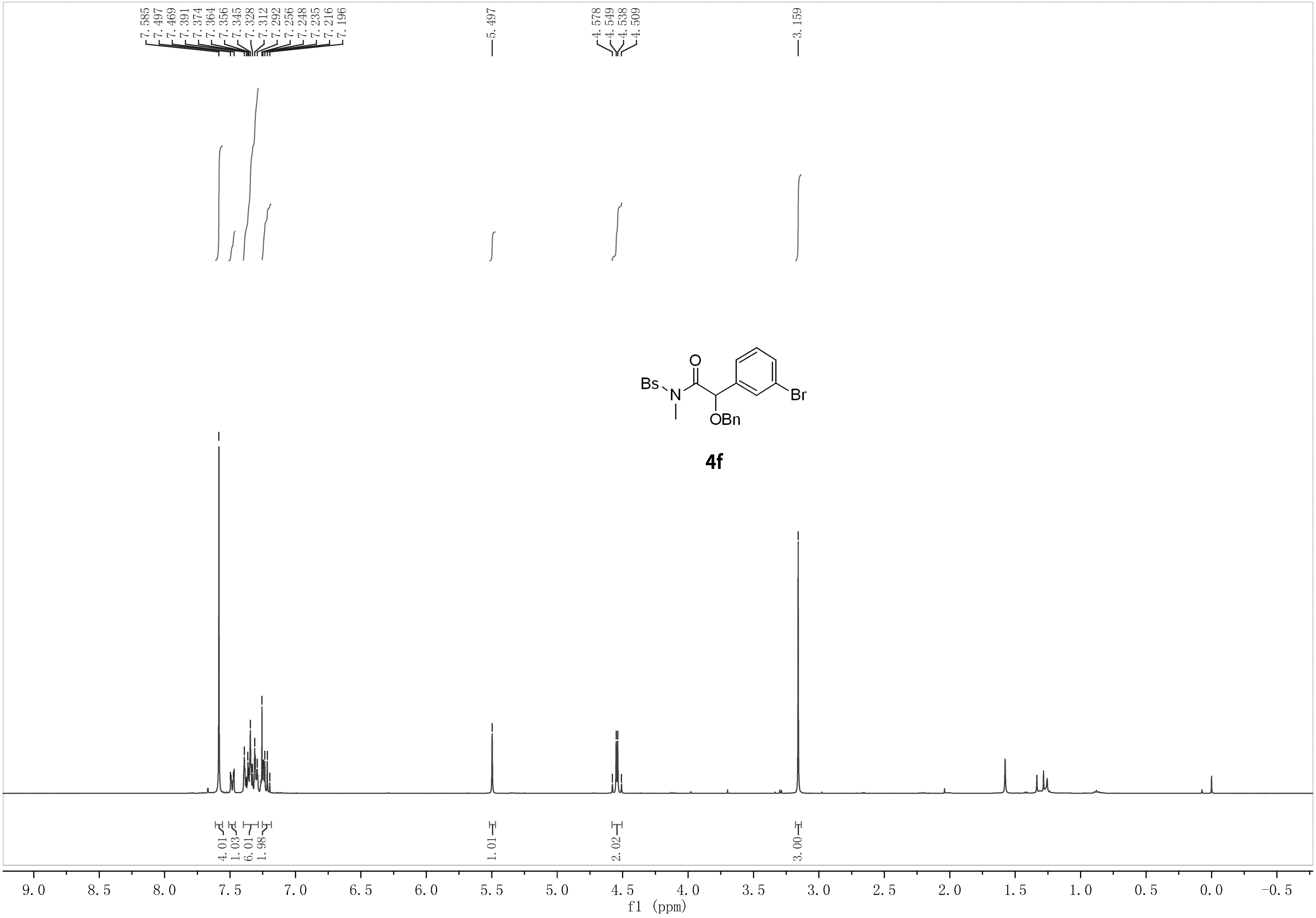
7.585
7.497
7.469
7.391
7.374
7.364
7.356
7.345
7.328
7.312
7.292
7.256
7.248
7.235
7.216
7.196
5.497
4.578
4.549
4.538
4.509
3.159
Bs
N
O
OBn
Br
4f
4.01
1.03
6.01
1.98
1.01
2.02
3.00
9.0 8.5 8.0 7.5 7.0 6.5 6.0 5.5 5.0 4.5 4.0 3.5 3.0 2.5 2.0 1.5 1.0 0.5 0.0 −0.5
f1 (ppm)

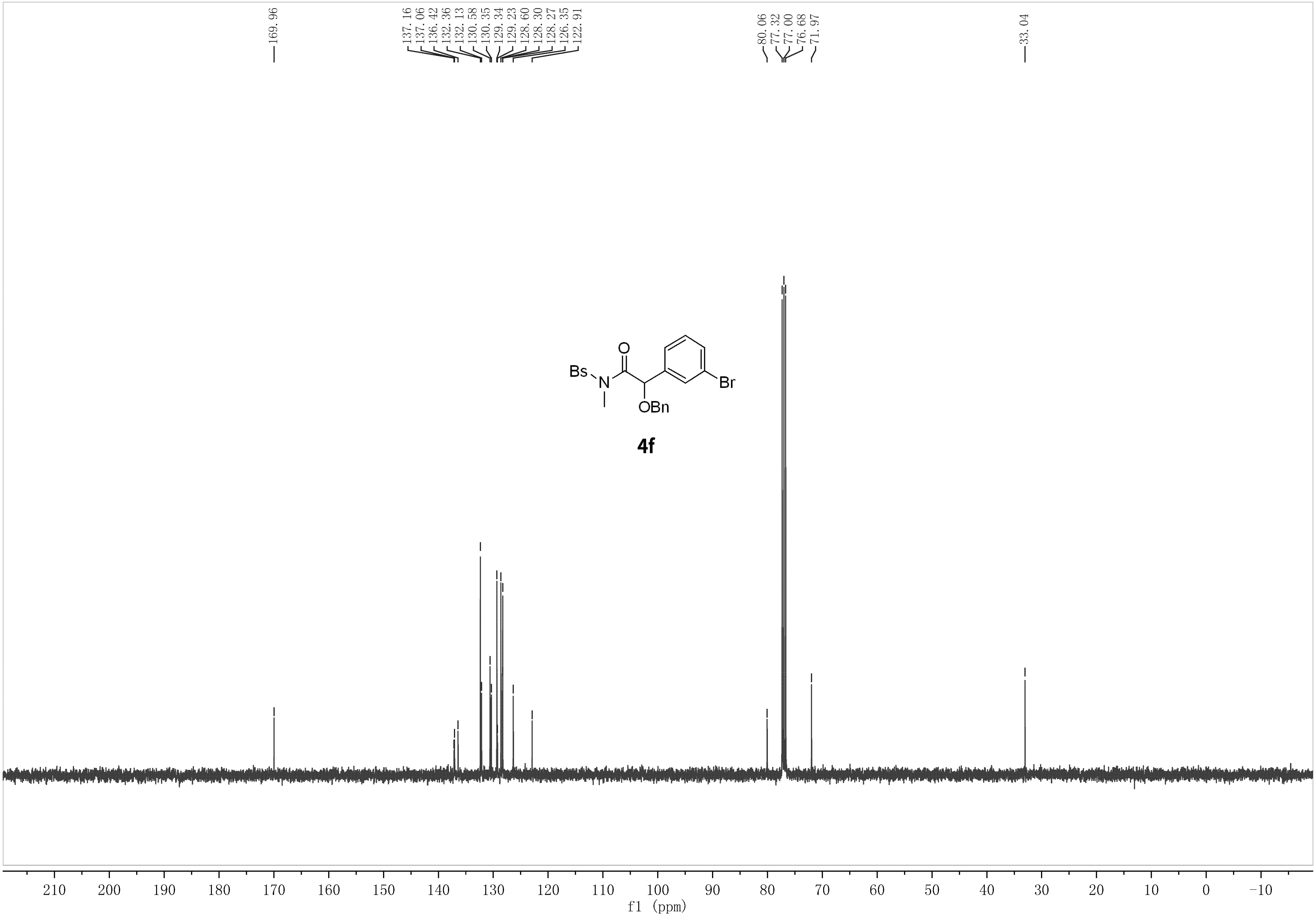

169.96
137.16
137.06
136.42
132.36
132.13
130.58
130.35
129.34
129.23
128.60
128.30
128.27
126.35
122.91
80.06
77.32
77.00
76.68
71.97
33.04
O
Bs
N
OBn
Br
4f
210 200 190 180 170 160 150 140 130 120 110 100 90 80 70 60 50 40 30 20 10 0 −10
f1 (ppm)

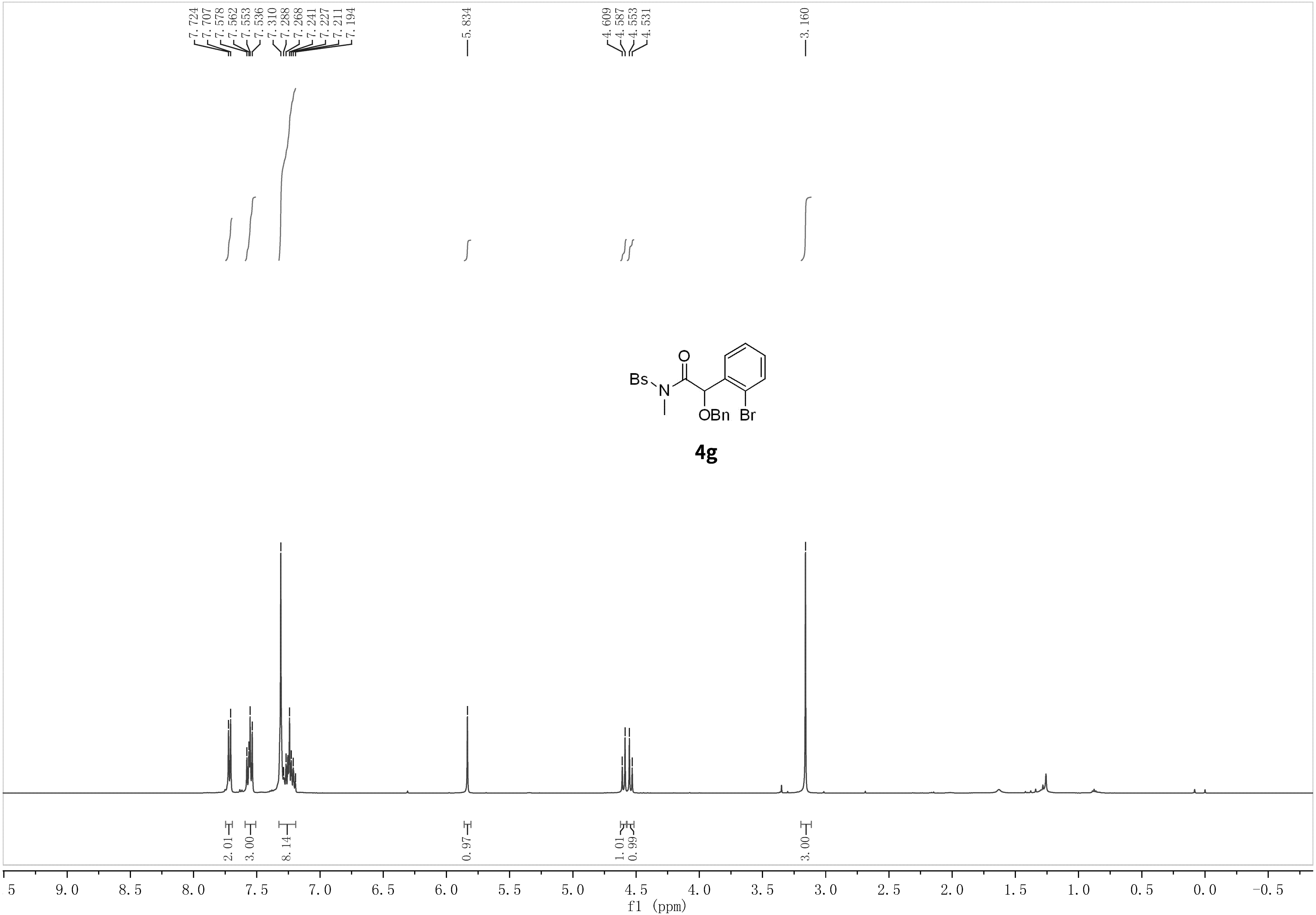

7.724
7.707
7.578
7.562
7.553
7.536
7.310
7.288
7.268
7.241
7.227
7.211
7.194
5.834
4.609
4.587
4.553
4.531
3.160
Bs
N
O
OBn
Br
4g
2.01
3.00
8.14
0.97
1.01
0.99
3.00
9.0
8.5
8.0
7.5
7.0
6.5
6.0
5.5
5.0
4.5
4.0
3.5
3.0
2.5
2.0
1.5
1.0
0.5
0.0
-0.5
f1 (ppm)

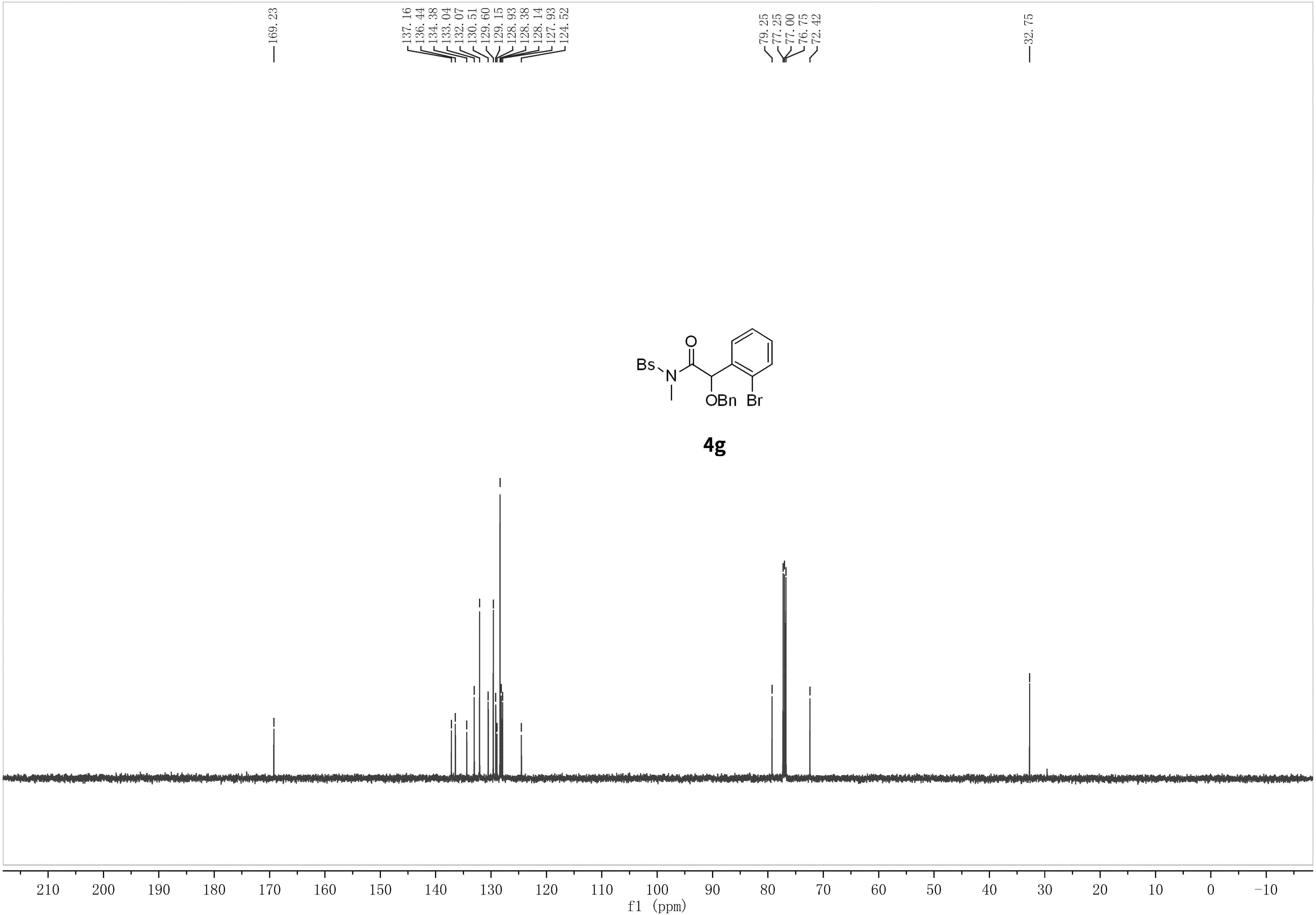

169.23
137.16
136.44
134.38
133.04
132.07
130.51
129.60
129.15
128.93
128.38
128.14
127.93
124.52
79.25
77.25
77.00
76.75
72.42
32.75
Bs
N
O
OBn
Br
4g
210 200 190 180 170 160 150 140 130 120 110 100 90 80 70 60 50 40 30 20 10 0 −10
f1 (ppm)

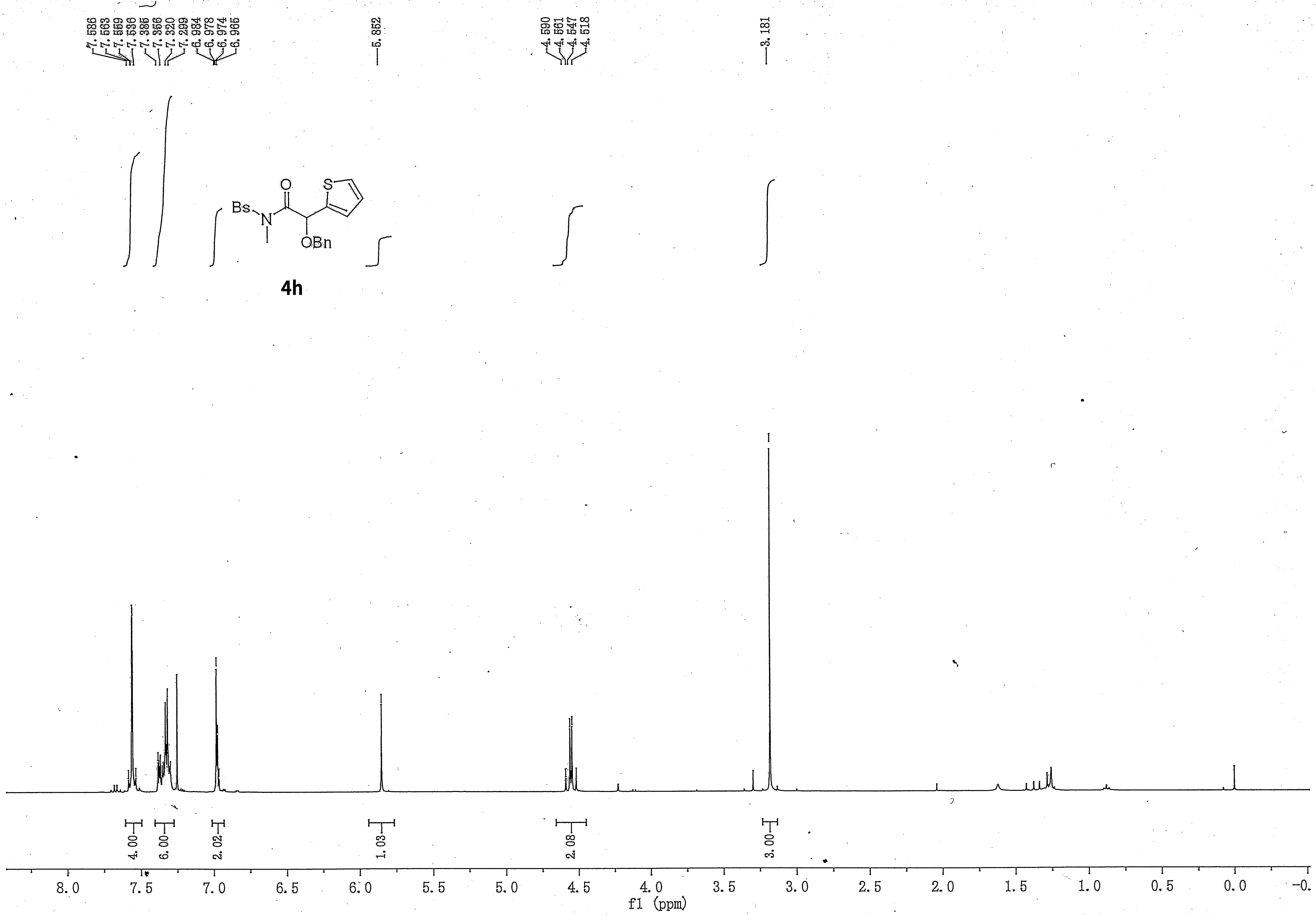
7.586
7.563
7.569
7.536
7.385
7.356
7.320
7.299
6.984
6.978
6.974
6.965
5.852
4.590
4.561
4.547
4.518
3.181
Bs
N
O
S
OBn
4h
4.00
6.00
2.02
1.03
2.08
3.00
8.0
7.5
7.0
6.5
6.0
5.5
5.0
4.5
4.0
3.5
3.0
2.5
2.0
1.5
1.0
0.5
0.0
-0.
f1 (ppm)

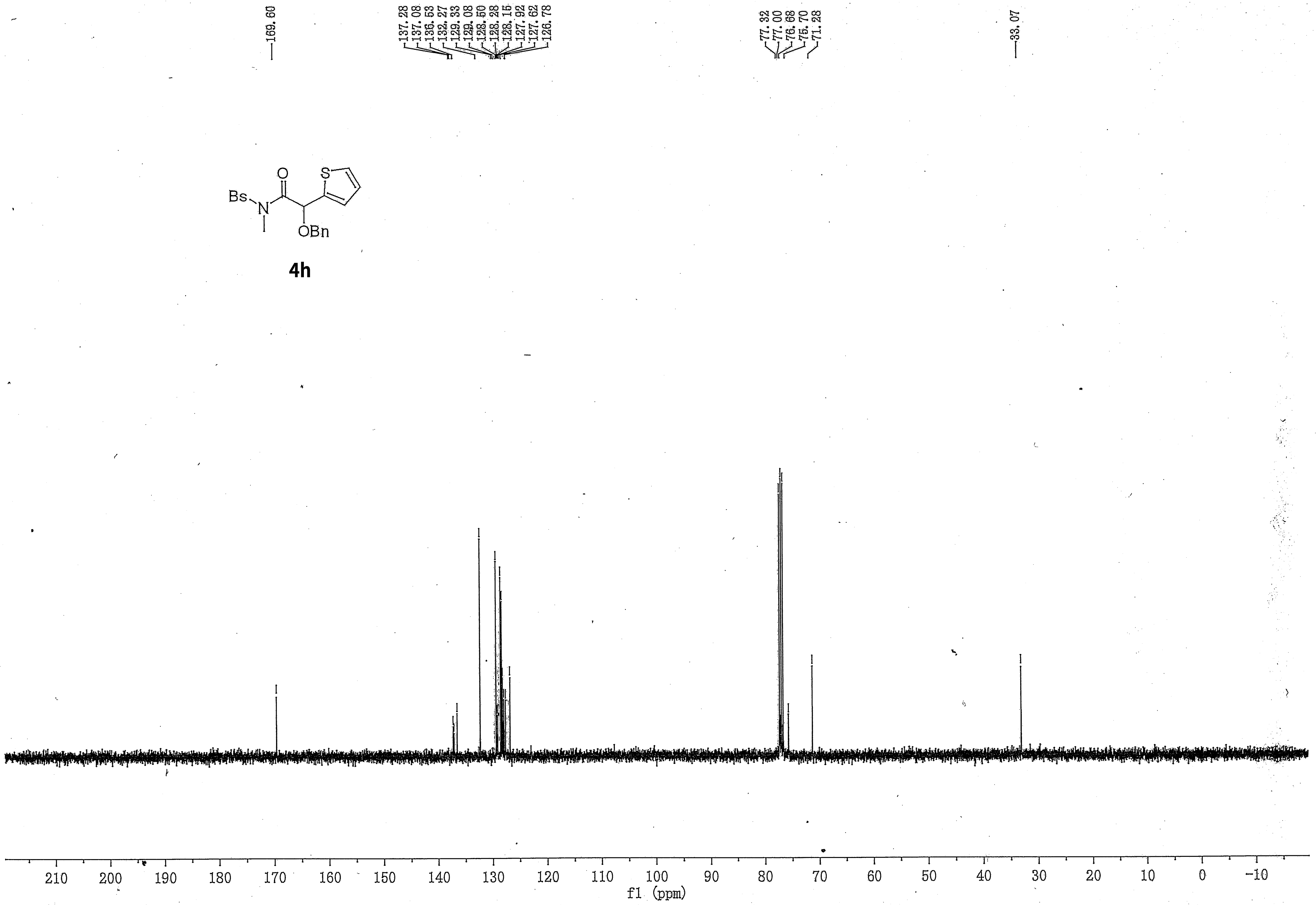

169.60
137.28
137.08
136.53
132.27
129.33
129.08
128.50
128.28
128.15
127.92
127.62
126.78
77.32
77.00
76.68
75.70
71.28
33.07
Bs
N
O
S
OBn
210 200 190 180 170 160 150 140 130 120 110 100 90 80 70 60 50 40 30 20 10 0 −10
f1 (ppm)

4h

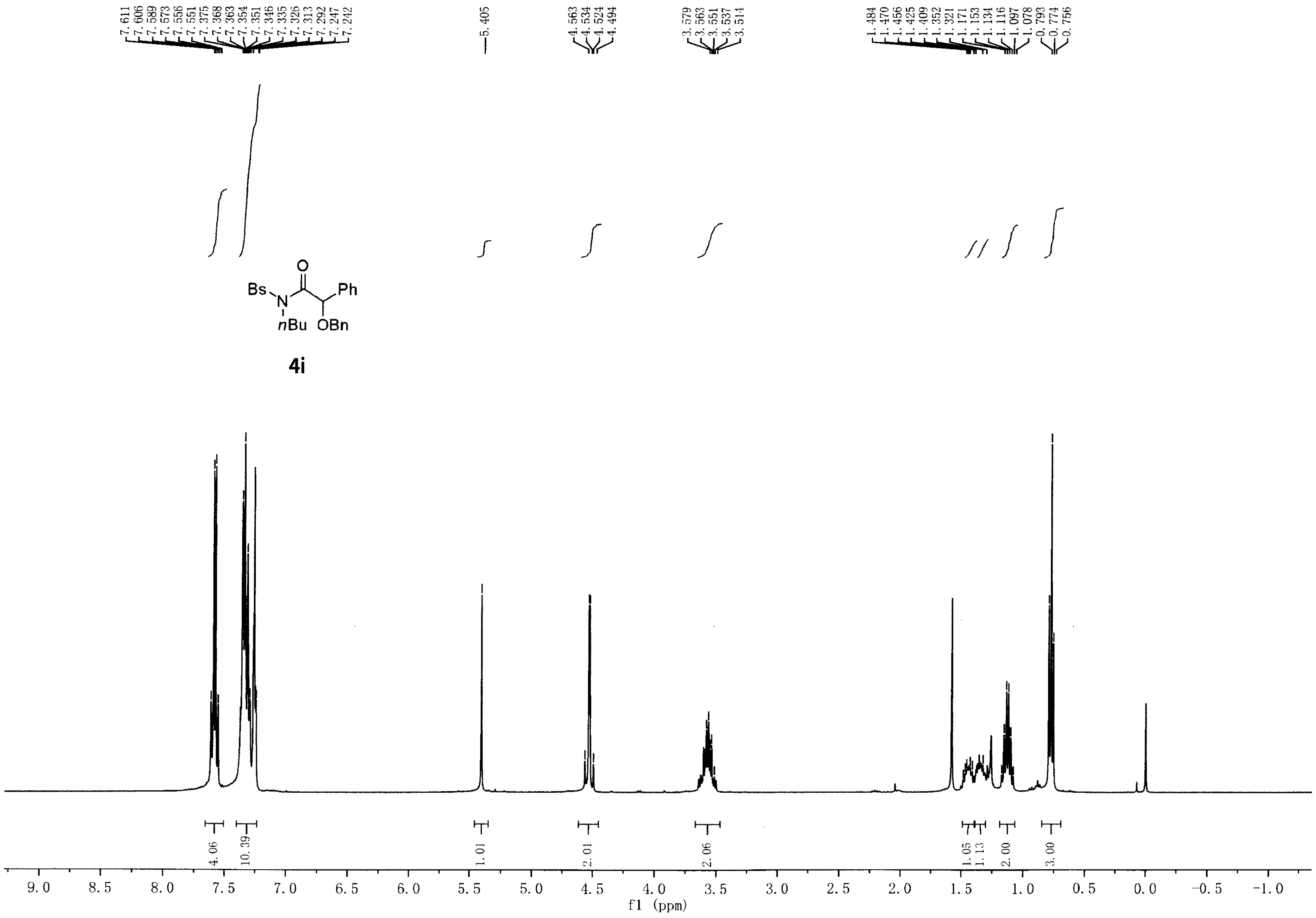

7.611 7.606 7.589 7.573 7.556 7.551 7.375 7.368 7.363 7.354 7.351 7.346 7.335 7.326 7.313 7.292 7.247 7.242
5.405
4.563 4.534 4.524 4.494
3.579 3.563 3.551 3.537 3.514
1.484 1.470 1.456 1.425 1.409 1.352 1.321 1.171 1.153 1.134 1.116 1.097 1.078 0.793 0.774 0.756
Bs
N
O
Ph
nBu
OBn
4i
4.06
10.39
1.01
2.01
2.06
1.05
1.13
2.00
3.00
9.0 8.5 8.0 7.5 7.0 6.5 6.0 5.5 5.0 4.5 4.0 3.5 3.0 2.5 2.0 1.5 1.0 0.5 0.0 -0.5 -1.0
f1 (ppm)

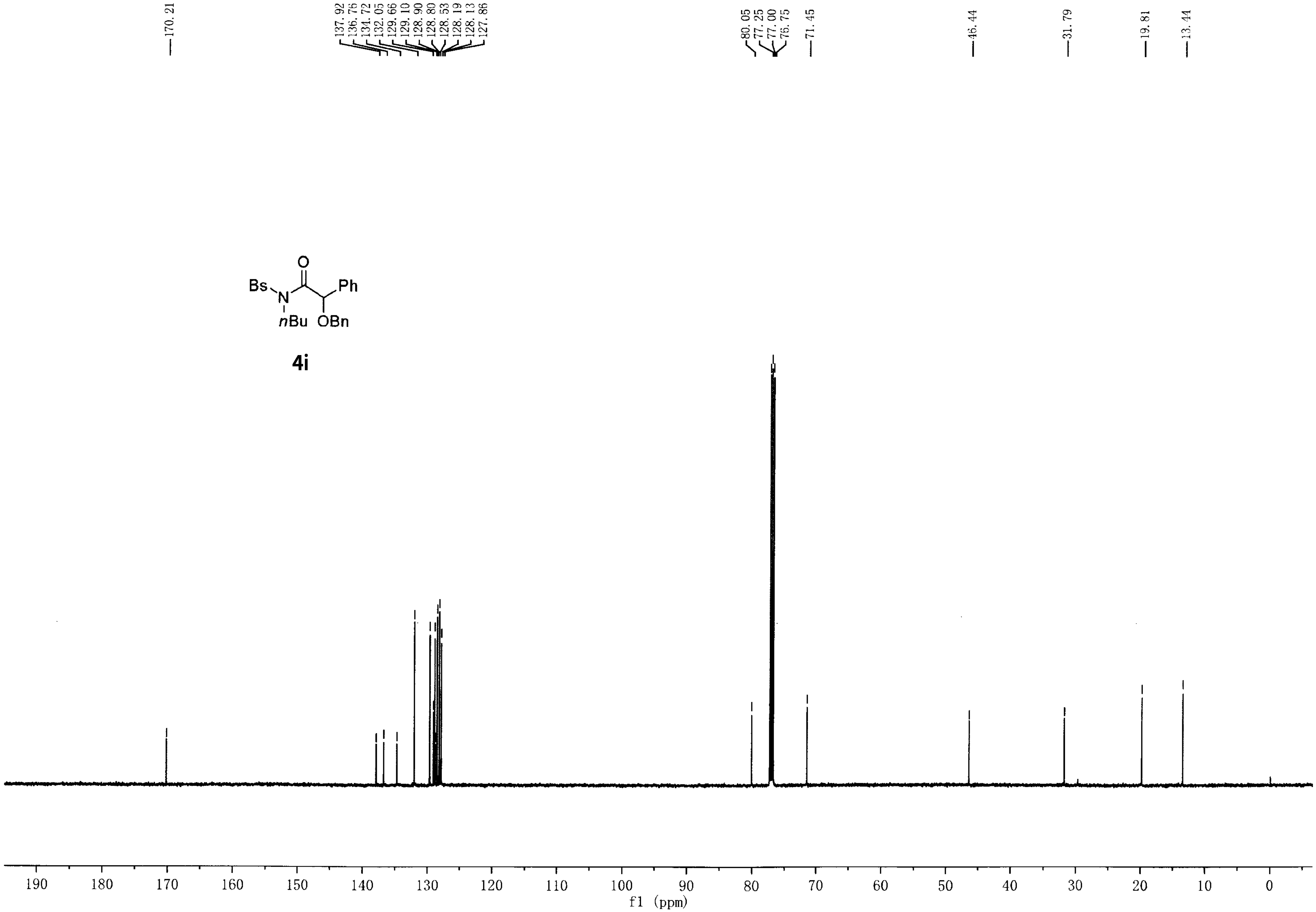
170.21
137.92
136.76
134.72
132.05
129.66
129.10
128.90
128.80
128.53
128.19
128.13
127.86
80.05
77.25
77.00
76.75
71.45
46.44
31.79
19.81
13.44
O
Bs
N
Ph
nBu
OBn
190
180
170
160
150
140
130
120
110
100
90
80
70
60
50
40
30
20
10
0
f1 (ppm)

4i

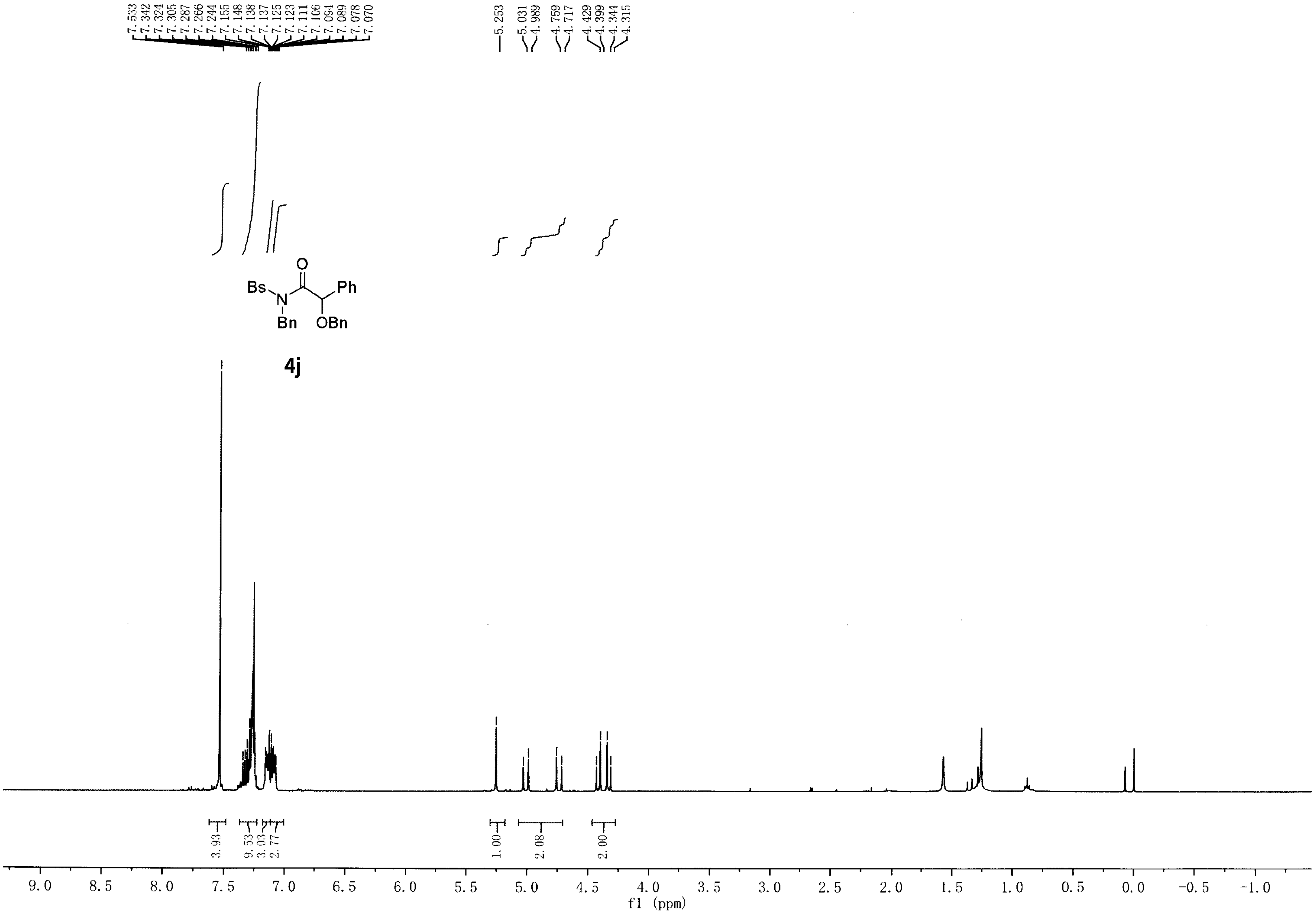

7.533
7.342
7.324
7.305
7.287
7.266
7.244
7.155
7.148
7.138
7.137
7.125
7.123
7.111
7.106
7.094
7.089
7.078
7.070
5.253
5.031
4.989
4.759
4.717
4.429
4.399
4.344
4.315
Bs
N
Bn
O
Ph
OBn
4j
3.93
9.53
3.03
2.77
1.00
2.08
2.00
9.0
8.5
8.0
7.5
7.0
6.5
6.0
5.5
5.0
4.5
4.0
3.5
3.0
2.5
2.0
1.5
1.0
0.5
0.0
-0.5
-1.0
f1 (ppm)

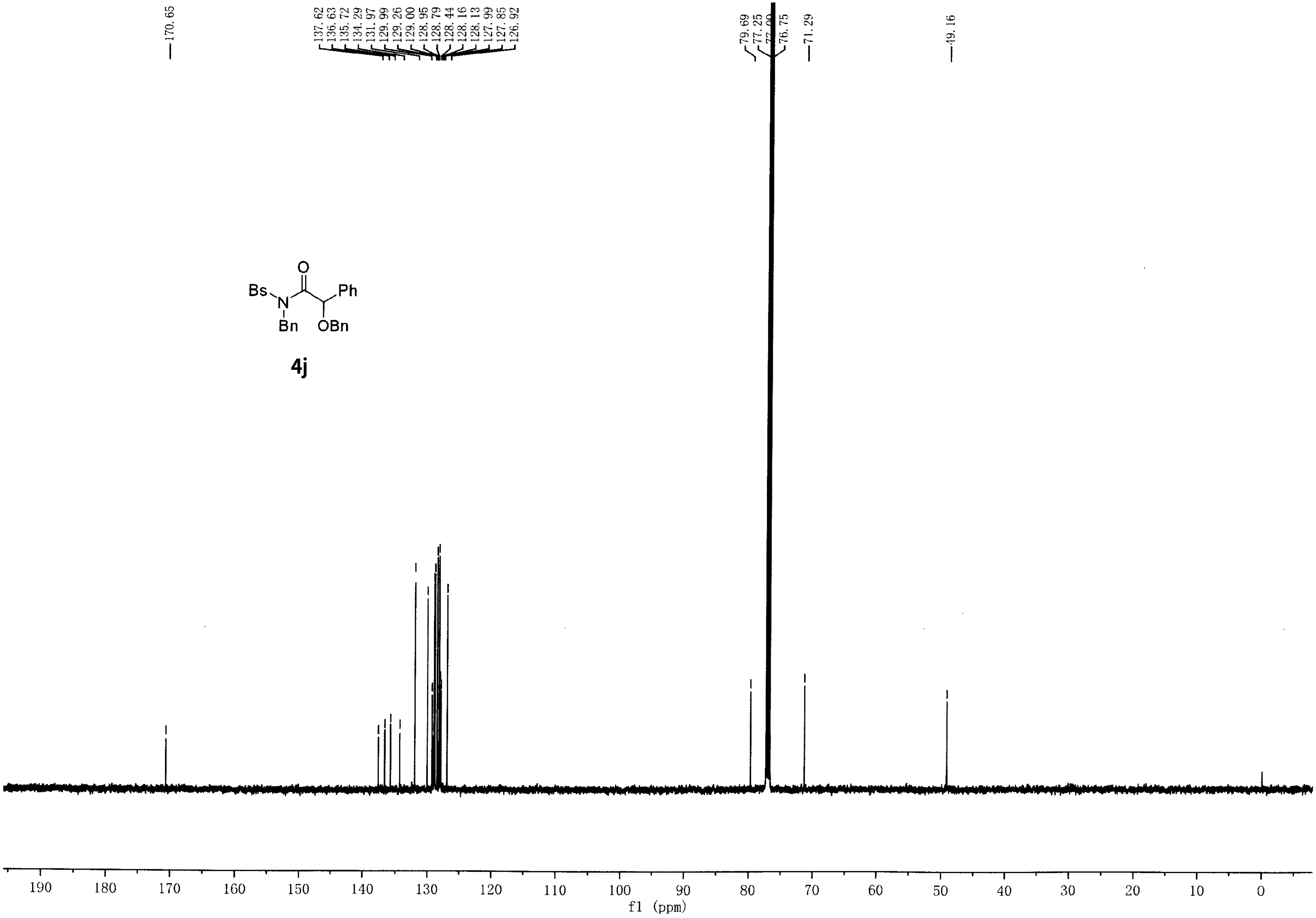
170.65
137.62
136.63
135.72
134.29
131.97
129.99
129.26
129.00
128.95
128.79
128.44
128.16
128.13
127.99
127.85
126.92
79.69
77.25
77.00
76.75
71.29
49.16
Bs
N
Bn
O
Ph
OBn
4j
190 180 170 160 150 140 130 120 110 100 90 80 70 60 50 40 30 20 10 0
f1 (ppm)

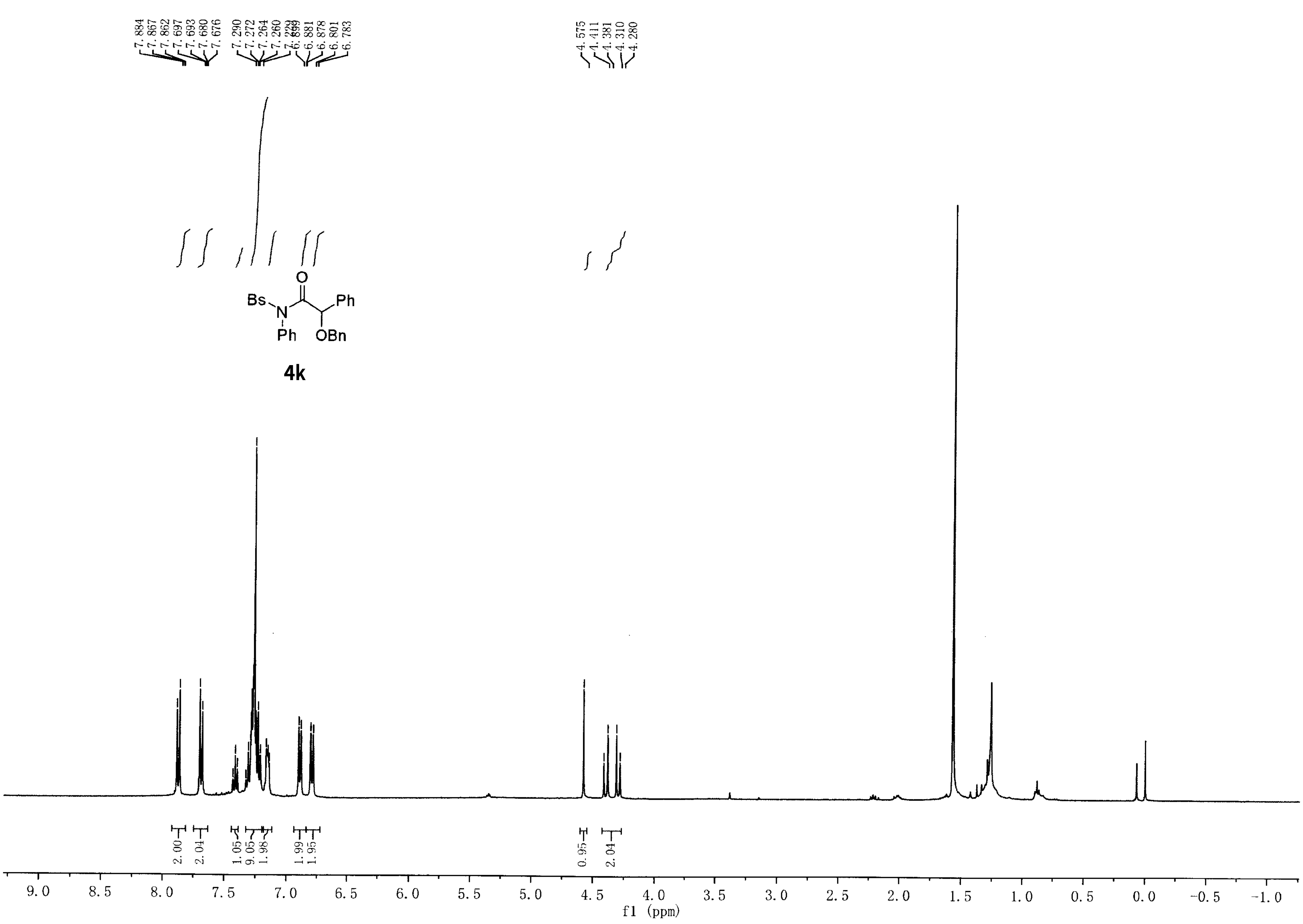
7.884
7.867
7.862
7.697
7.693
7.680
7.676
7.290
7.272
7.264
7.260
6.881
6.878
6.801
6.783
4.575
4.411
4.381
4.310
4.280
Bs
N
Ph
O
Ph
OBn
4k
2.00
2.04
1.05
9.05
1.98
1.99
1.95
0.95
2.04
9.0
8.5
8.0
7.5
7.0
6.5
6.0
5.5
5.0
4.5
4.0
3.5
3.0
2.5
2.0
1.5
1.0
0.5
0.0
-0.5
-1.0
f1 (ppm)

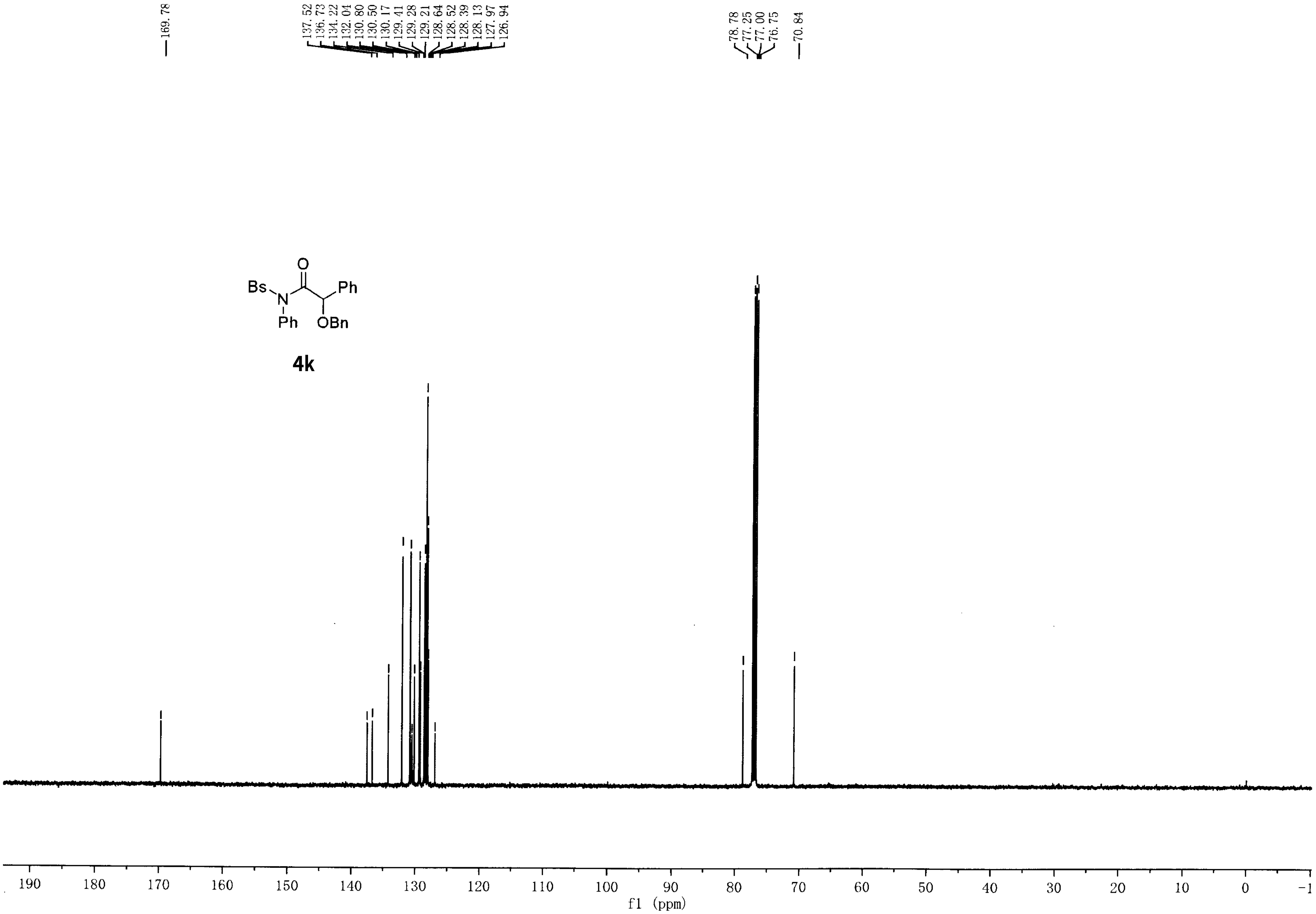
169.78
137.52
136.73
134.22
132.04
130.80
130.50
130.17
129.41
129.28
129.21
128.64
128.52
128.39
128.13
127.97
126.94
78.78
77.25
77.00
76.75
70.84
O
Bs
N
Ph
Ph
OBn
4k
190 180 170 160 150 140 130 120 110 100 90 80 70 60 50 40 30 20 10 0 -1
f1 (ppm)

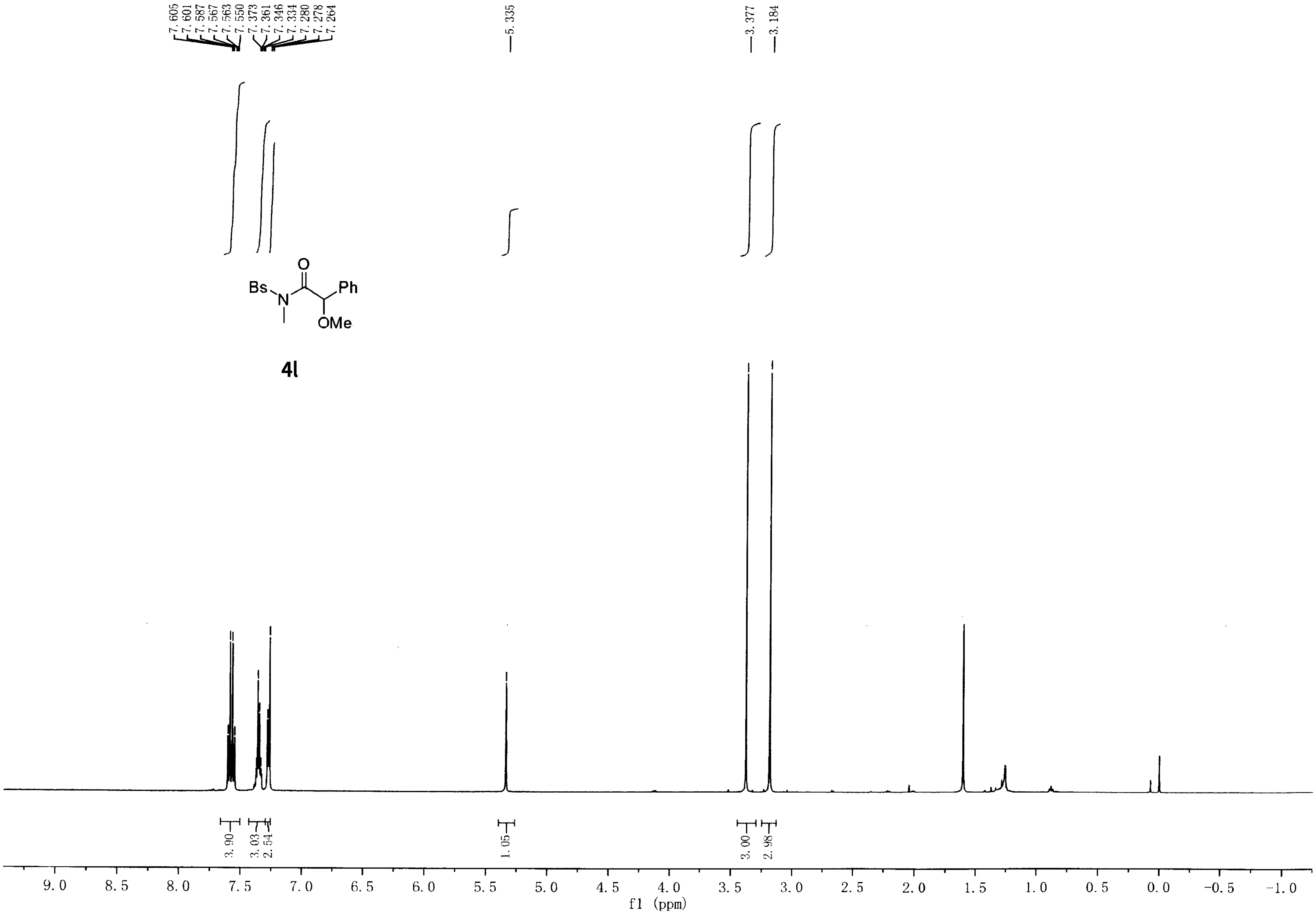
7.605
7.601
7.587
7.567
7.563
7.550
7.373
7.361
7.346
7.334
7.280
7.278
7.264
5.335
3.377
3.184
Bs
N
O
Ph
OMe
4l
3.90
3.03
2.54
1.05
3.00
2.98
9.0
8.5
8.0
7.5
7.0
6.5
6.0
5.5
5.0
4.5
4.0
3.5
3.0
2.5
2.0
1.5
1.0
0.5
0.0
-0.5
-1.0
f1 (ppm)

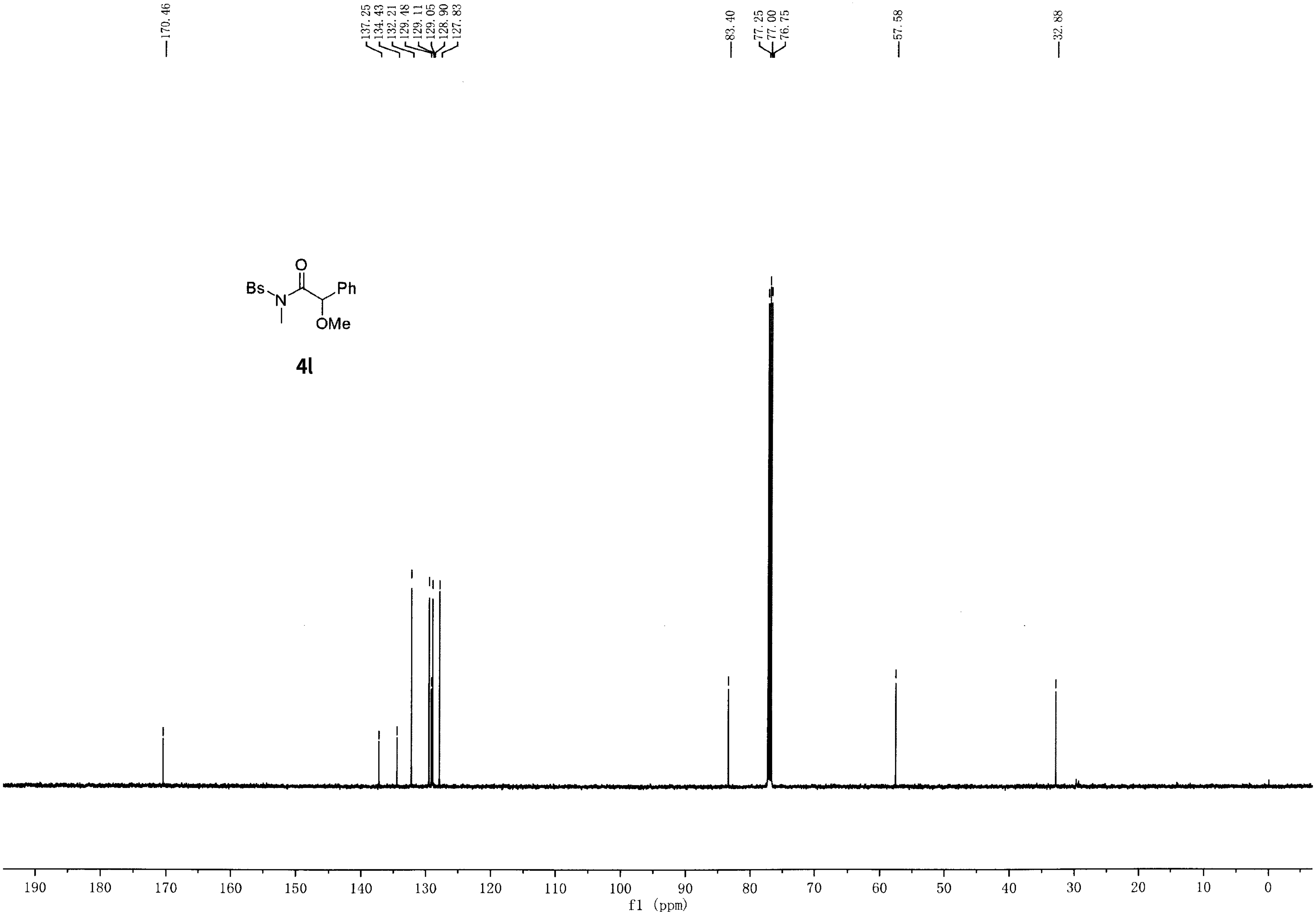
170.46
137.25
134.43
132.21
129.48
129.11
129.05
128.90
127.83
83.40
77.25
77.00
76.75
57.58
32.88
190 180 170 160 150 140 130 120 110 100 90 80 70 60 50 40 30 20 10 0
f1 (ppm)
O
Bs
N
Ph
OMe

4l

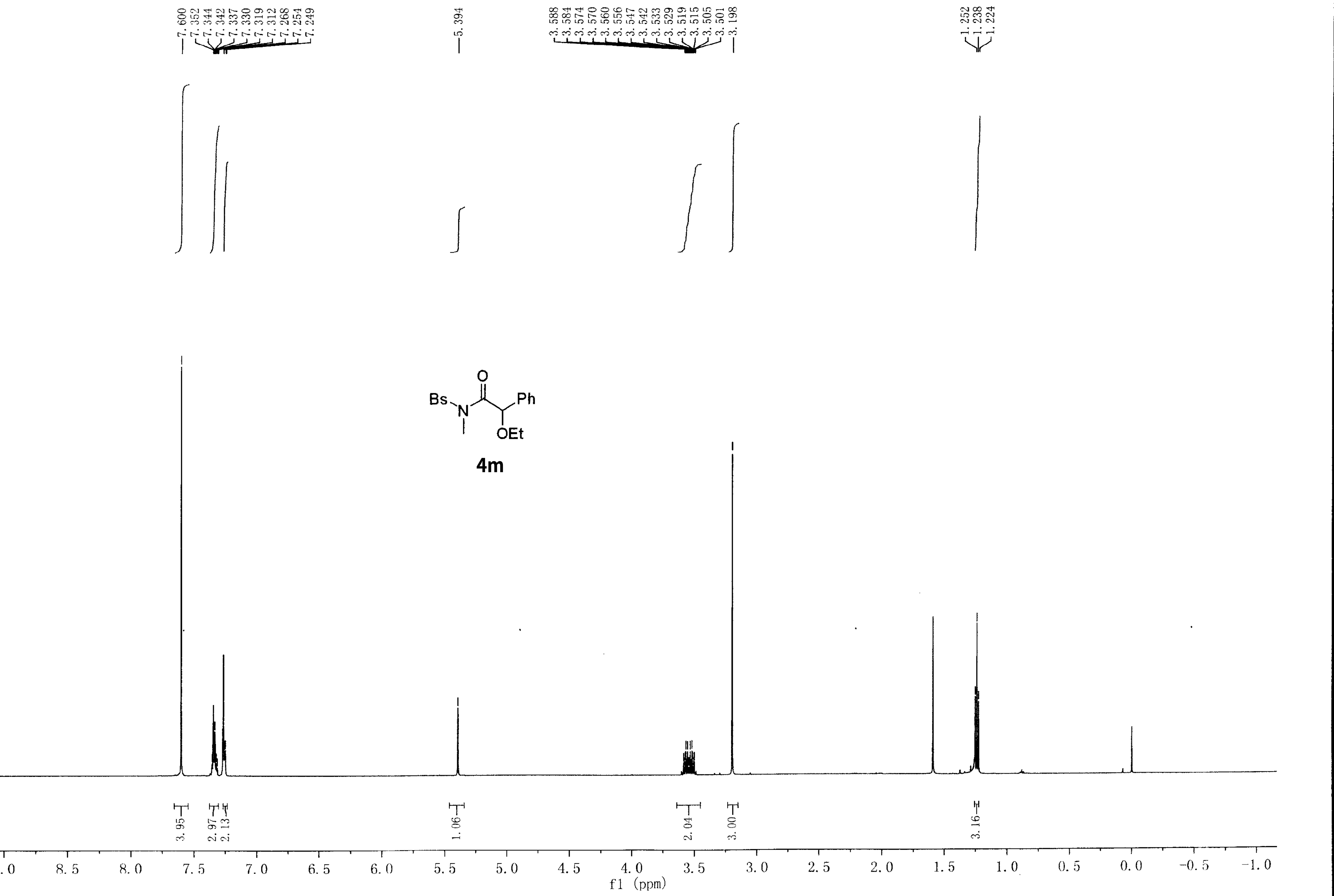

7.600
7.352
7.344
7.342
7.337
7.330
7.319
7.312
7.268
7.254
7.249
5.394
3.588
3.584
3.574
3.570
3.560
3.556
3.547
3.542
3.533
3.529
3.519
3.515
3.505
3.501
3.198
1.252
1.238
1.224
Bs
N
O
Ph
OEt
4m
3.95
2.97
2.13
1.06
2.04
3.00
3.16
f1 (ppm)

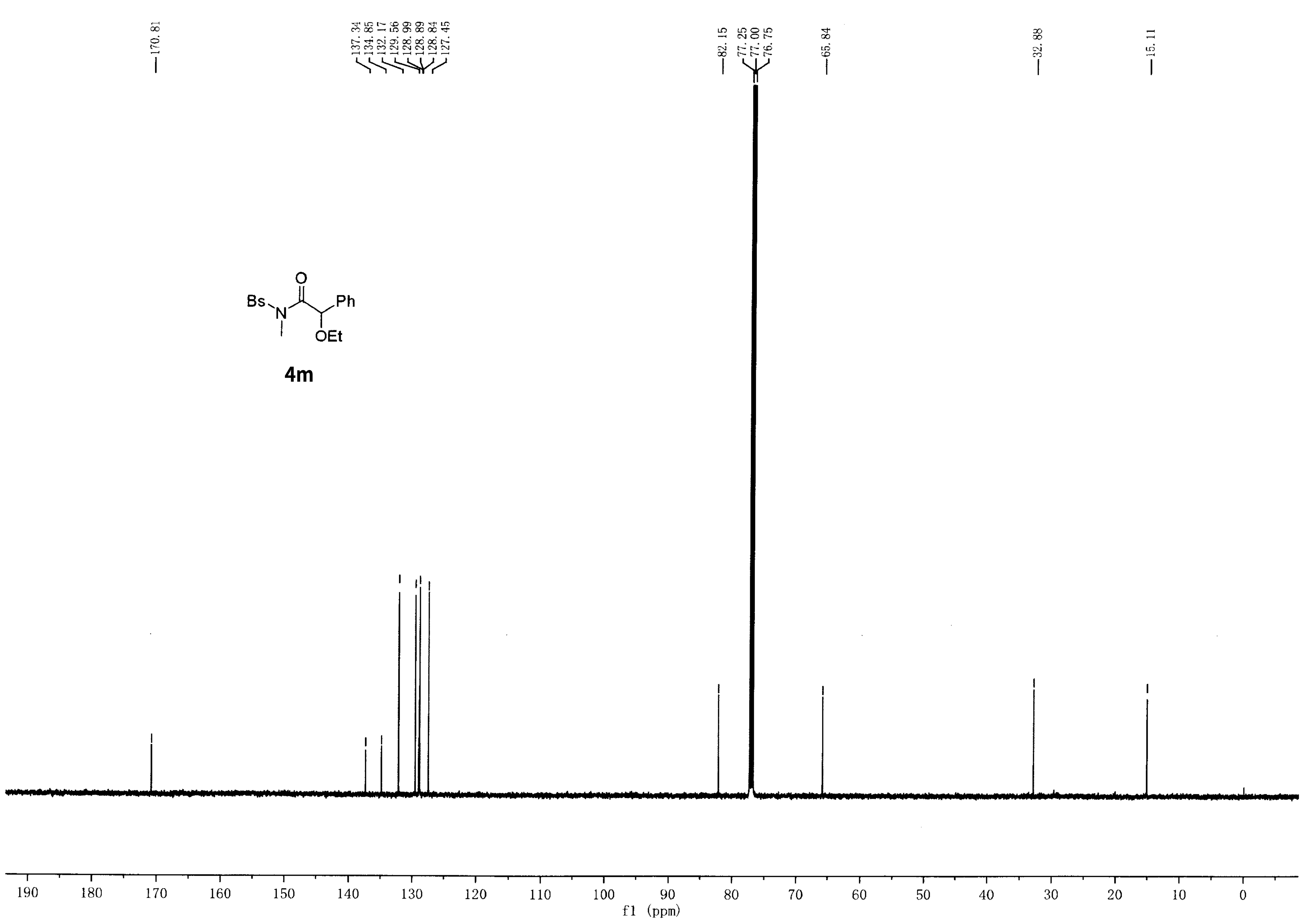

170.81
137.34
134.85
132.17
129.56
128.99
128.89
128.84
127.45
82.15
77.25
77.00
76.75
65.84
32.88
15.11
Bs
N
O
Ph
OEt
4m
190 180 170 160 150 140 130 120 110 100 90 80 70 60 50 40 30 20 10 0
f1 (ppm)

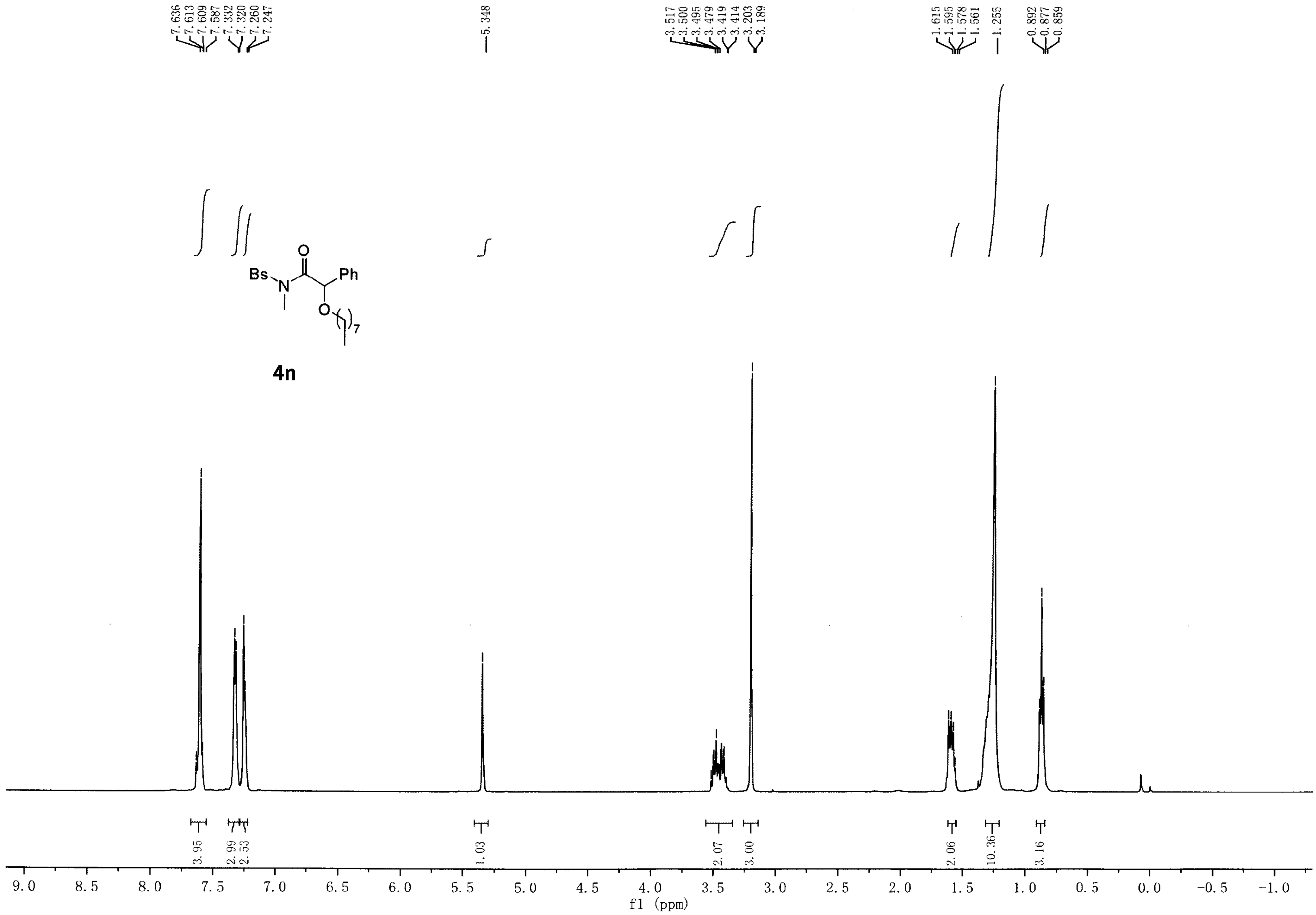

7.636
7.613
7.609
7.587
7.332
7.320
7.260
7.247
5.348
3.517
3.500
3.495
3.479
3.419
3.414
3.203
3.189
1.615
1.595
1.578
1.561
1.255
0.892
0.877
0.859
Bs
N
O
Ph
O
7
4n
3.95
2.99
2.53
1.03
2.07
3.00
2.06
10.36
3.16
9.0 8.5 8.0 7.5 7.0 6.5 6.0 5.5 5.0 4.5 4.0 3.5 3.0 2.5 2.0 1.5 1.0 0.5 0.0 -0.5 -1.0
f1 (ppm)

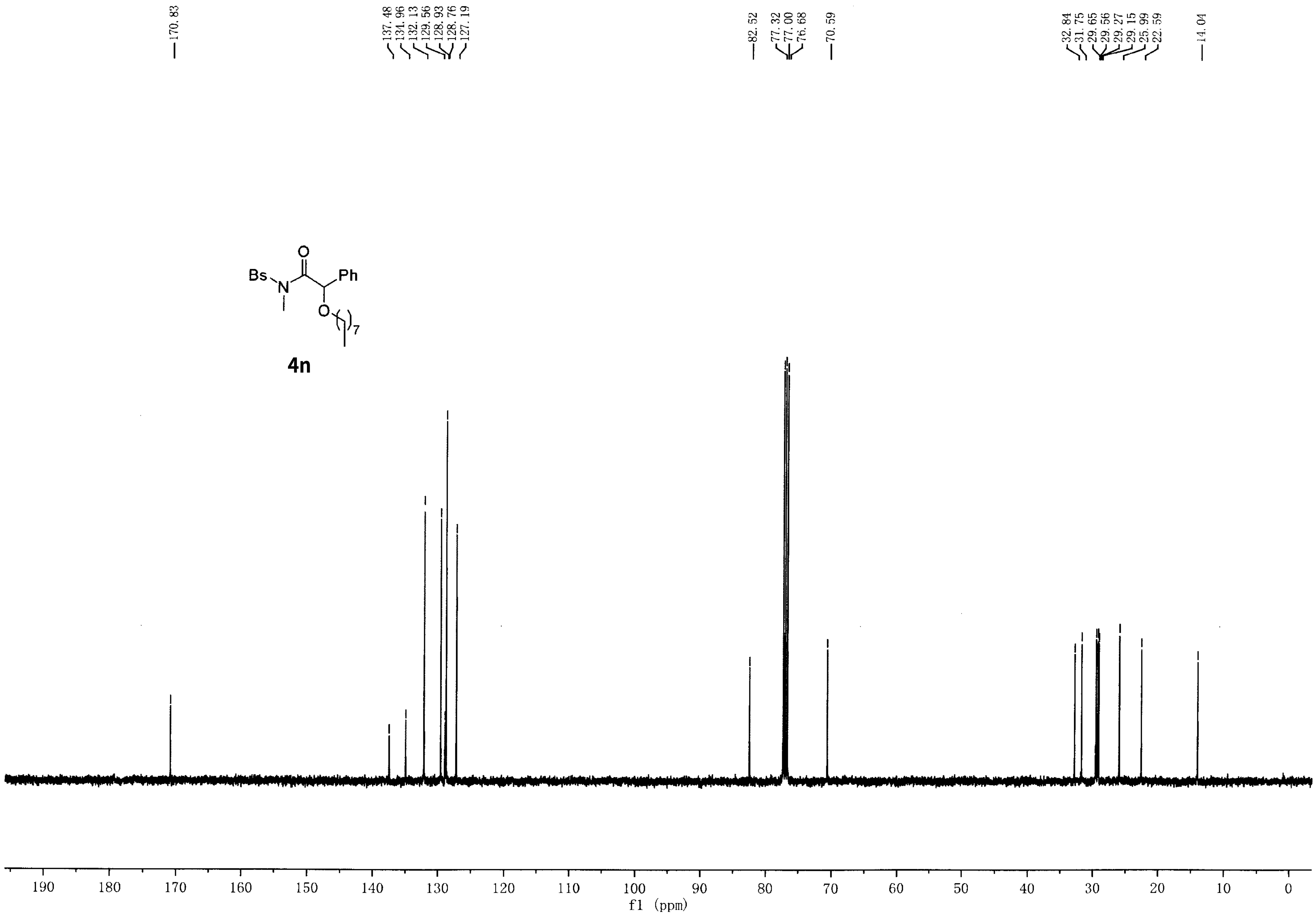
170.83
137.48
134.96
132.13
129.56
128.93
128.76
127.19
82.52
77.32
77.00
76.68
70.59
32.84
31.75
29.65
29.56
29.27
29.15
25.99
22.59
14.04
Bs
N
O
Ph
O
7
4n
190
180
170
160
150
140
130
120
110
100
90
80
70
60
50
40
30
20
10
0
f1 (ppm)

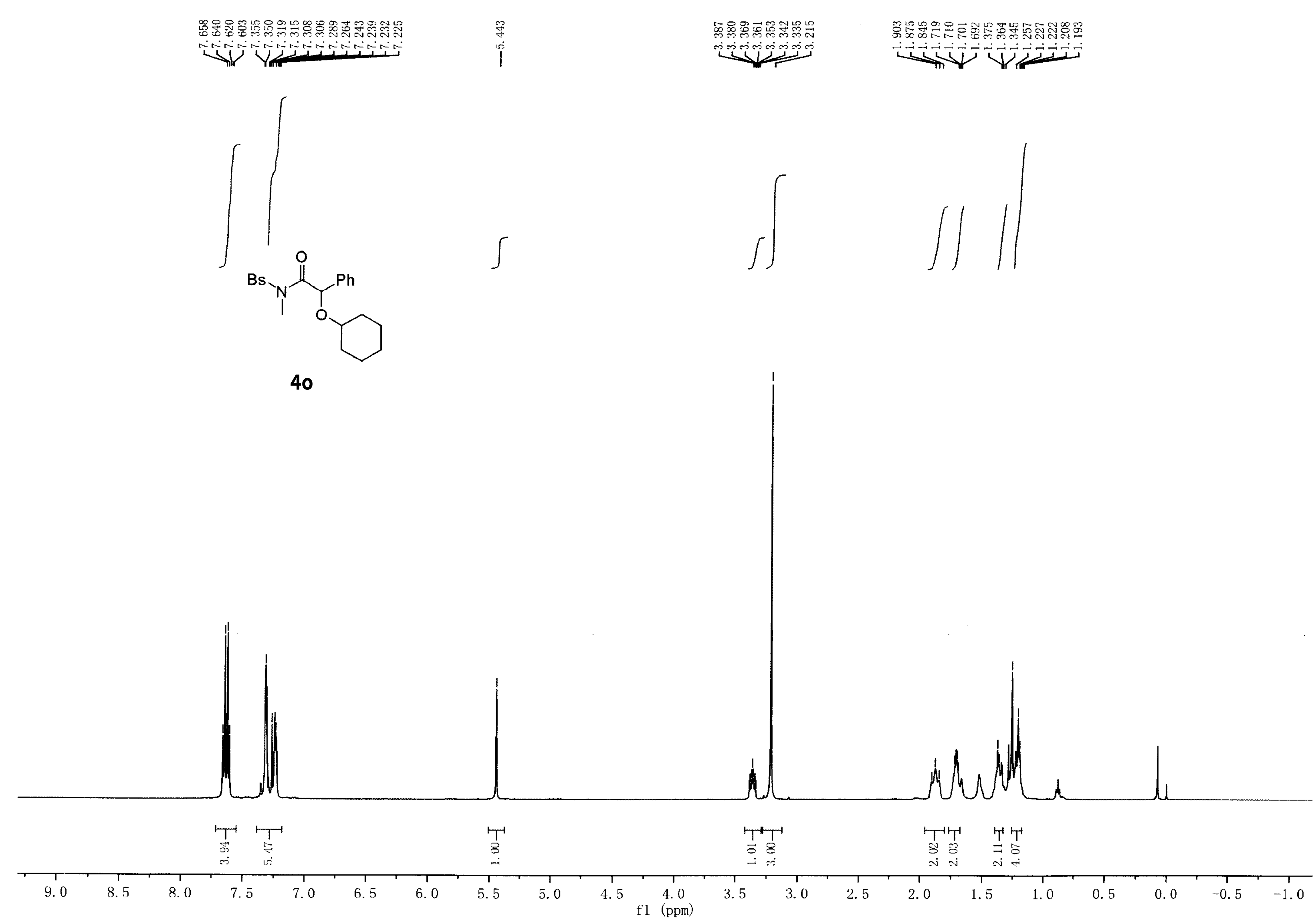
7.658
7.640
7.620
7.603
7.355
7.350
7.319
7.315
7.308
7.306
7.289
7.264
7.243
7.239
7.232
7.225
5.443
3.387
3.380
3.369
3.361
3.353
3.342
3.335
3.215
1.903
1.875
1.845
1.719
1.710
1.701
1.692
1.375
1.364
1.345
1.257
1.227
1.222
1.208
1.193
Bs
N
O
Ph
O
4o
3.94
5.47
1.00
1.01
3.00
2.02
2.03
2.11
4.07
9.0
8.5
8.0
7.5
7.0
6.5
6.0
5.5
5.0
4.5
4.0
3.5
3.0
2.5
2.0
1.5
1.0
0.5
0.0
-0.5
-1.0
f1 (ppm)

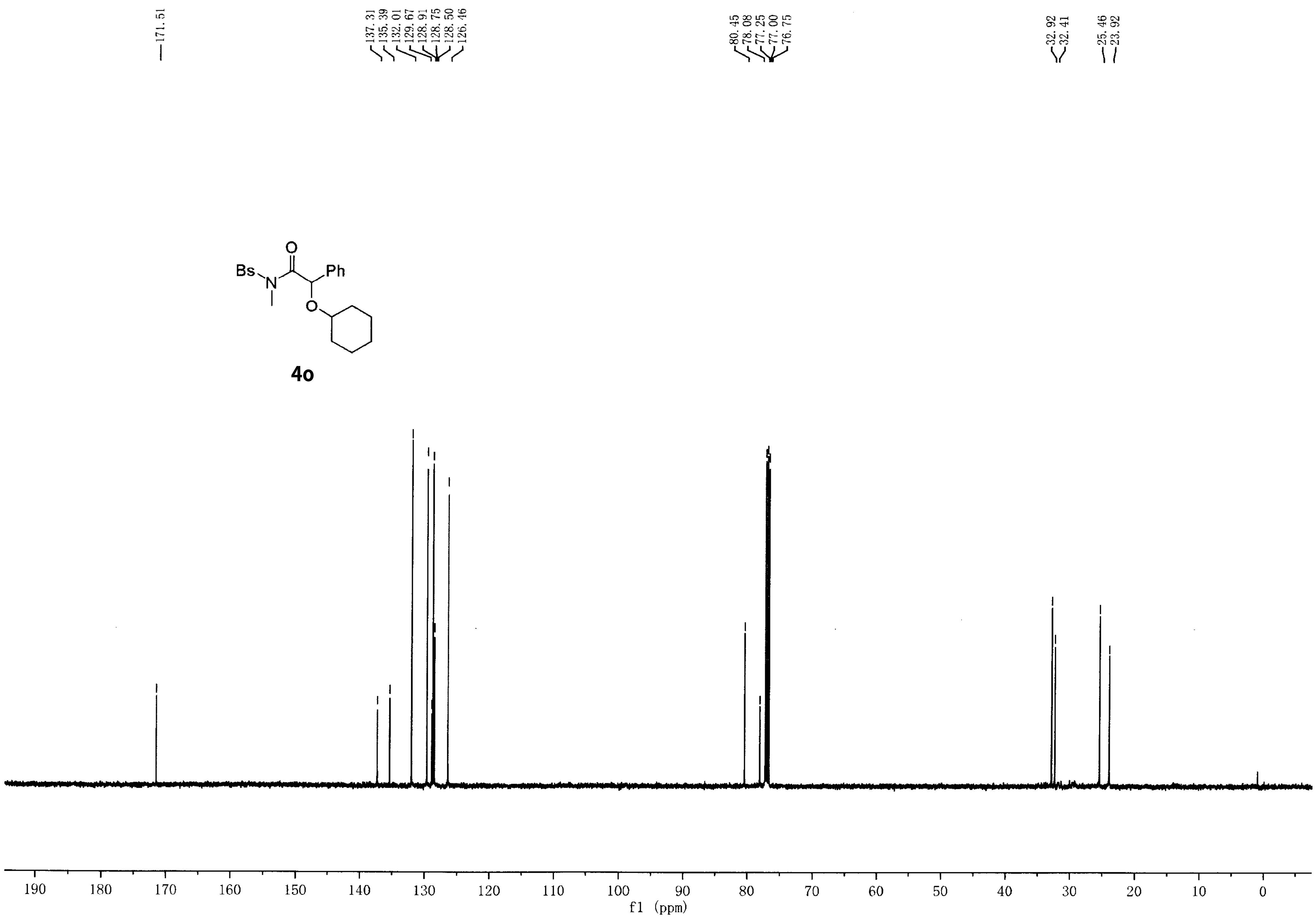
171.51
137.31
135.39
132.01
129.67
128.91
128.75
128.50
126.46
80.45
78.08
77.25
77.00
76.75
32.92
32.41
25.46
23.92
Bs
N
O
Ph
O
4o
190 180 170 160 150 140 130 120 110 100 90 80 70 60 50 40 30 20 10 0
f1 (ppm)

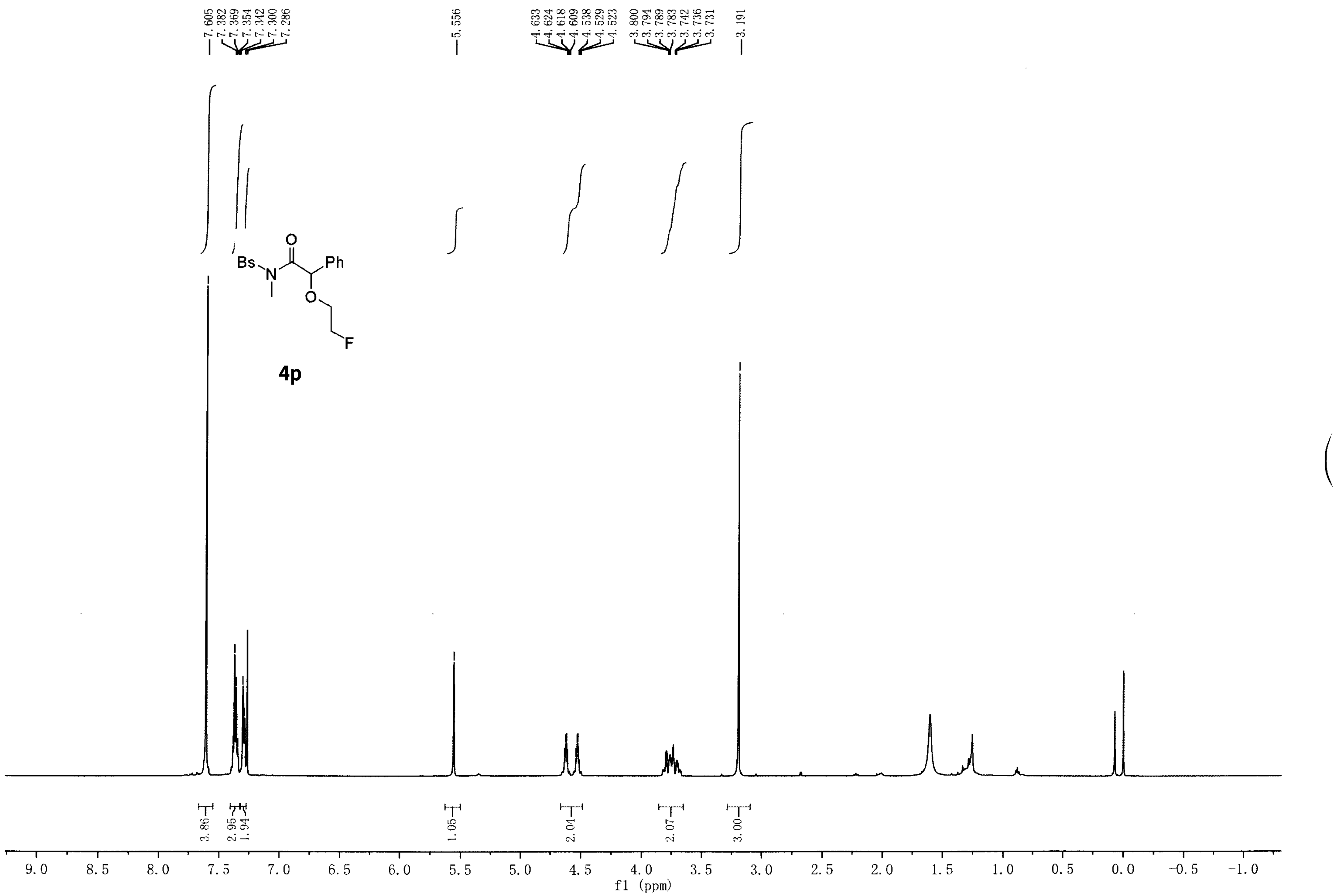
7.605
7.382
7.369
7.354
7.342
7.300
7.286
5.556
4.633
4.624
4.618
4.609
4.538
4.529
4.523
3.800
3.794
3.789
3.783
3.742
3.736
3.731
3.191
Bs
N
O
Ph
O
F
4p
3.86
2.95
1.94
1.05
2.01
2.07
3.00
9.0
8.5
8.0
7.5
7.0
6.5
6.0
5.5
5.0
4.5
4.0
3.5
3.0
2.5
2.0
1.5
1.0
0.5
0.0
-0.5
-1.0
f1 (ppm)

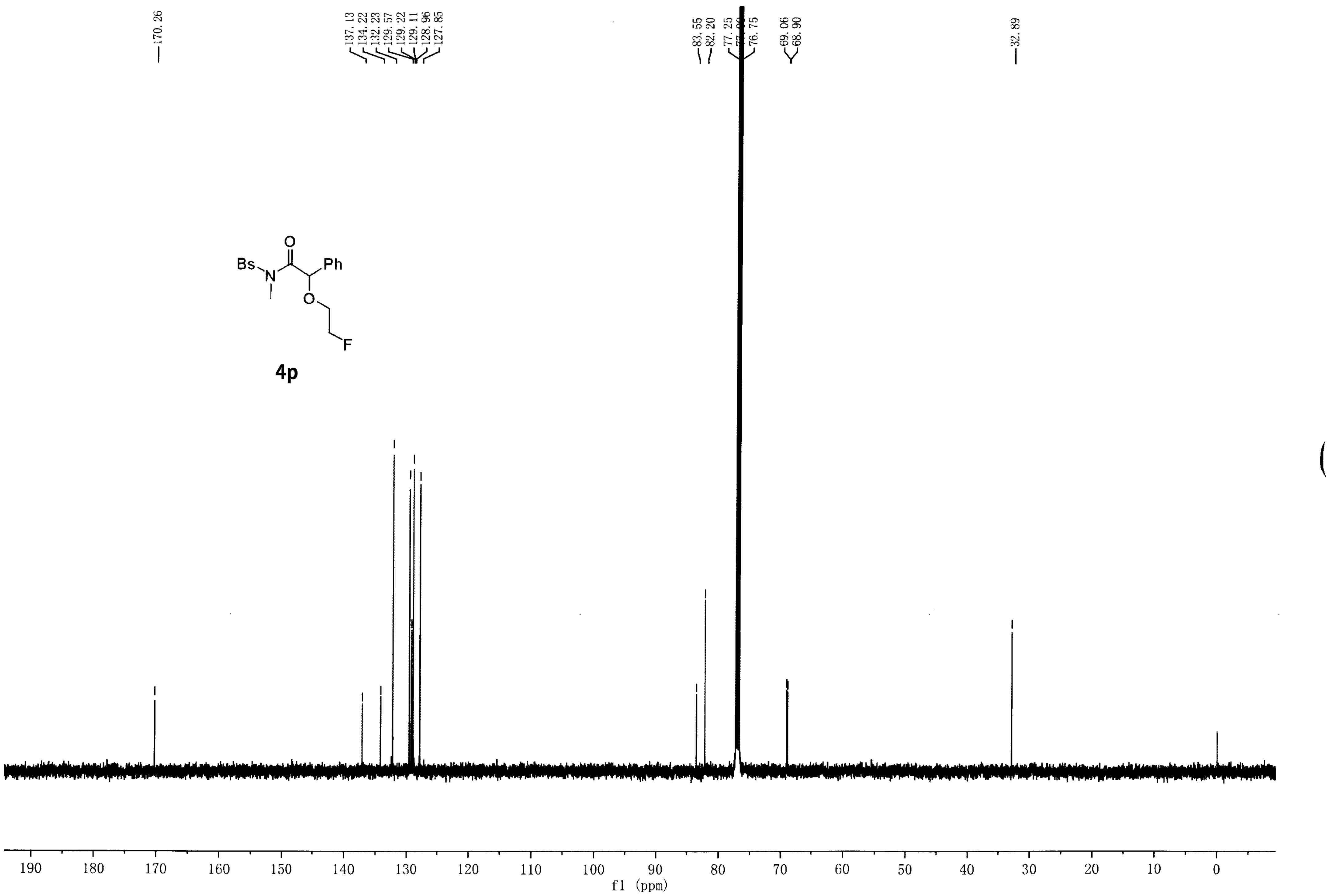

170.26
137.13
134.22
132.23
129.57
129.22
129.11
128.96
127.85
83.55
82.20
77.25
76.75
69.06
68.90
32.89
Bs
N
O
Ph
O
F
4p
190 180 170 160 150 140 130 120 110 100 90 80 70 60 50 40 30 20 10 0
f1 (ppm)

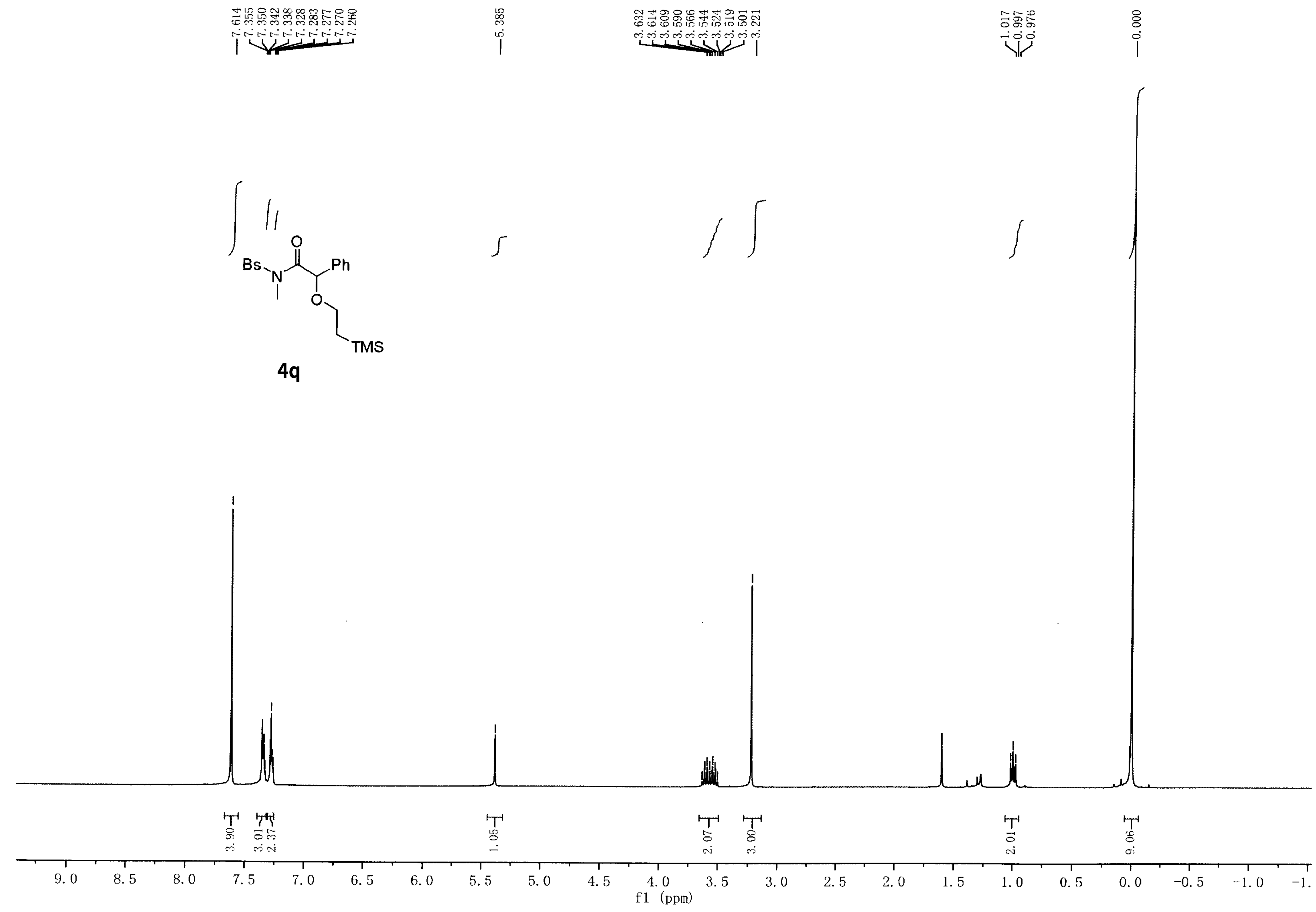
7.614
7.355
7.350
7.342
7.338
7.328
7.283
7.277
7.270
7.260
5.385
3.632
3.614
3.609
3.590
3.566
3.544
3.524
3.519
3.501
3.221
1.017
0.997
0.976
0.000
Bs
N
O
Ph
O
TMS
4q
3.90
3.01
2.37
1.05
2.07
3.00
2.01
9.06
9.0
8.5
8.0
7.5
7.0
6.5
6.0
5.5
5.0
4.5
4.0
3.5
3.0
2.5
2.0
1.5
1.0
0.5
0.0
-0.5
-1.0
f1 (ppm)

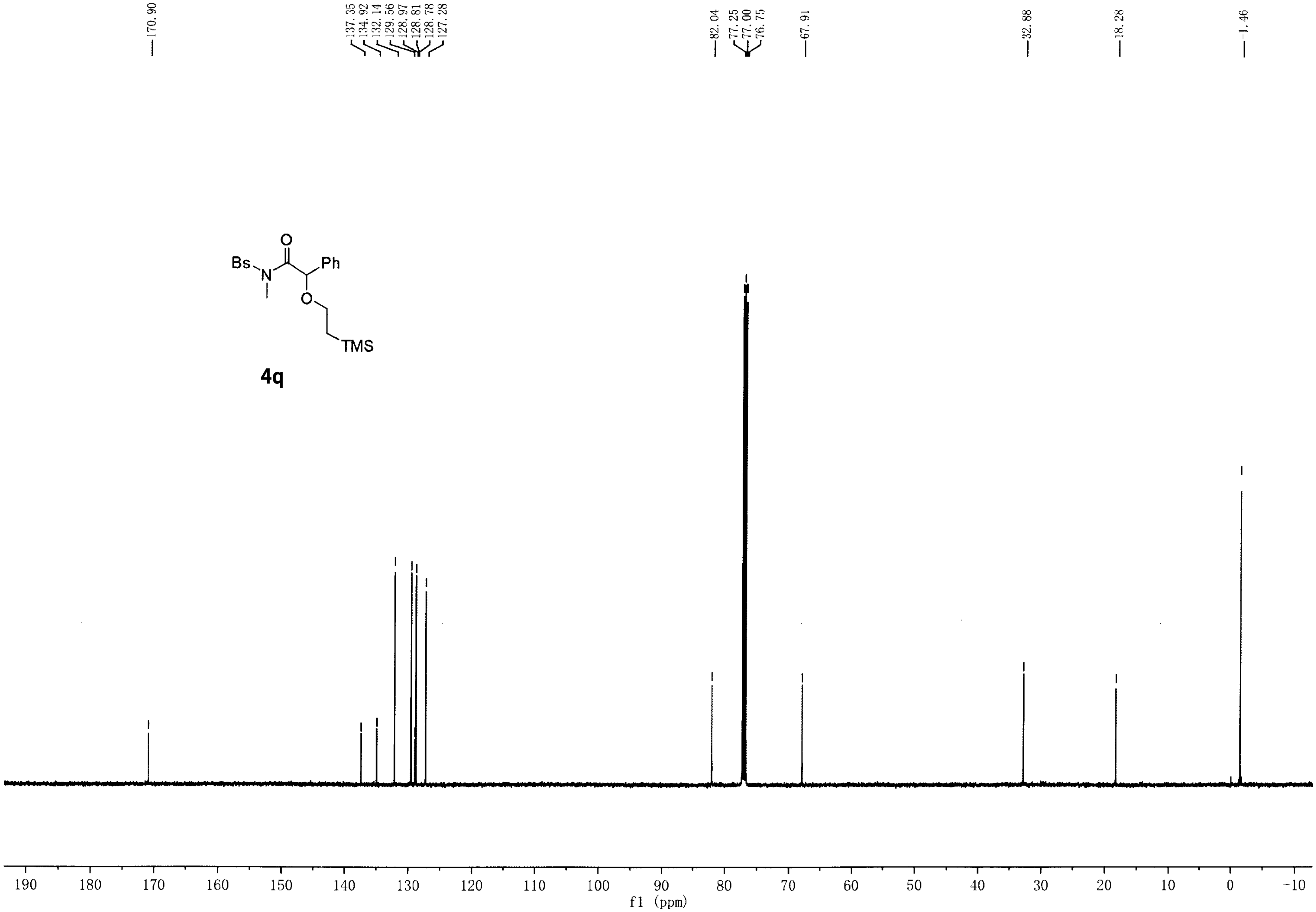
170.90
137.35
134.92
132.14
129.56
128.97
128.81
128.78
127.28
82.04
77.25
77.00
76.75
67.91
32.88
18.28
-1.46
Bs
N
O
Ph
O
TMS
4q
190 180 170 160 150 140 130 120 110 100 90 80 70 60 50 40 30 20 10 0 -10
f1 (ppm)

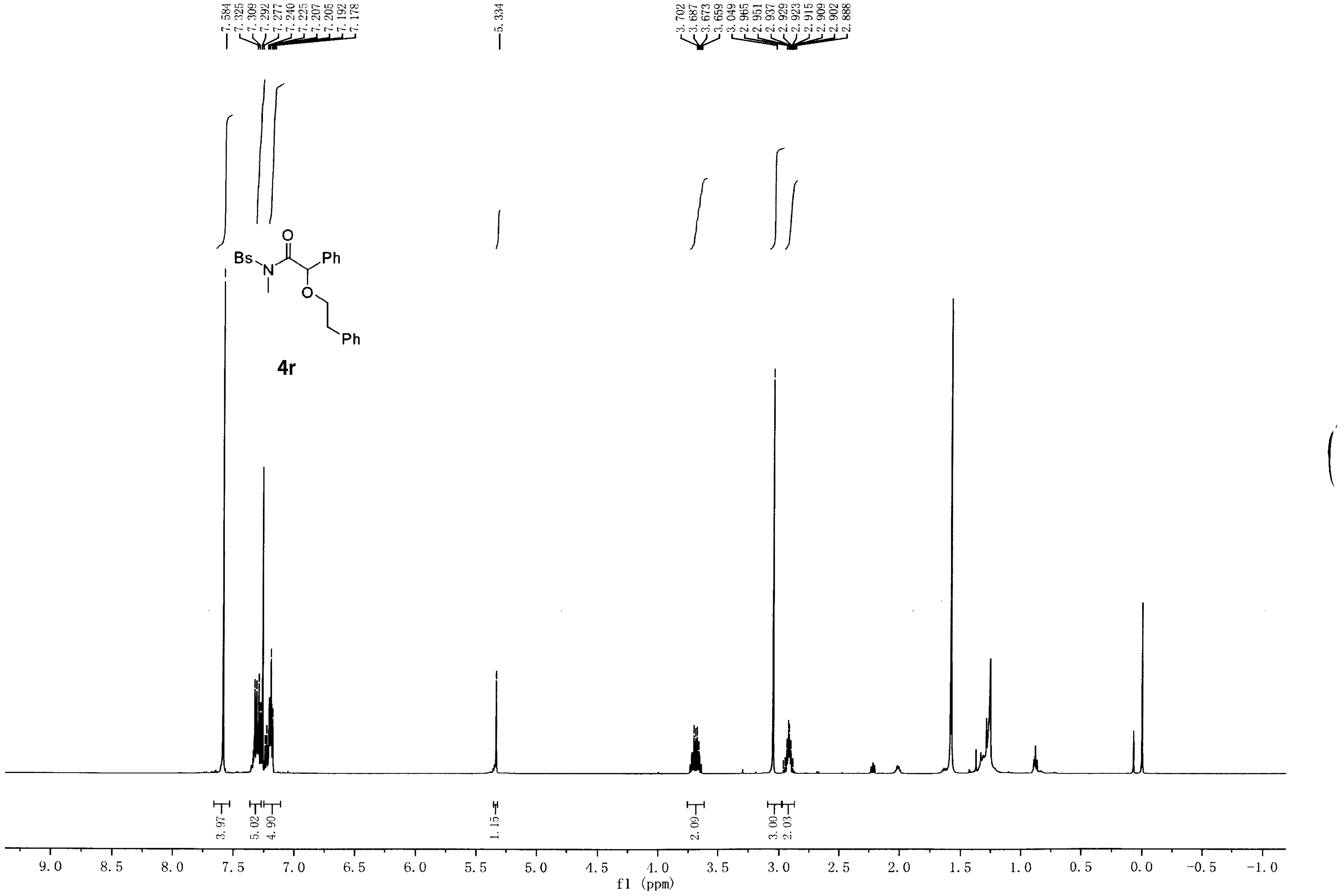
7.584
7.325
7.309
7.292
7.277
7.240
7.225
7.207
7.205
7.192
7.178
5.334
3.702
3.687
3.673
3.659
3.049
2.965
2.951
2.937
2.929
2.923
2.915
2.909
2.902
2.888
Bs
N
O
Ph
O
Ph
4r
3.97
5.02
4.90
1.15
2.09
3.00
2.03
9.0
8.5
8.0
7.5
7.0
6.5
6.0
5.5
5.0
4.5
4.0
3.5
3.0
2.5
2.0
1.5
1.0
0.5
0.0
-0.5
-1.0
f1 (ppm)

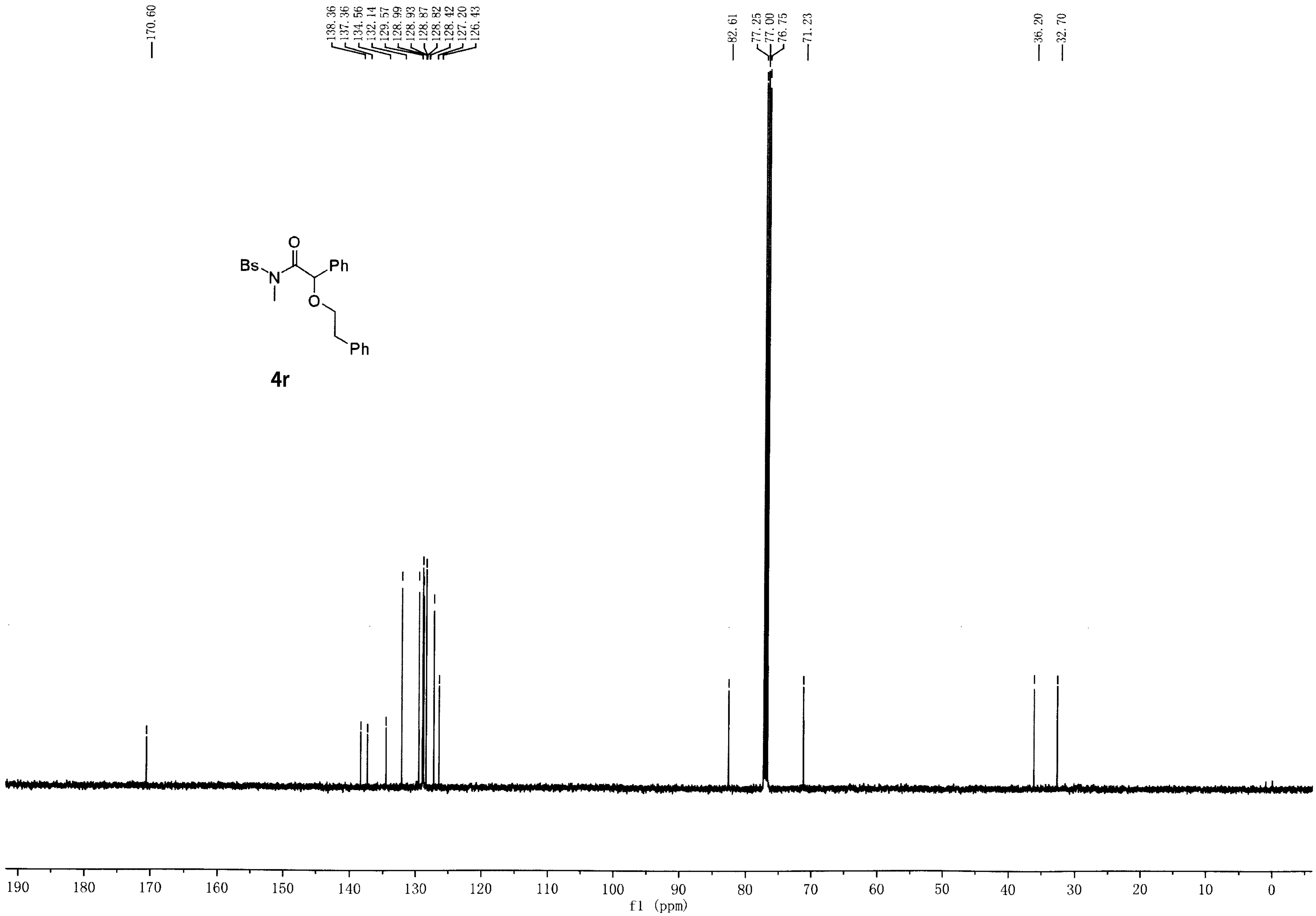
170.60
138.36
137.36
134.56
132.14
129.57
128.99
128.93
128.87
128.82
128.42
127.20
126.43
82.61
77.25
77.00
76.75
71.23
36.20
32.70
Bs
N
O
Ph
O
Ph
4r
190 180 170 160 150 140 130 120 110 100 90 80 70 60 50 40 30 20 10 0
f1 (ppm)

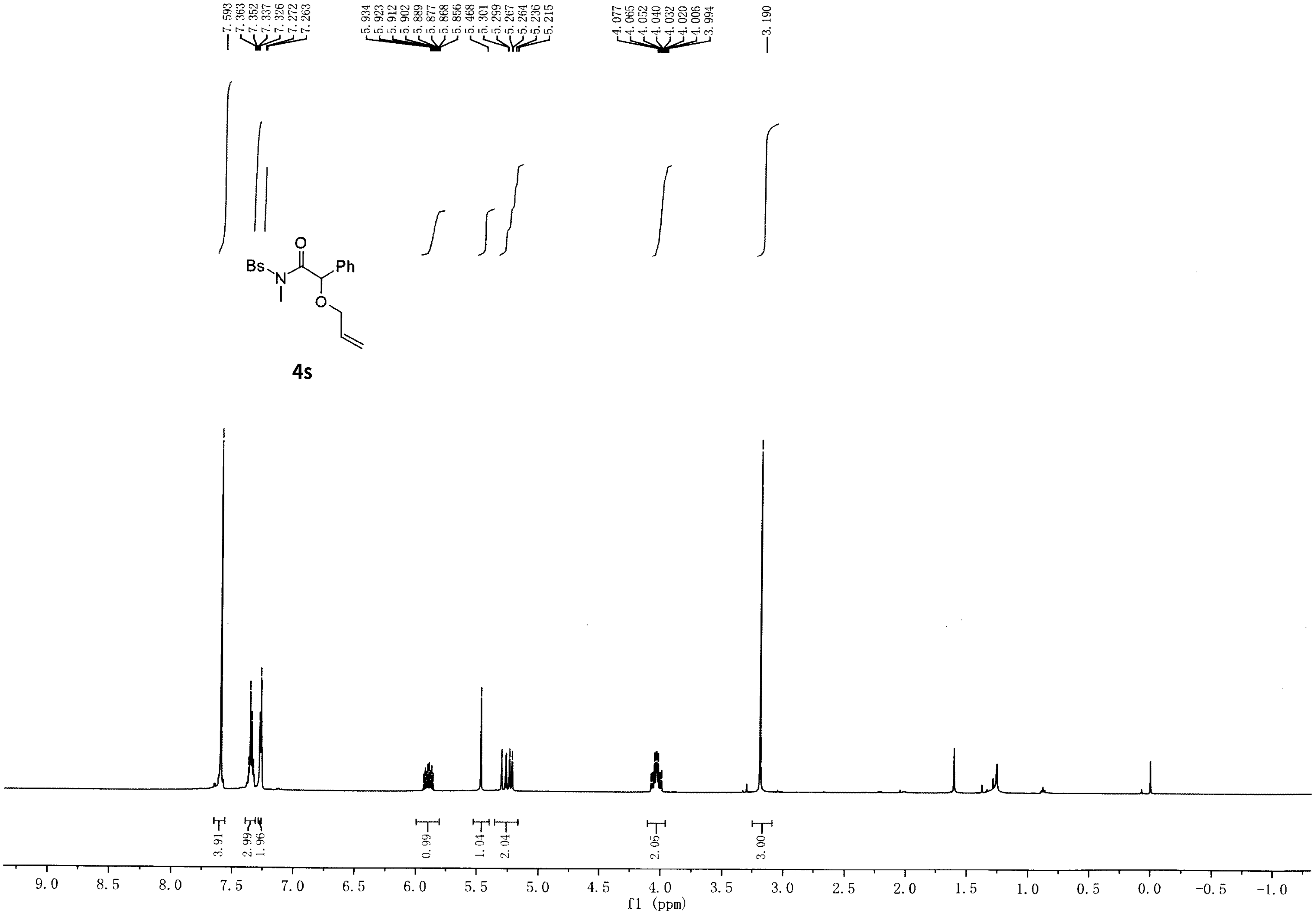
7.593
7.363
7.352
7.337
7.326
7.272
7.263
5.934
5.923
5.912
5.902
5.889
5.877
5.868
5.856
5.468
5.301
5.299
5.267
5.264
5.236
5.215
4.077
4.065
4.052
4.040
4.032
4.020
4.006
3.994
3.190
Bs
N
O
Ph
O
4s
3.91
2.99
1.96
0.99
1.04
2.04
2.05
3.00
9.0 8.5 8.0 7.5 7.0 6.5 6.0 5.5 5.0 4.5 4.0 3.5 3.0 2.5 2.0 1.5 1.0 0.5 0.0 -0.5 -1.0
f1 (ppm)

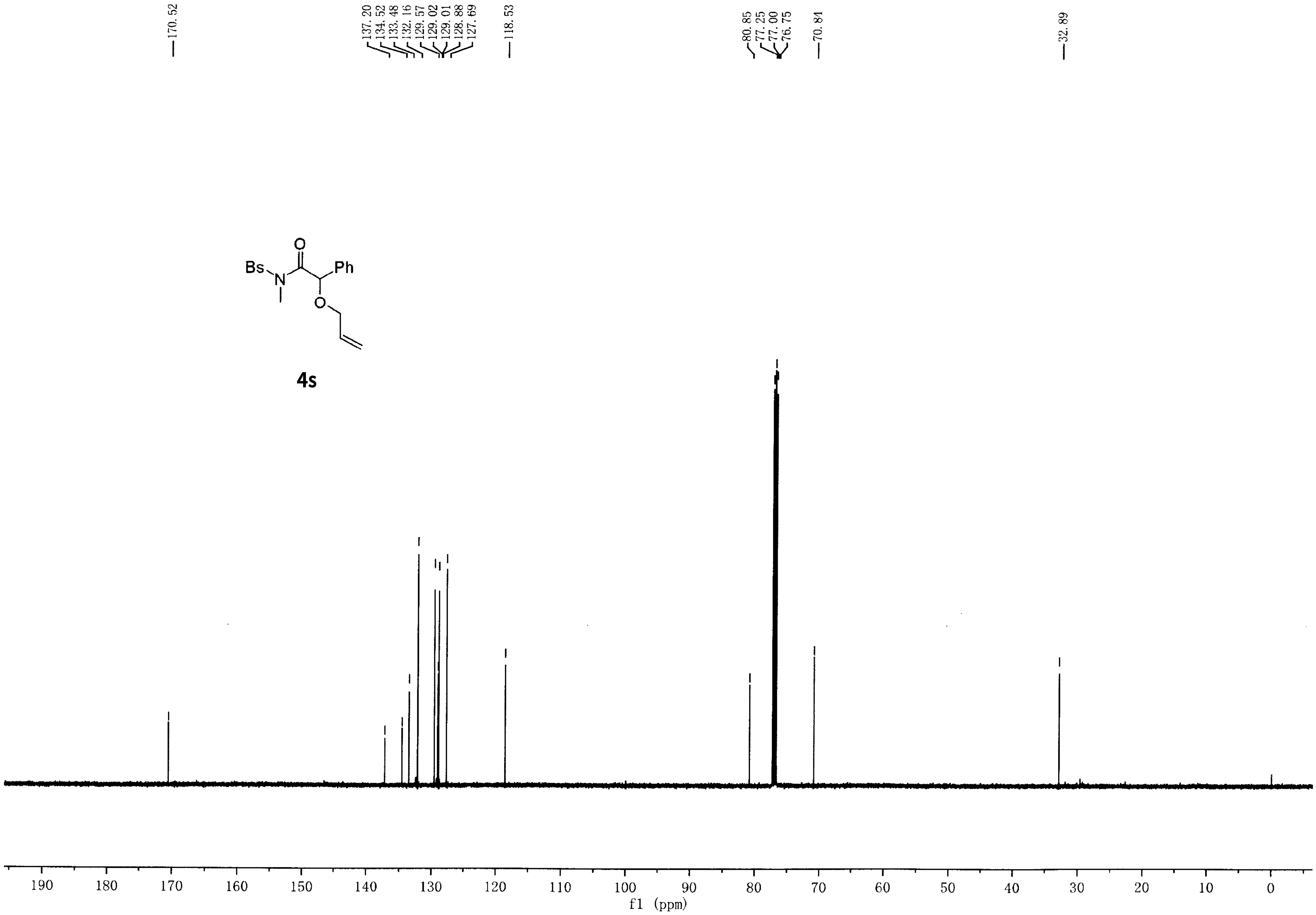
170.52
137.20
134.52
133.48
132.16
129.57
129.02
129.01
128.88
127.69
118.53
80.85
77.25
77.00
76.75
70.84
32.89
Bs
N
O
Ph
O
4s
190 180 170 160 150 140 130 120 110 100 90 80 70 60 50 40 30 20 10 0
f1 (ppm)

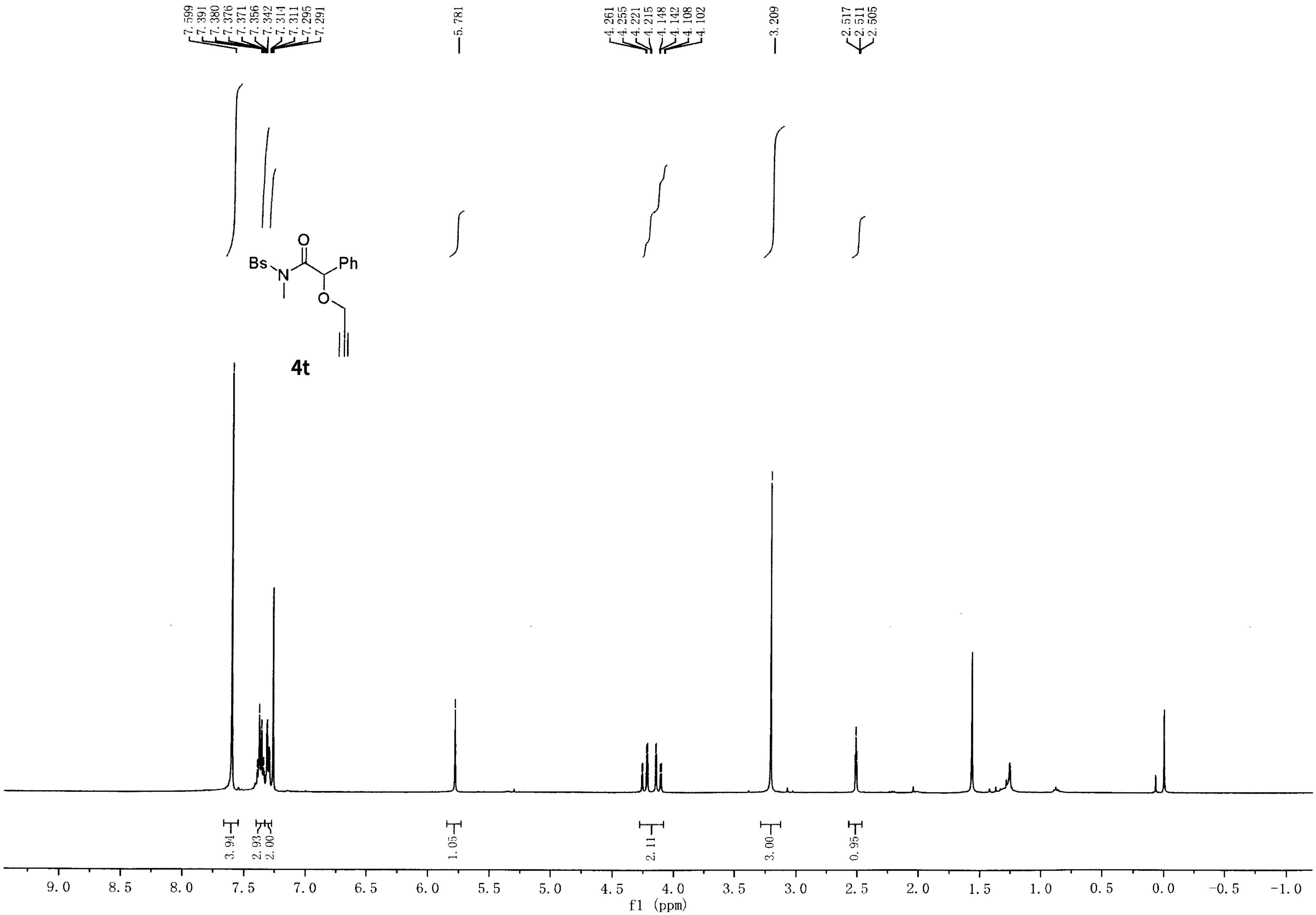

7.599
7.391
7.380
7.376
7.371
7.356
7.342
7.314
7.311
7.295
7.291
5.781
4.261
4.255
4.221
4.215
4.148
4.142
4.108
4.102
3.209
2.517
2.511
2.505
Bs
N
O
Ph
O
4t
3.91
2.93
2.00
1.05
2.11
3.00
0.95
9.0
8.5
8.0
7.5
7.0
6.5
6.0
5.5
5.0
4.5
4.0
3.5
3.0
2.5
2.0
1.5
1.0
0.5
0.0
-0.5
-1.0
f1 (ppm)

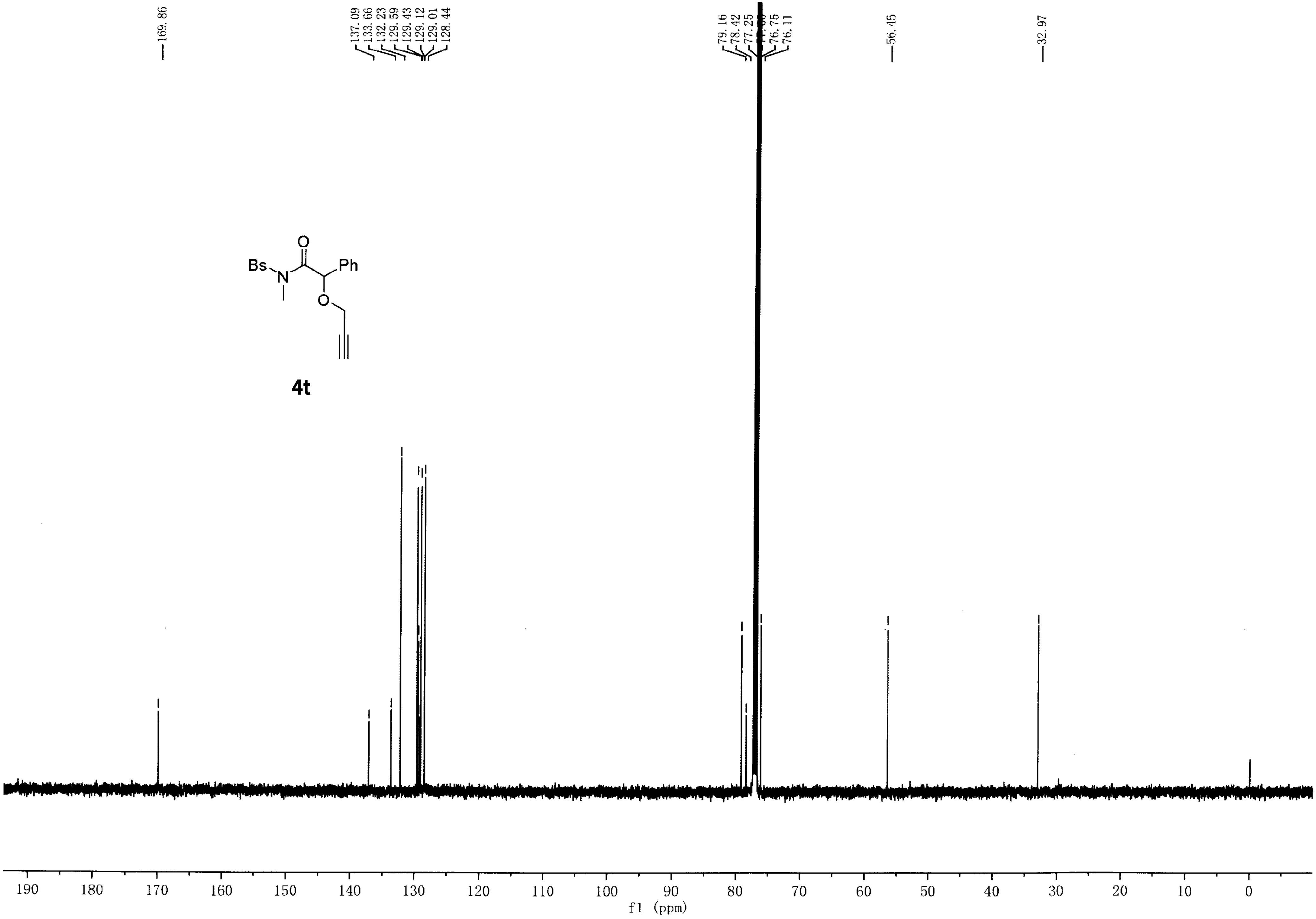
169.86
137.09
133.66
132.23
129.59
129.43
129.12
129.01
128.44
79.16
78.42
77.25
76.75
76.11
56.45
32.97
190 180 170 160 150 140 130 120 110 100 90 80 70 60 50 40 30 20 10 0
f1 (ppm)
Bs
N
O
Ph
O

4t

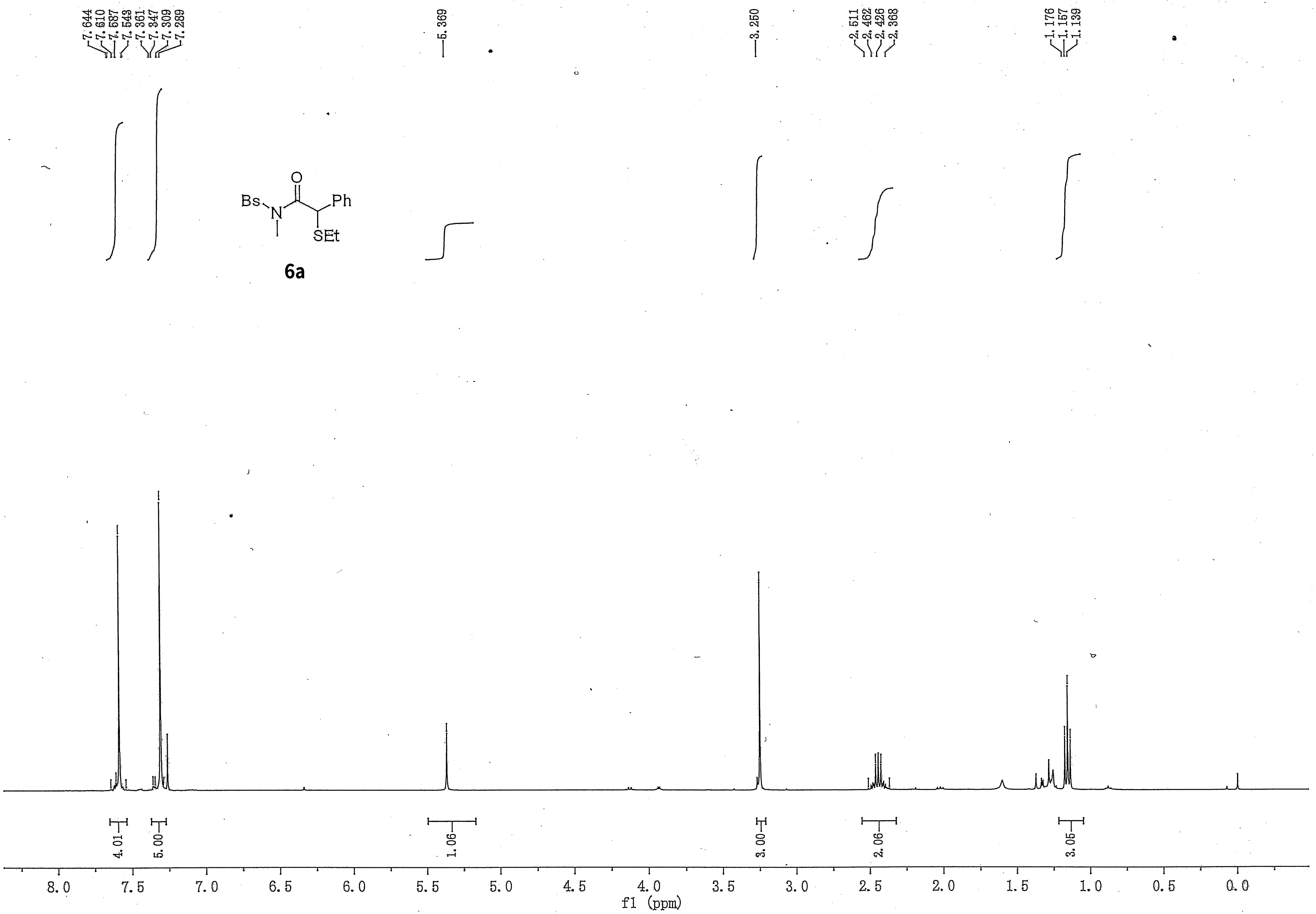
7.644
7.610
7.587
7.543
7.361
7.347
7.309
7.289
5.369
3.250
2.511
2.462
2.426
2.368
1.176
1.157
1.139
O
Bs
N
Ph
SEt
6a
4.01
5.00
1.06
3.00
2.06
3.05
8.0 7.5 7.0 6.5 6.0 5.5 5.0 4.5 4.0 3.5 3.0 2.5 2.0 1.5 1.0 0.5 0.0
f1 (ppm)

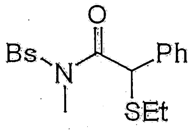
Bs
N
O
Ph
SEt

6a

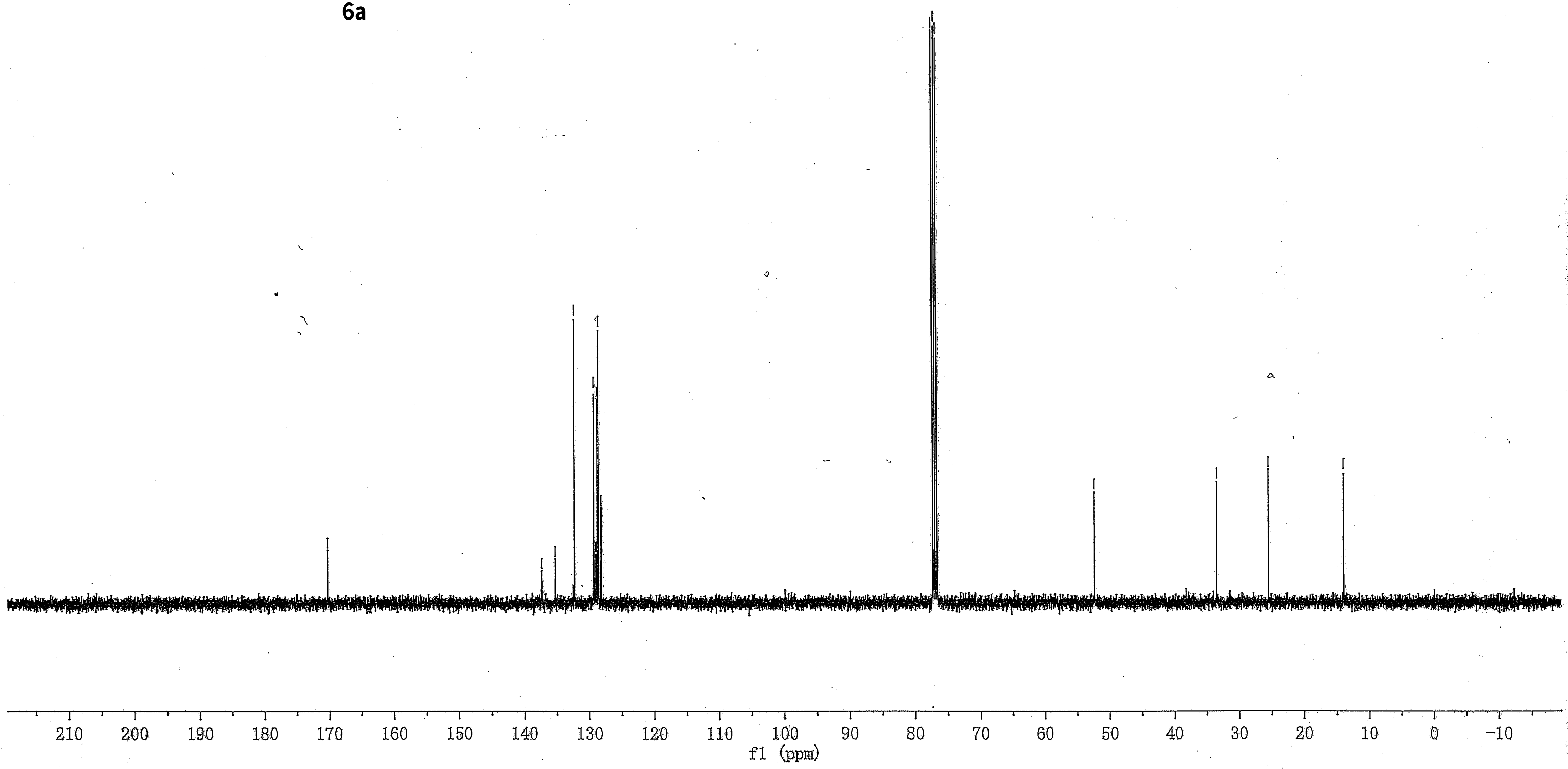
170.28
137.35
135.32
132.31
129.31
129.04
128.84
128.61
128.22
77.32
77.00
76.68
52.38
33.50
25.51
13.97
210 200 190 180 170 160 150 140 130 120 110 100 90 80 70 60 50 40 30 20 10 0 −10
f1 (ppm)

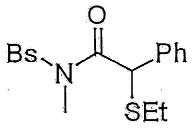
O
Bs
N
Ph
SEt

6a

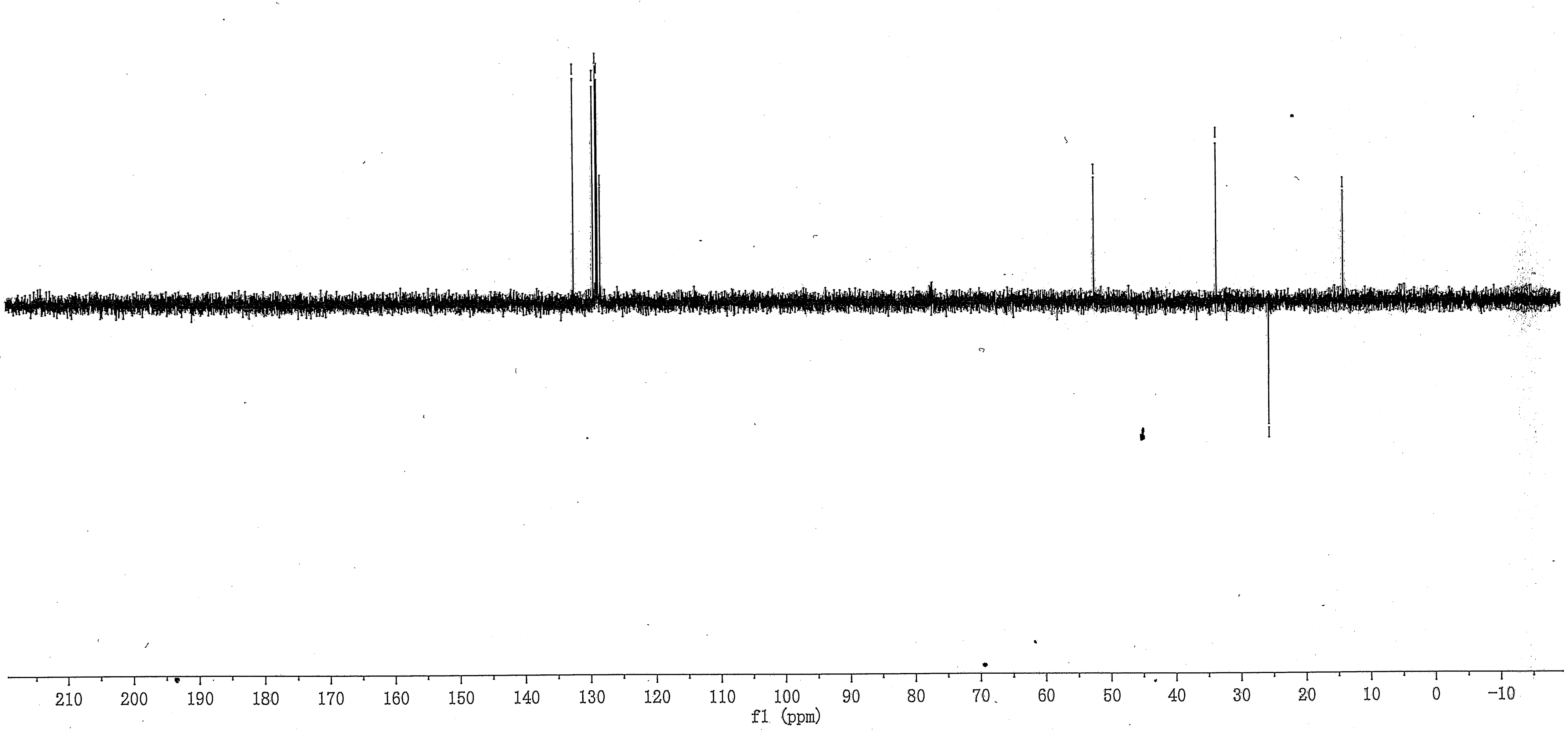
132.31
129.32
128.84
128.62
128.22
52.38
33.50
25.51
13.97
210 200 190 180 170 160 150 140 130 120 110 100 90 80 70 60 50 40 30 20 10 0 -10
f1 (ppm)

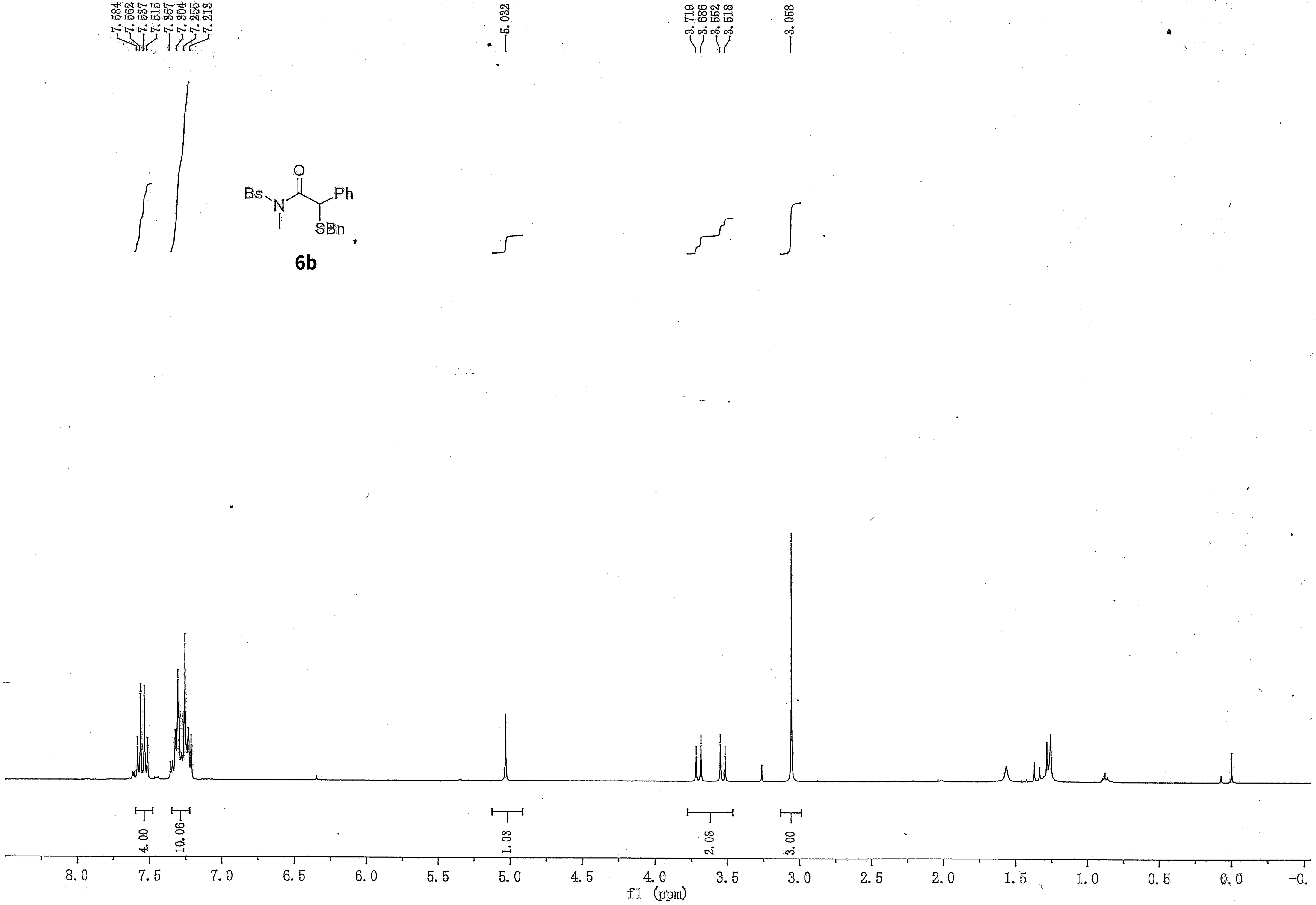
7.584
7.562
7.537
7.515
7.357
7.304
7.255
7.213
5.032
3.719
3.686
3.552
3.518
3.058
Bs
N
O
Ph
SBn
6b
4.00
10.06
1.03
2.08
3.00
8.0
7.5
7.0
6.5
6.0
5.5
5.0
4.5
4.0
3.5
3.0
2.5
2.0
1.5
1.0
0.5
0.0
-0.
f1 (ppm)

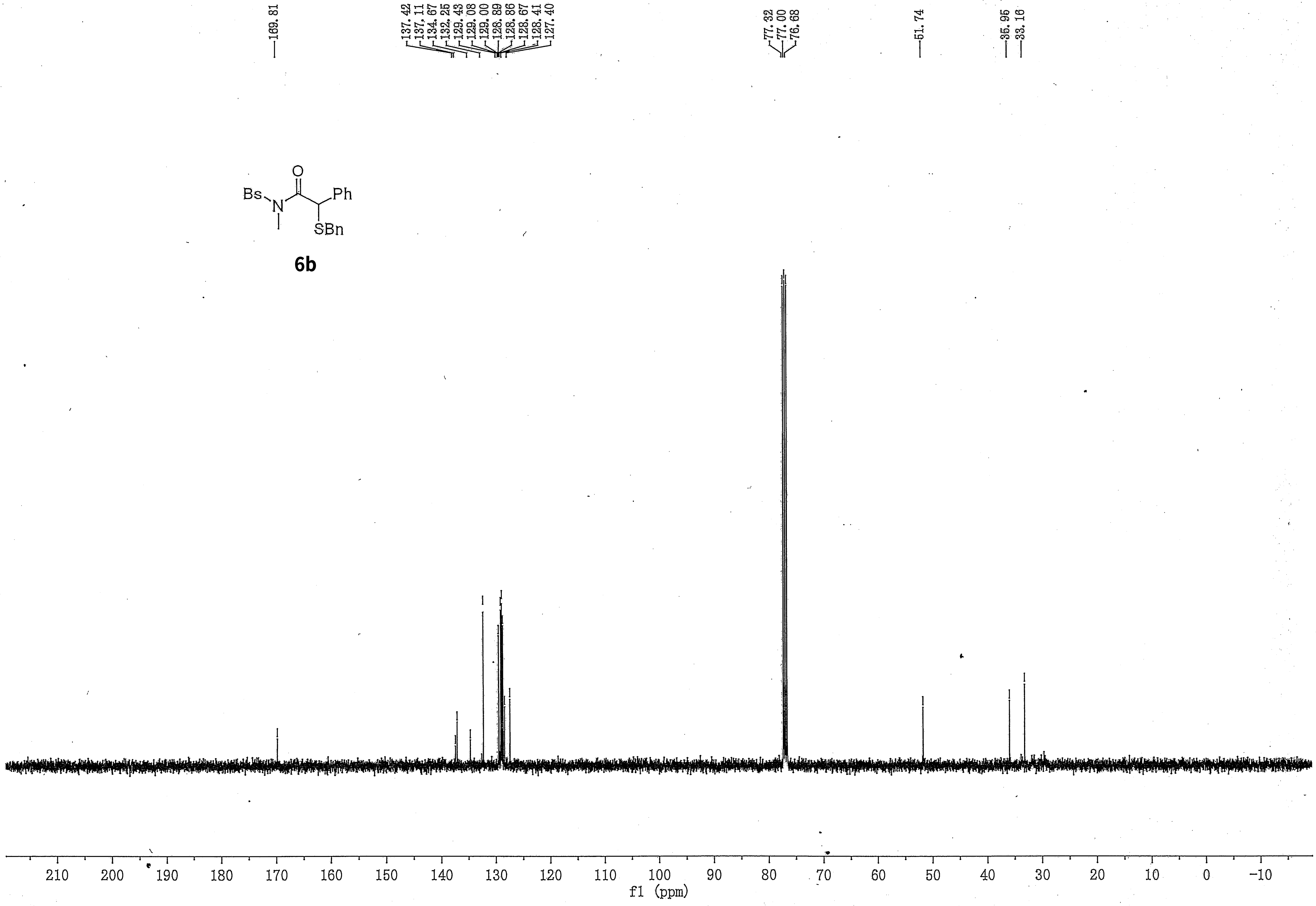
169.81
137.42
137.11
134.67
132.25
129.43
129.08
129.00
128.89
128.86
128.67
128.41
127.40
77.32
77.00
76.68
51.74
35.95
33.16
210 200 190 180 170 160 150 140 130 120 110 100 90 80 70 60 50 40 30 20 10 0 -10
f1 (ppm)
O
Bs
N
Ph
SBn

6b

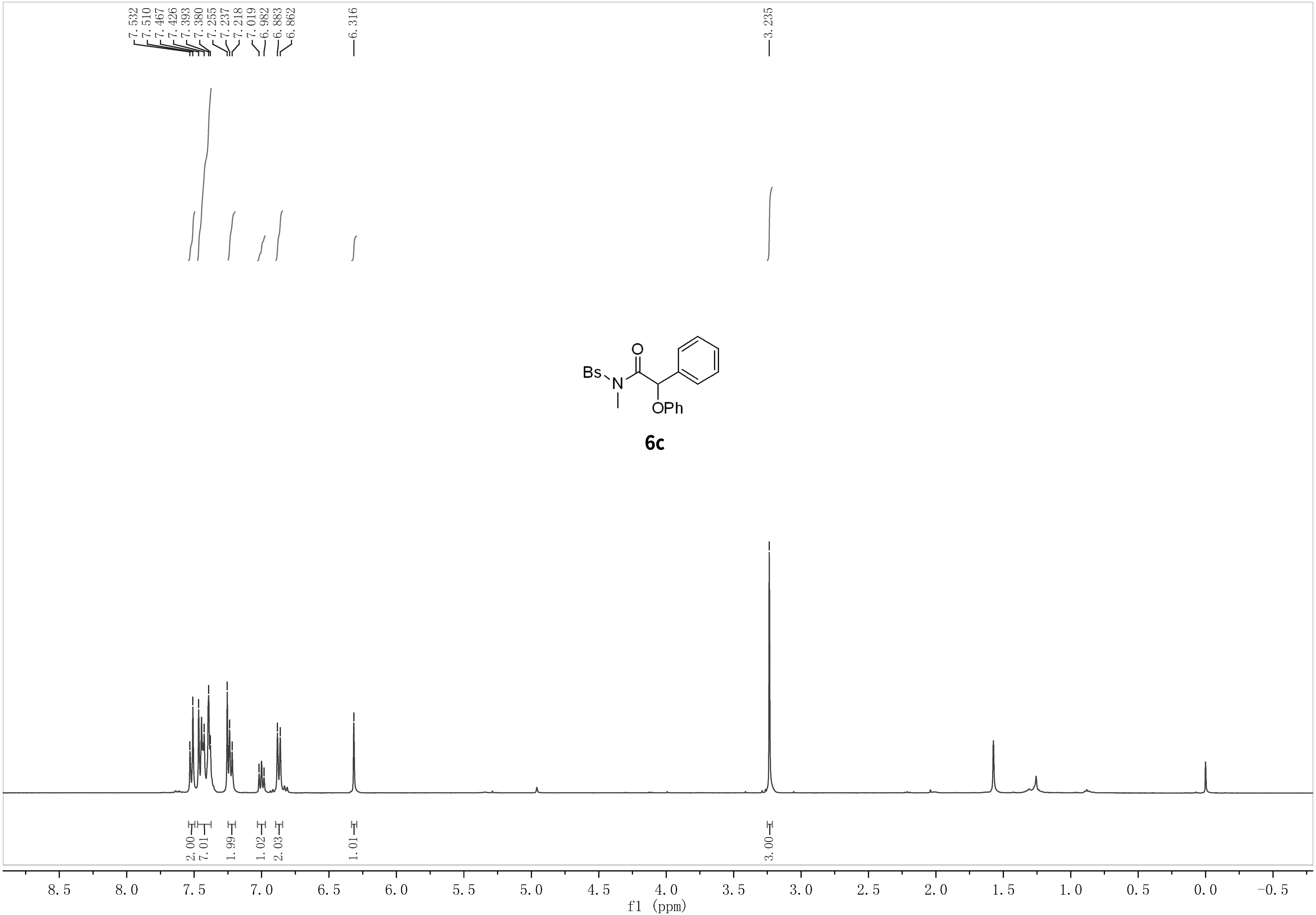
7.532
7.510
7.467
7.426
7.393
7.380
7.255
7.237
7.218
7.019
6.982
6.883
6.862
6.316
3.235
Bs
N
O
OPh
2.00
7.01
1.99
1.02
2.03
1.01
3.00
8.5
8.0
7.5
7.0
6.5
6.0
5.5
5.0
4.5
4.0
3.5
3.0
2.5
2.0
1.5
1.0
0.5
0.0
-0.5
f1 (ppm)

6c

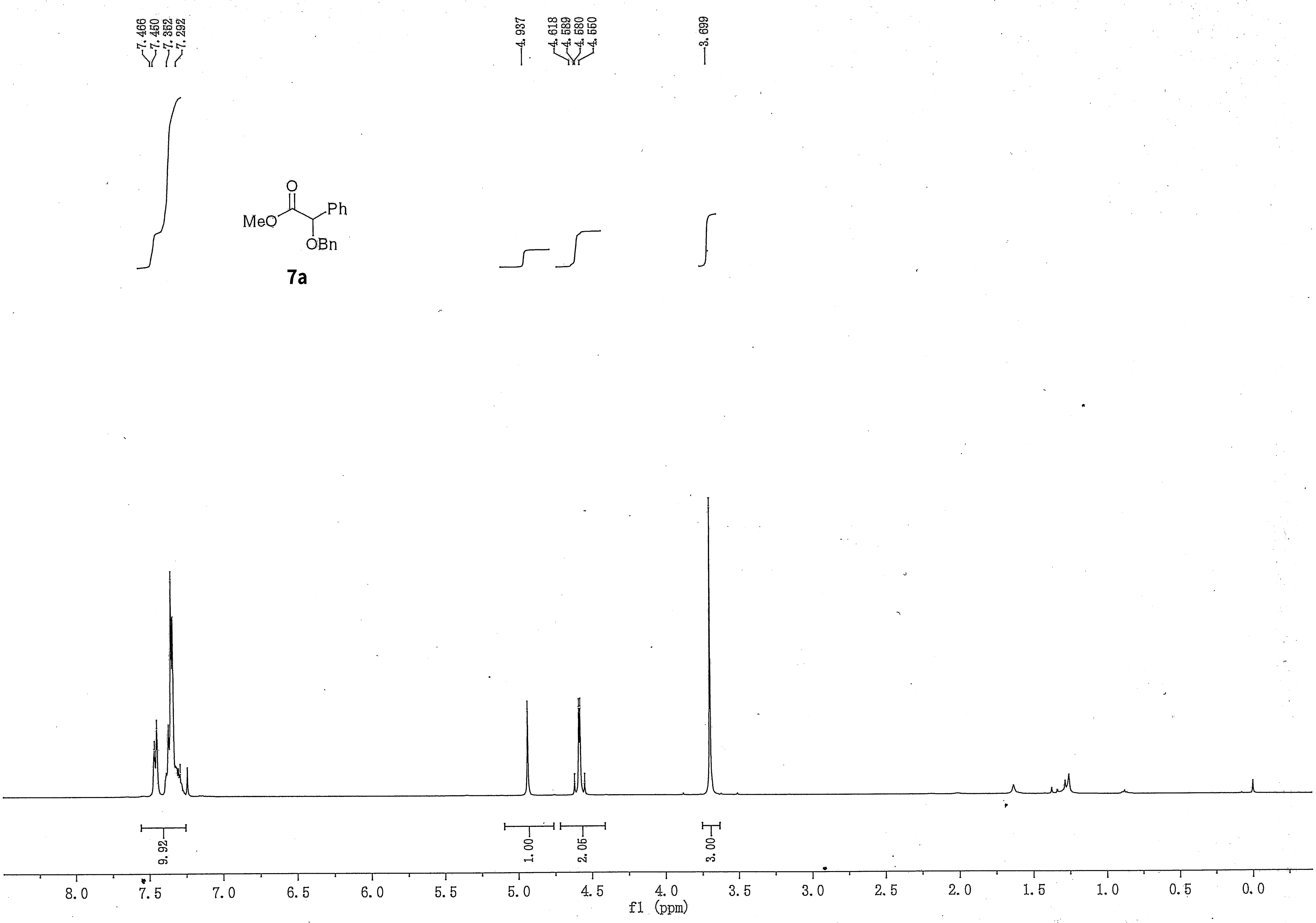

7.466
7.450
7.352
7.292
4.937
4.618
4.589
4.580
4.550
3.699
MeO
O
Ph
OBn
7a
9.92
1.00
2.05
3.00
8.0
7.5
7.0
6.5
6.0
5.5
5.0
4.5
4.0
3.5
3.0
2.5
2.0
1.5
1.0
0.5
0.0
f1 (ppm)

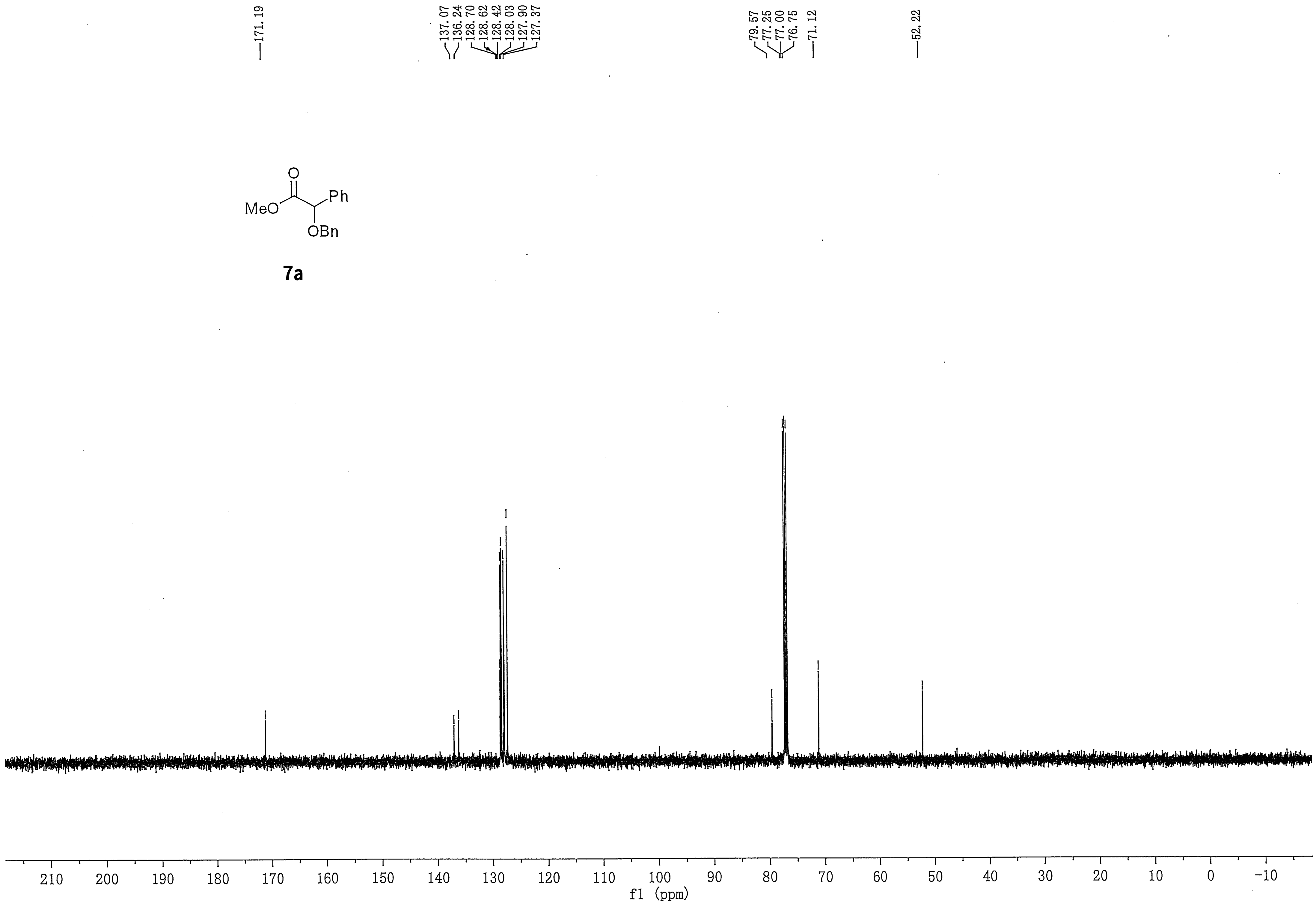

171.19
137.07
136.24
128.70
128.62
128.42
128.03
127.90
127.37
79.57
77.25
77.00
76.75
71.12
52.22
O
Ph
MeO
OBn
210 200 190 180 170 160 150 140 130 120 110 100 90 80 70 60 50 40 30 20 10 0 −10
f1 (ppm)

7a

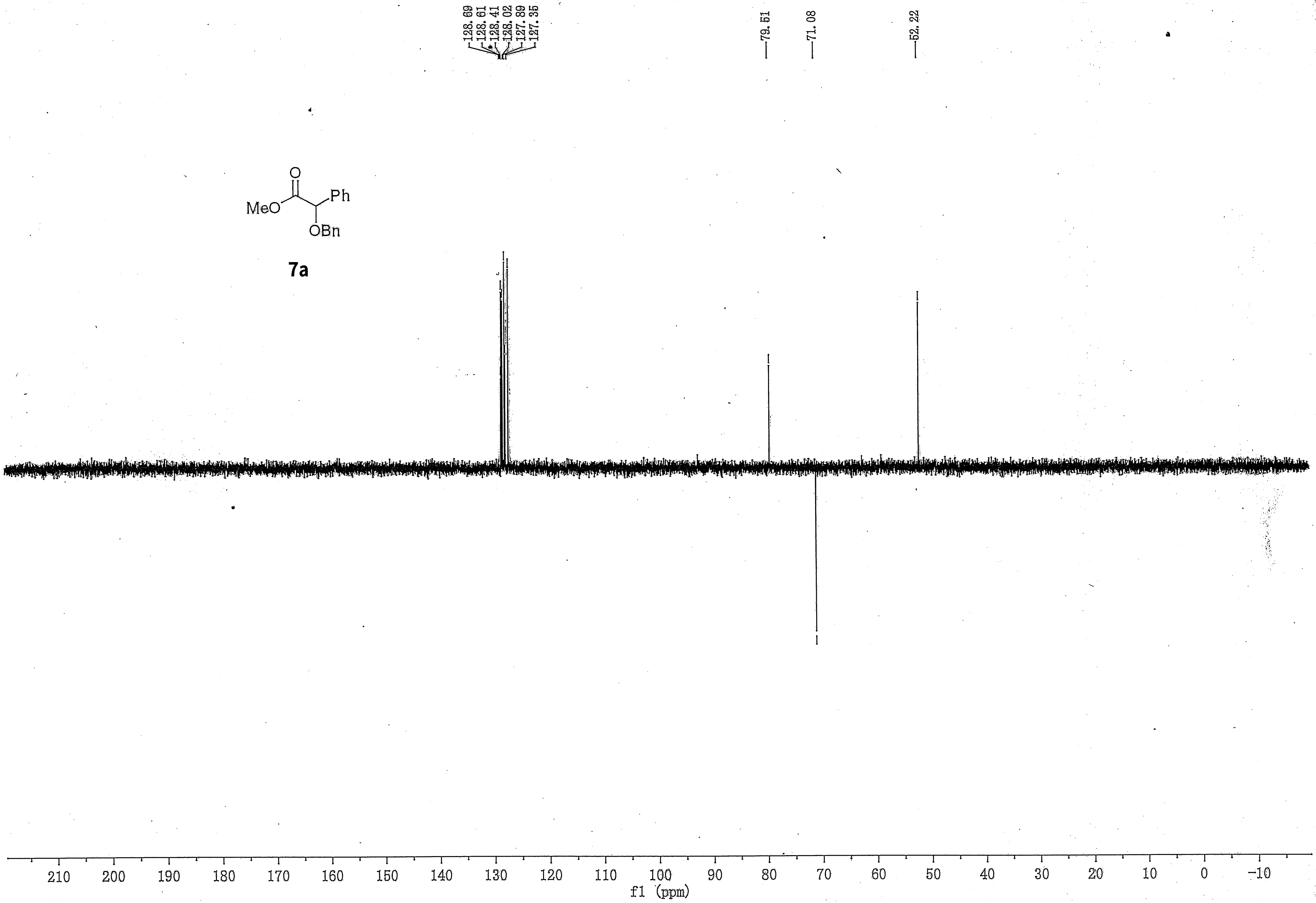
O
MeO
Ph
OBn
7a
128.69
128.61
128.41
128.02
127.89
127.35
79.51
71.08
52.22
210 200 190 180 170 160 150 140 130 120 110 100 90 80 70 60 50 40 30 20 10 0 -10
f1 (ppm)

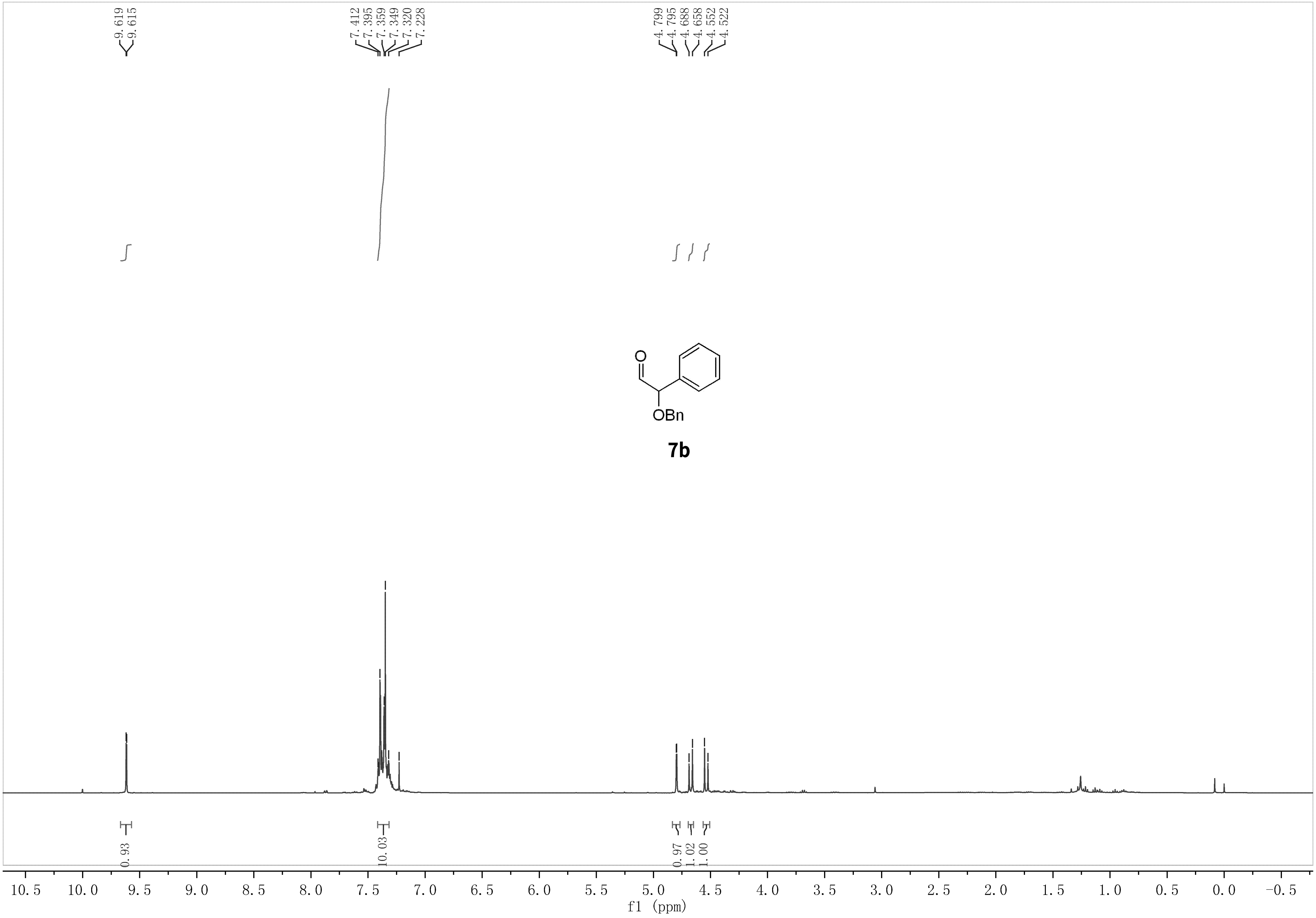
9.619
9.615
7.412
7.395
7.359
7.349
7.320
7.228
4.799
4.795
4.688
4.658
4.552
4.522
O
OBn
7b
0.93
10.03
0.97
1.02
1.00
10.5 10.0 9.5 9.0 8.5 8.0 7.5 7.0 6.5 6.0 5.5 5.0 4.5 4.0 3.5 3.0 2.5 2.0 1.5 1.0 0.5 0.0 −0.5
f1 (ppm)

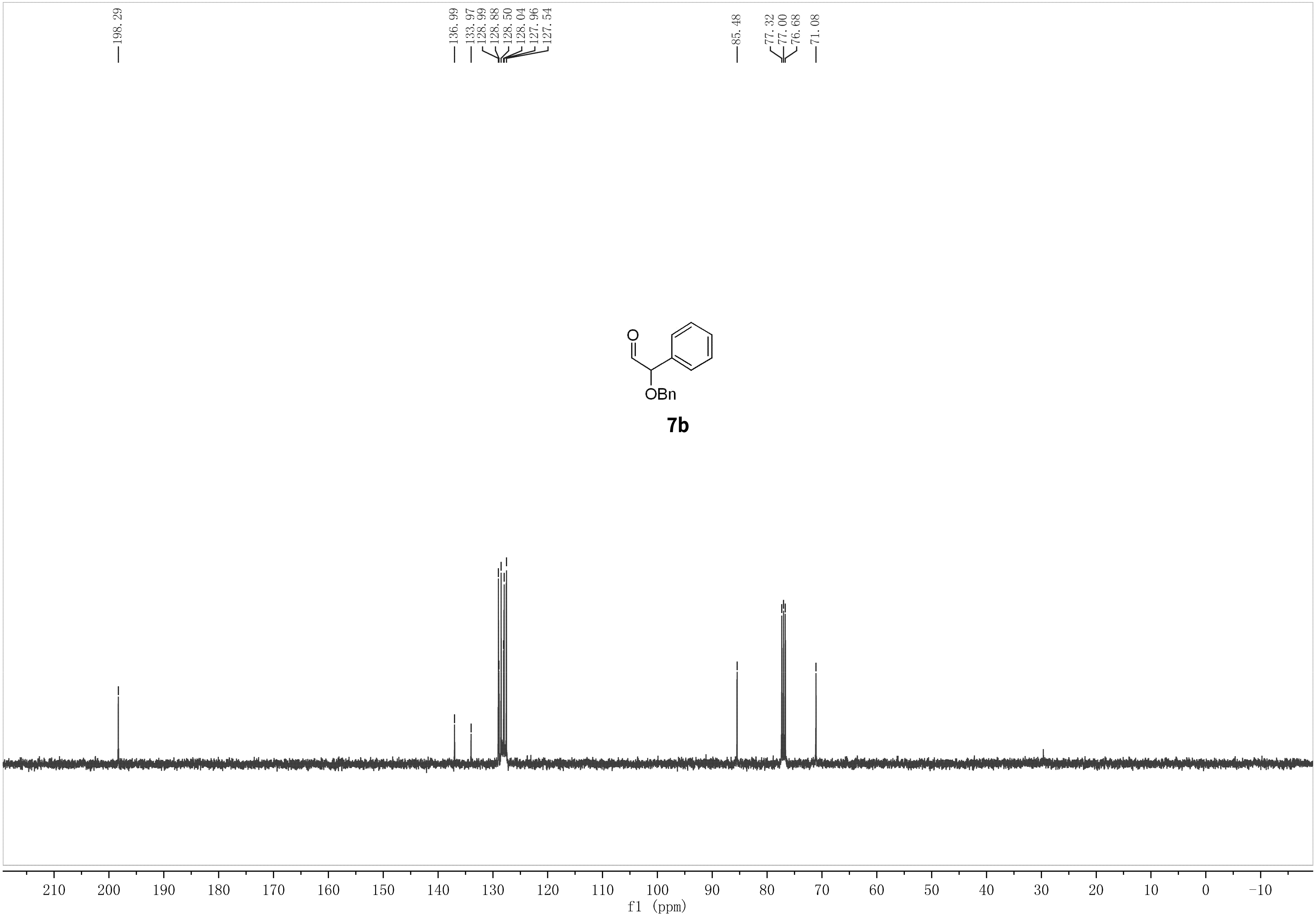
198.29
136.99
133.97
128.99
128.88
128.50
128.04
127.96
127.54
85.48
77.32
77.00
76.68
71.08
O
OBn
7b
210 200 190 180 170 160 150 140 130 120 110 100 90 80 70 60 50 40 30 20 10 0 −10
f1 (ppm)

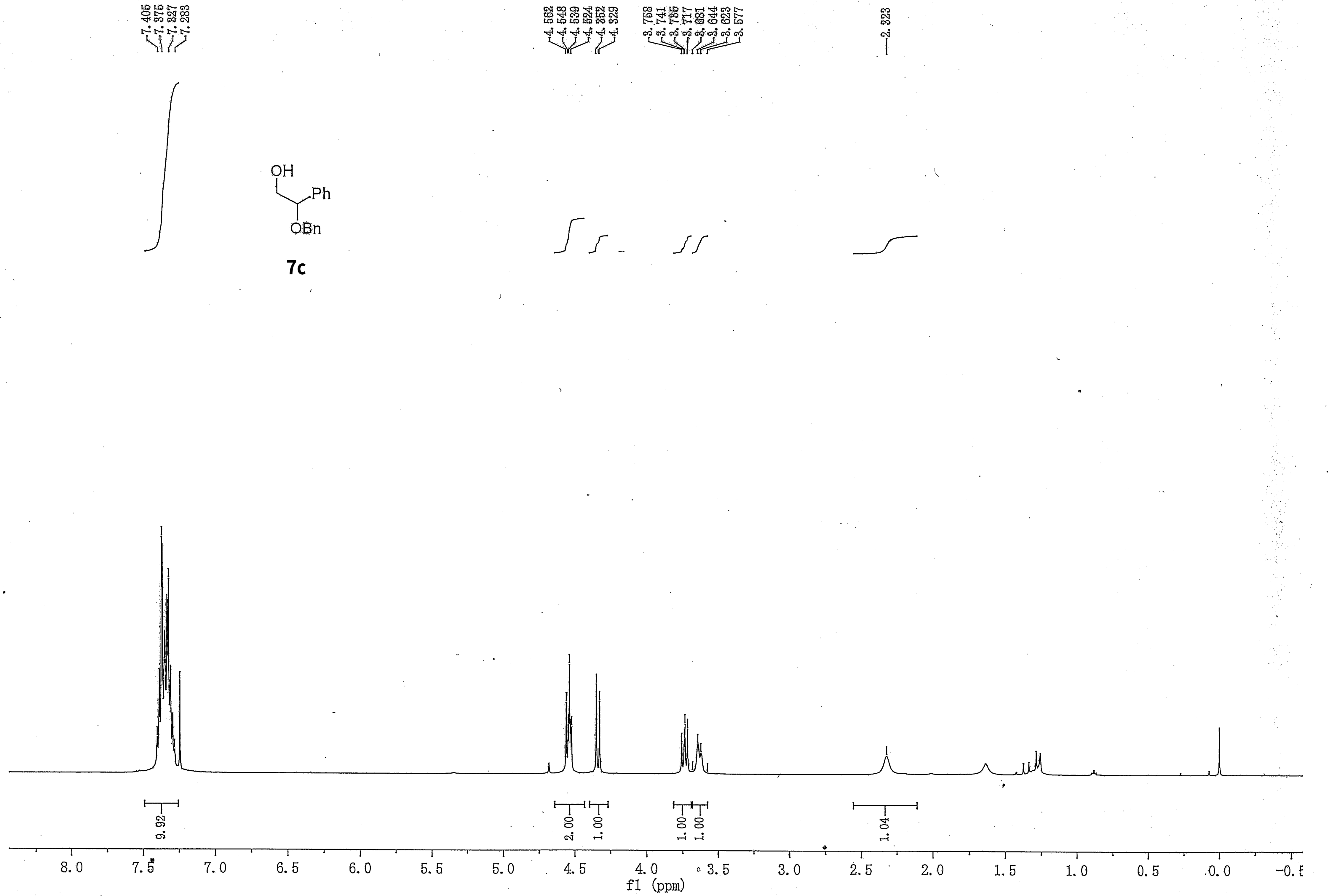
7.405
7.375
7.327
7.283
4.562
4.548
4.539
4.524
4.352
4.329
3.758
3.741
3.735
3.717
3.681
3.644
3.623
3.577
2.323
OH
Ph
OBn
7c
9.92
2.00
1.00
1.00
1.00
1.04
8.0 7.5 7.0 6.5 6.0 5.5 5.0 4.5 4.0 3.5 3.0 2.5 2.0 1.5 1.0 0.5 0.0 -0.5
f1 (ppm)

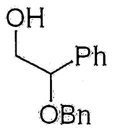
OH
Ph
OBn

7c

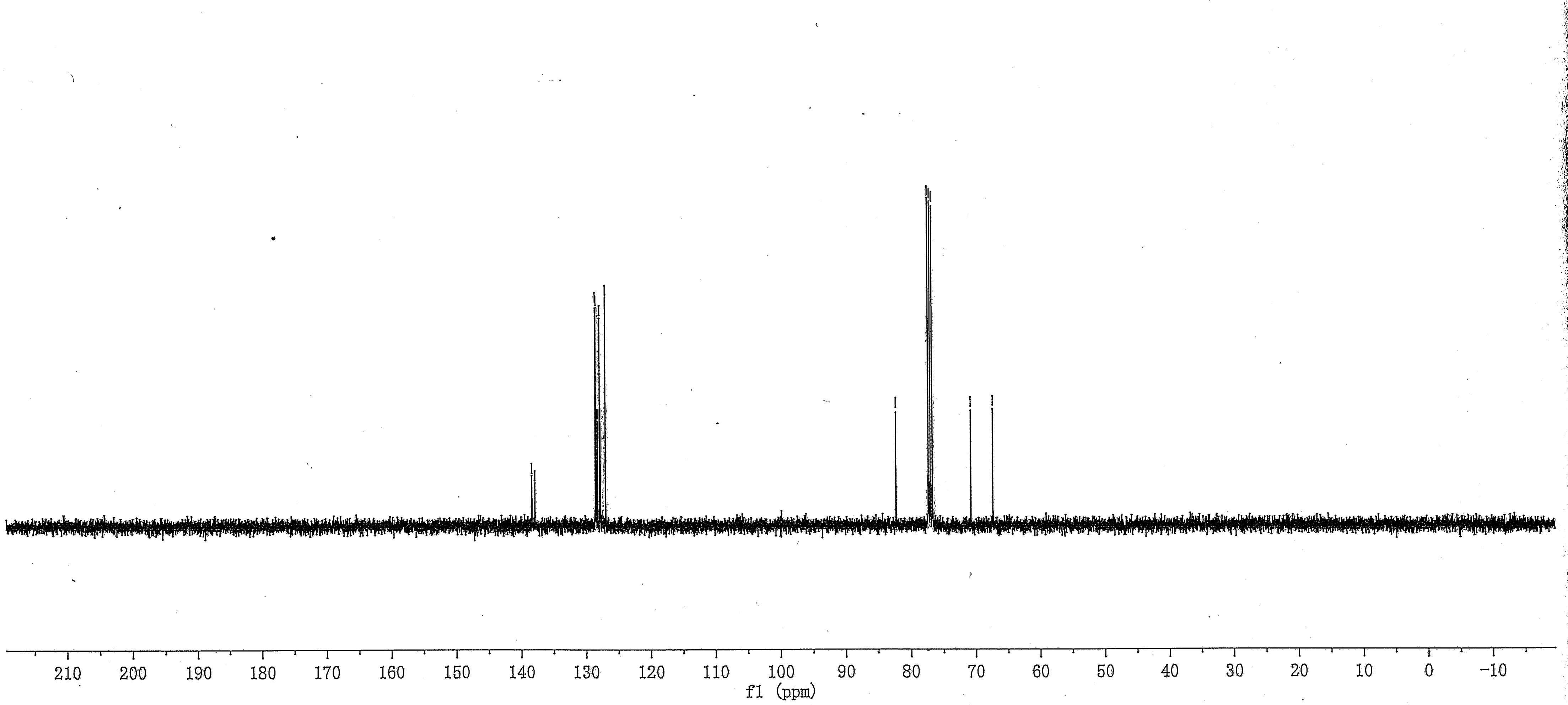
138.38
137.90
128.60
128.44
128.21
127.89
127.78
126.99
82.28
77.32
77.00
76.68
70.74
67.35
210 200 190 180 170 160 150 140 130 120 110 100 90 80 70 60 50 40 30 20 10 0 −10
f1 (ppm)

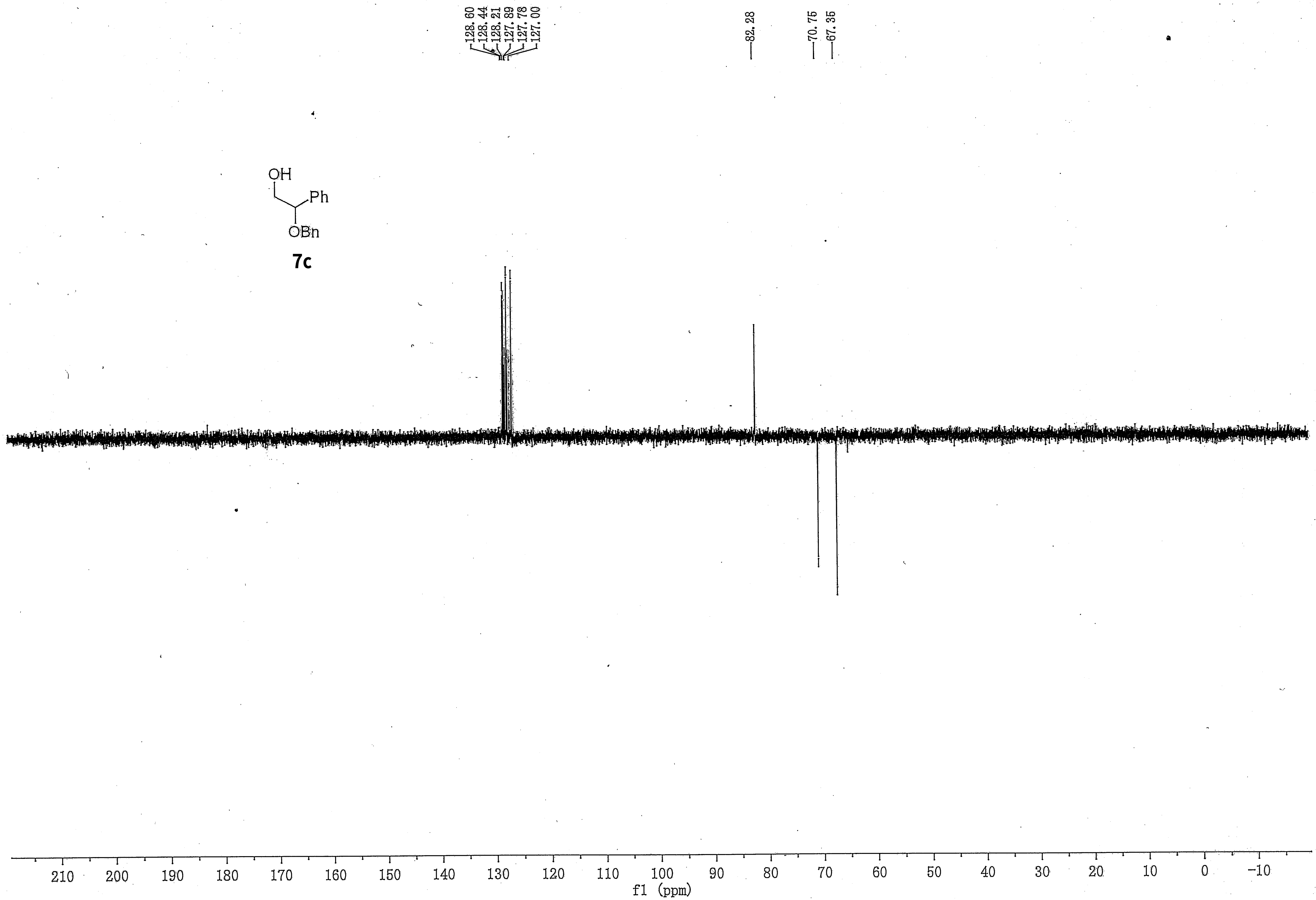
128.60
128.44
128.21
127.89
127.78
127.00
82.28
70.75
67.35
OH
Ph
OBn
210
200
190
180
170
160
150
140
130
120
110
100
90
80
70
60
50
40
30
20
10
0
-10
f1 (ppm)

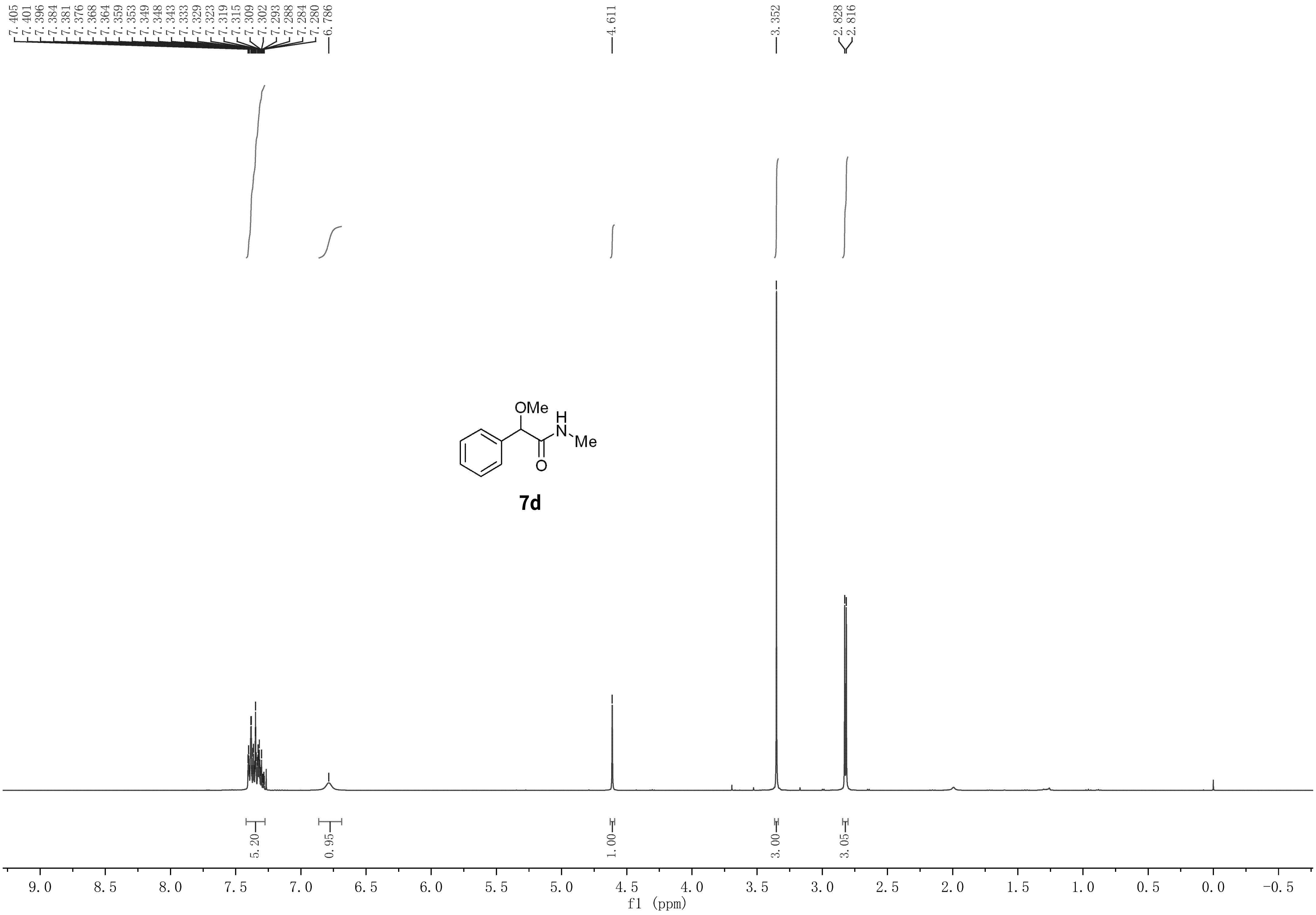
7.405
7.401
7.396
7.384
7.381
7.376
7.368
7.364
7.359
7.353
7.349
7.348
7.343
7.333
7.329
7.323
7.319
7.315
7.309
7.302
7.293
7.288
7.284
7.280
6.786
4.611
3.352
2.828
2.816
OMe
H
N
Me
O
7d
5.20
0.95
1.00
3.00
3.05
9.0
8.5
8.0
7.5
7.0
6.5
6.0
5.5
5.0
4.5
4.0
3.5
3.0
2.5
2.0
1.5
1.0
0.5
0.0
−0.5
f1 (ppm)

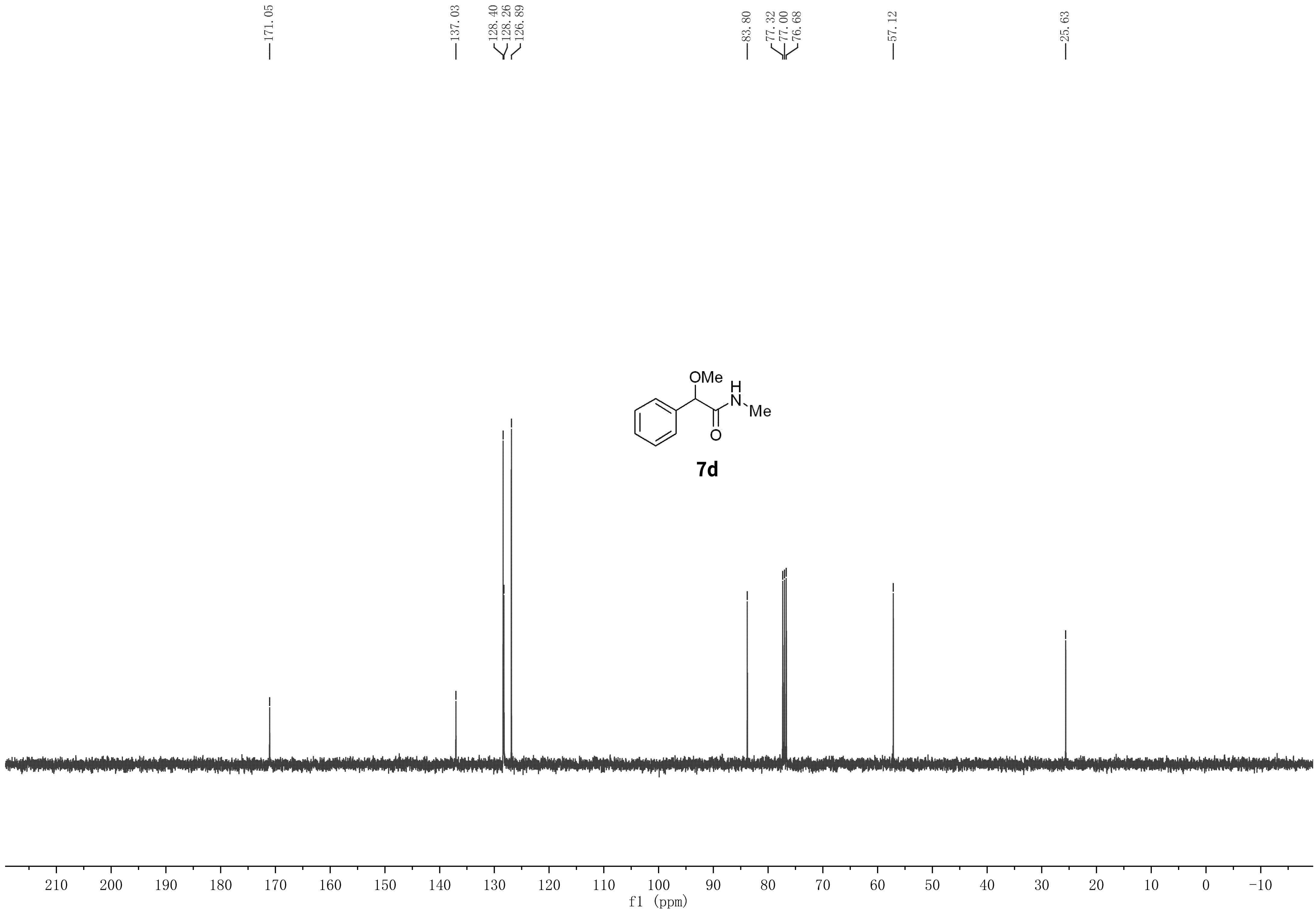
171.05
137.03
128.40
128.26
126.89
83.80
77.32
77.00
76.68
57.12
25.63
OMe
H
N
Me
O
7d
210 200 190 180 170 160 150 140 130 120 110 100 90 80 70 60 50 40 30 20 10 0 −10
f1 (ppm)

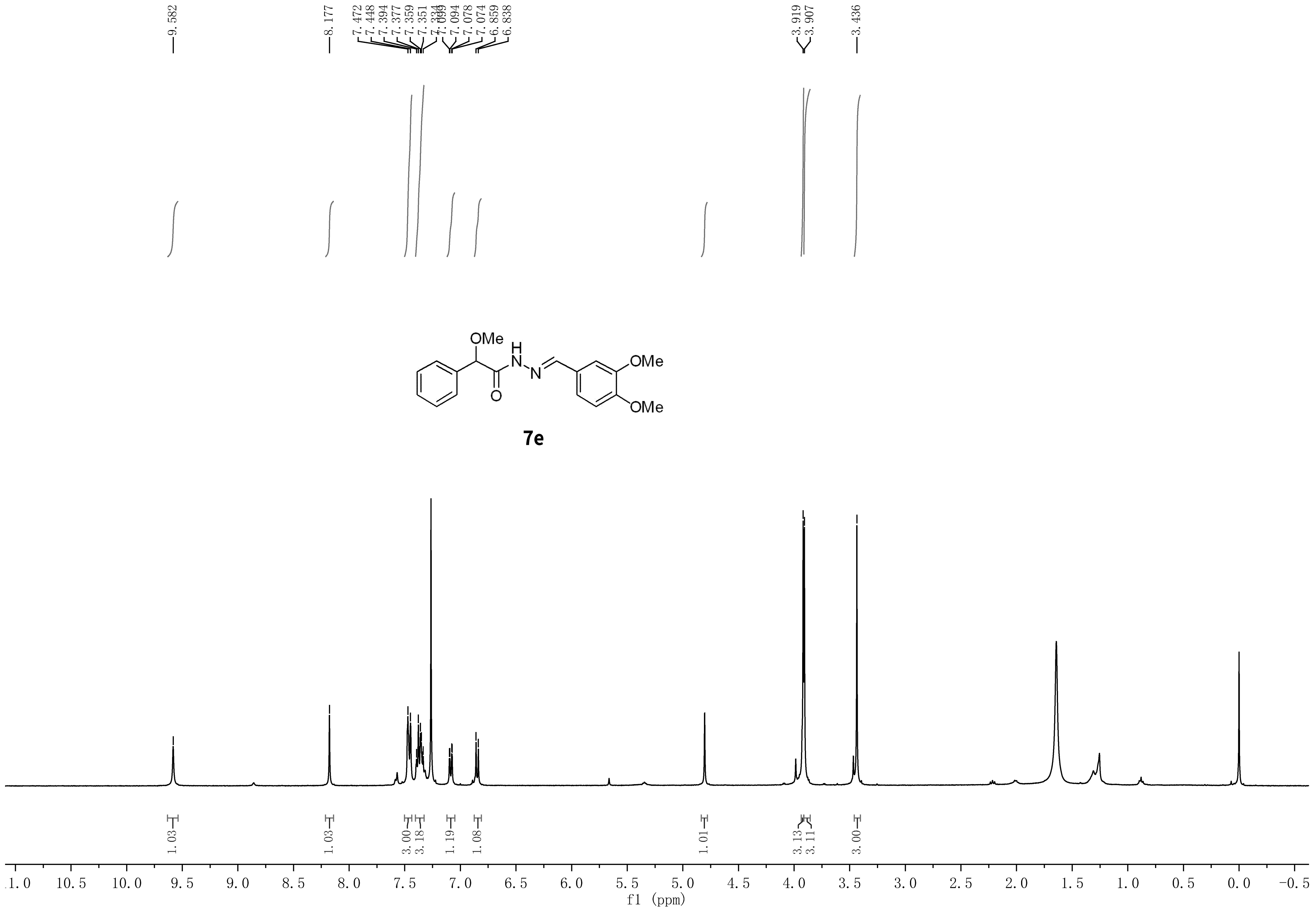

9.582
8.177
7.472
7.448
7.394
7.377
7.359
7.351
7.334
7.099
7.094
7.078
7.074
6.859
6.838
3.919
3.907
3.436
OMe
H
N
N
O
OMe
OMe
7e
1.03
1.03
3.00
3.18
1.19
1.08
1.01
3.13
3.11
3.00
11.0 10.5 10.0 9.5 9.0 8.5 8.0 7.5 7.0 6.5 6.0 5.5 5.0 4.5 4.0 3.5 3.0 2.5 2.0 1.5 1.0 0.5 0.0 -0.5
f1 (ppm)

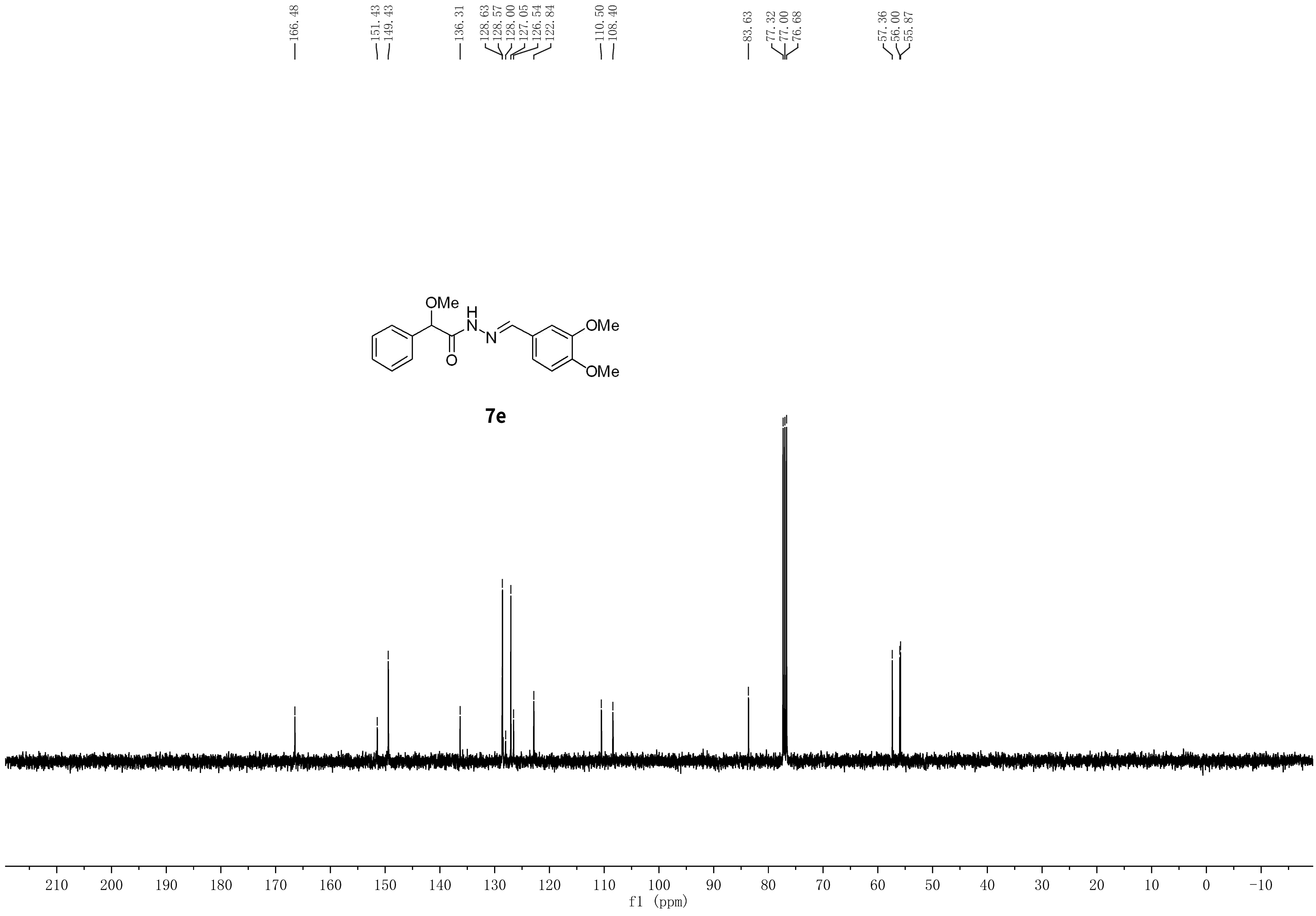
166.48
151.43
149.43
136.31
128.63
128.57
128.00
127.05
126.54
122.84
110.50
108.40
83.63
77.32
77.00
76.68
57.36
56.00
55.87
OMe
H
N
N
O
OMe
OMe
210
200
190
180
170
160
150
140
130
120
110
100
90
80
70
60
50
40
30
20
10
0
-10
f1 (ppm)

7e
